# Mutations in the Neuronal Vesicular SNARE *VAMP2* Affect Synaptic Membrane Fusion and Impair Human Neurodevelopment

**DOI:** 10.1016/j.ajhg.2019.02.016

**Published:** 2019-03-28

**Authors:** Vincenzo Salpietro, Nancy T. Malintan, Isabel Llano-Rivas, Christine G. Spaeth, Stephanie Efthymiou, Pasquale Striano, Jana Vandrovcova, Maria C. Cutrupi, Roberto Chimenz, Emanuele David, Gabriella Di Rosa, Anna Marce-Grau, Miquel Raspall-Chaure, Elena Martin-Hernandez, Federico Zara, Carlo Minetti, Deciphering Developmental Disorders Study, SYNAPS Study Group, Vincenzo Salpietro, Stephanie Efthymiou, Yamna Kriouile, Mohamed El Khorassani, Mhammed Aguennouz, Blagovesta Karashova, Daniela Avdjieva, Hadil Kathom, Radka Tincheva, Lionel Van Maldergem, Wolfgang Nachbauer, Sylvia Boesch, Larissa Arning, Dagmar Timmann, Bru Cormand, Belen Pérez-Dueñas, Gabriella Di Rosa, Erica Pironti, Jatinder S. Goraya, Tipu Sultan, Salman Kirmani, Shahnaz Ibrahim, Farida Jan, Jun Mine, Selina Banu, Pierangelo Veggiotti, Michel D. Ferrari, Alberto Verrotti, Gian Luigi Marseglia, Salvatore Savasta, Barbara Garavaglia, Carmela Scuderi, Eugenia Borgione, Valeria Dipasquale, Maria Concetta Cutrupi, Simona Portaro, Benigno Monteagudo Sanchez, Mercedes Pineda-Marfa’, Francina Munell, Alfons Macaya, Richard Boles, Gali Heimer, Savvas Papacostas, Andreea Manole, Nancy Malintan, Maria Natalia Zanetti, Michael G. Hanna, James E. Rothman, Dimitri M. Kullmann, Henry Houlden, Oscar D. Bello, Rita De Zorzi, Sara Fortuna, Andrew Dauber, Mariam Alkhawaja, Tipu Sultan, Kshitij Mankad, Antonio Vitobello, Quentin Thomas, Frederic Tran Mau-Them, Laurence Faivre, Francisco Martinez-Azorin, Carlos E. Prada, Alfons Macaya, Dimitri M. Kullmann, James E. Rothman, Shyam S. Krishnakumar, Henry Houlden

**Affiliations:** 1 Pediatric Neurology and Muscular Diseases Unit, IRCCS Istituto Giannina Gaslini, Genoa 16147, Italy; 2 Department of Neurosciences, Rehabilitation, Ophthalmology, Genetics, Maternal and Child Health, University of Genoa, Genoa 16132, Italy; 3 Department of Molecular Neuroscience, UCL Institute of Neurology, University College London, London WC1N 3BG, UK; 4 Department of Clinical and Experimental Epilepsy, UCL Institute of Neurology, University College London, London WC1N 3BG, UK; 5 Department of Medical Genetics, Hospital Universitario Cruces, Greater Bilbao 48903, Spain; 6 Division of Human Genetics, Department of Pediatrics, University of Cincinnati, Cincinnati Children's Hospital Medical Center, Cincinnati, Ohio 45229-3026, USA; 7 Division of Human Genetics, Department of the Adult and Developmental Age Human Pathology, University of Messina, Messina 98125, Italy; 8 Papardo University Hospital, Viale Ferdinando Stagno d'Alcontres, Contrada Papardo, Messina 98158, Italy; 9 Division of Child Neurology and Psychiatry, Department of the Adult and Developmental Age Human Pathology, University of Messina, Messina 98125, Italy; 10 Department of Pediatric Neurology, University Hospital Vall d'Hebron, Barcelona 08035, Spain; 11 Unidad de Enfermedades Mitocondriales-Metabólicas Hereditarias, Departamento de Pediatría, Hospital 12 de Octubre, Madrid 28041, Spain; 12 Laboratory of Neurogenetics and Neuroscience, G. Gaslini Institute, Genova 16147, Italy; 13 Center of Excellence in Biocrystallography, Department of Chemical and Pharmaceutical Sciences, University of Trieste, Trieste 34127, Italy; 14 Department of Chemical and Pharmaceutical Sciences, University of Trieste, Trieste 34127, Italy; 15 Division of Endocrinology, Cincinnati Center for Growth Disorders, Cincinnati Children's Hospital Medical Center, Cincinnati, Ohio 45229-3026, USA; 16 Prince Hamzah Hospital, Ministry of Health, Amman 11181, Jordan; 17 Department of Pediatric Neurology, Institute of Child Health and The Children’s Hospital Lahore, 381-D/2, Lahore 54600, Pakistan; 18 Department of Neuroradiology, Great Ormond Street Hospital for Children, London WC1N 3JH, UK; 19 Unité Fonctionnelle Innovation en Diagnostic Genomique des Maladies Rares, Center Hospitalier Universitaire Dijon Bourgogne, Dijon 21079, France; 20 Inserm, UMR 1231, Genetique des Anomalies du Development, Université de Bourgogne, Dijon 21079, France; 21 Center de Référence Anomalies du Développement et Syndromes Malformatifs, Hôpital d'Enfants, Dijon 21079, France; 22 Centro de Investigación Biomédica en Red de Enfermedades Raras (CIBERER), Instituto de Investigación Hospital 12 de Octubre (i+12), Madrid 28041, Spain; 23 Department of Cell Biology, Yale University School of Medicine, New Haven, CT 06520, USA

**Keywords:** SNARE, vesicle fusion, neuronal exocytosis, VAMP2, synaptobrevin, neurodevelopmental disorders, synaptopathy, autism, movement disorders, epilepsy

## Abstract

*VAMP2* encodes the vesicular SNARE protein VAMP2 (also called synaptobrevin-2). Together with its partners syntaxin-1A and synaptosomal-associated protein 25 (SNAP25), VAMP2 mediates fusion of synaptic vesicles to release neurotransmitters. VAMP2 is essential for vesicular exocytosis and activity-dependent neurotransmitter release. Here, we report five heterozygous *de novo* mutations in *VAMP2* in unrelated individuals presenting with a neurodevelopmental disorder characterized by axial hypotonia (which had been present since birth), intellectual disability, and autistic features. In total, we identified two single-amino-acid deletions and three non-synonymous variants affecting conserved residues within the C terminus of the VAMP2 SNARE motif. Affected individuals carrying *de novo* non-synonymous variants involving the C-terminal region presented a more severe phenotype with additional neurological features, including central visual impairment, hyperkinetic movement disorder, and epilepsy or electroencephalography abnormalities. Reconstituted fusion involving a lipid-mixing assay indicated impairment in vesicle fusion as one of the possible associated disease mechanisms. The genetic synaptopathy caused by *VAMP2 de novo* mutations highlights the key roles of this gene in human brain development and function.

## Main Text

Chemical synaptic transmission relies on precisely coordinated, activity-dependent neurotransmitter release.^1^ A fundamental step in this pathway is the fusion of synaptic vesicles with the presynaptic plasma membrane. Soluble N-ethylmaleimide-sensitive factor attachment protein receptor (SNARE) proteins mediate membrane fusion and are essential for the fusion of synaptic vesicles.^1^, ^2^ At mammalian central nervous system (CNS) synapses, neuronal SNAREs consist of vesicle-associated membrane protein 2 (VAMP2, also called synaptobrevin-2) on the vesicle membrane (v-SNARE) and the binary complex of syntaxin1A (STX1A) and synaptosomal-associated protein 25 Kd (SNAP25) on the plasma membrane (target or t-SNARE).^3^ The v- and t-SNARE proteins assemble in a polarized manner starting from the N termini distal from the membranes and proceeding towards the C termini and are held together by discrete interacting residues (numbered -7 to +8), including 15 hydrophobic contacts and central ionic residues.^4^ This “zippering” process pulls the membranes together and provides the energy to fuse the lipid bilayers.^5^, ^6^ The SNAREs alone are sufficient to drive fusion of synaptic vesicles, but this process is tightly regulated by a number of synaptic proteins to enable Ca^2+^-regulated neurotransmitter release.^7^ The key regulatory elements at excitatory CNS synapses include chaperones (Munc18 and Munc13), the primary Ca^2+^ sensor synaptotagmin-1, and the auxiliary protein complexin.^7^, ^8^, ^9^, ^10^

*VAMP2* (MIM: 185881) encodes a neuronal v-SNARE essential for the fusion of synaptic vesicles at mammalian central nerve terminals.^5^, ^6^, ^7^ Introduction of specific engineered mutations affecting its SNARE motif has been reported to alter vesicle fusion *in vitro* by impairing either formation of the SNARE complex or the interaction of VAMP2 with other (auxiliary) presynaptic proteins.^11^, ^12^ *Vamp2*^−/−^ mice present severely decreased rates of both spontaneous and Ca^2+^-triggered synaptic-vesicle fusion, and these mice die immediately after birth.^13^ Also, synapses from VAMP2-deficient mice display changes in synaptic-vesicle morphology and size—and delayed stimulus-dependent endocytosis.^14^ Thus, VAMP2 exerts a complex influence on synaptic transmission; it plays fundamental roles in vesicle fusion, neurotransmitter release, and vesicle endocytosis. Despite the critical role of VAMP2 in presynaptic molecular events, little is known of the consequences of disrupted *VAMP2* function in human neurodevelopment.

Here, we describe five unrelated individuals who had shown hypotonia since birth and who had intellectual disability (ID) with autistic features, including variable motor stereotypies resembling Rett syndrome (RTT), and, in some children, also central visual impairment, hyperkinetic movements, and epilepsy and/or electroencephalography (EEG) abnormalities. Table 1 summarizes the detailed phenotypes of the individuals (1–5), aged between 3 and 14 years.

**Table 1:** Clinical Features of Individuals with *De Novo VAMP2* Mutations

**Individual Number Gender Age**	**Variant**	**Growth/OFC**	**Hypotonia/DD**	**ID**	**Epileptic Seizures**	**EEG**	**ASD**	**RTT-Like Features**	**Movement Disorder**	**Central Visual Defects**	**Speech Impairment**	**Brain Imaging**	**Additional Features**
1 F 3 yr	c.223T>C, p.Ser75Pro	normal	yes	severe	no	high-voltage delta activity, sharp wave-slow wave complexes	yes	stereotyped hand movements, absent purposeful hand movements	choreic movement, flapping, dystonic postures	yes	absent speech	thin corpus callosum, delayed myelination	inability to walk
2 M 10 yr	c.233A>C, p.Glu78Ala	normal	yes	severe	focal seizures, GTCS	fast rhythmic activity, sharp wave-slow wave complexes	yes	body rocking, head banging, screaming, absent purposeful hand movements	generalized chorea	yes	absent speech	unremarkable	abnormal behavior, self-injury, inability to walk
3 M 13yr	c.230T>C, p.Phe77Ser	normal	yes	severe	infantile spasms, convulsive status epilepticus	disorganized EEG paroxysms	yes	stereotyped hand movements, absent purposeful hand movements	choreic movement, myoclonic jerks	yes	absent speech	unremarkable	abnormal behavior, inability to walk, severe constipation
4 M 14yr	c.128_130delTGG, p.Val43del	normal	yes	moderate	focal seizures	generalized and multifocal abnormalities	yes	stereotyped hand movements (wringing), absent purposeful hand movements	no	no	only 5–10 spoken words	unremarkable	clumsiness, abnormal behavior
5 F 3 yr	c.135_137delCAT, p.Ile45del	normal	yes	moderate	no	disorganized EEG paroxysms	yes	stereotyped hand movements (washing)	no	no	only 5 spoken words	unremarkable	abnormal behavior

Abbreviations are as follows: ASD = autism spectrum disorder; DD = developmental delay; EEG = electroencephalography; FC = focal seizures; GTCS = generalized tonic-clonic seizures; ID = intellectual disability; and OFC = occipital-frontal circumference. Variants are named according to the GenBank: NM_014232 reference transcript.

In all affected children, family histories, pregnancies, and birth histories were unremarkable, and neurodevelopmental impairment occurred within the first year of life. The earliest sign of neurological involvement was axial hypotonia at birth. Poor visual fixation (with only brief and occasional visual contact, lasting up to a few seconds) had been evident since the first months of life in three affected individuals (1–3); these individuals were later diagnosed with central visual impairment (Table 1). Three children (individuals 1–3) exhibited a hyperkinetic movement disorder starting in the first year of life (Videos S1, S2, S3, and S4). Abnormal movements ranged from dystonic posturing (mainly involving the trunk, neck, and lower limbs) and moderate chorea (individuals 1 and 3) to a mixed-movement disorder with severe chorea and dystonic posturing (individual 2) or myoclonic jerks (individual 3). All children showed autistic features, typically including flapping or flailing of the arms, as well as hand wringing or clapping. Additional repetitive behavior patterns included body rocking and head banging. Self-injurious behaviors were evident in individual 2. A virtual absence of purposeful hand movements was present in all cases (Table 1, Videos S1, S2, S3, S4, and S5). Motor development in individuals 1–3 was severely impaired, and these children had not attained the ability to walk. Severe language impairment was present in the three more severely affected children (individuals 1–3), none of whom had attained meaningful speech production, but individuals 4 and 5 were capable of saying 5–10 words (Table 1).

Video S1. Individual 1 at the Ages of 2, 5, and 6 Months Individual 1, with the *de novo* p.Ser75Pro variant, presented with hypotonia at the age of 2 months; at the age of 5 months, she showed staring, a hyperkinetic movement disorder, flapping, hand stereotypies, and automatisms (hand to mouth); at the age of 6 months, when the individual is in the pushchair, note the virtual absence of purposeful hand movements.

Video S2. Individual 1 at the Age of 15 Months Individual 1, with the *de novo* p.Ser75Pro variant, presented with hypotonia, developmental delay, an inability to attain sitting position, poor visual contact, flapping, hand stereotypies (washing), choreic-predominant movement disorder, dystonic postures, staring, and automatisms (hand to mouth).

Video S3. Individual 2 at the Age of 6 Years Individual 2, with the *de novo* p.Glu78Ala variant, at the age of 6 years presented with hypotonia, developmental delay, lack of purposeful hand movements, automatisms (hand to mouth), backward falling, and dystonic posturing of the four limbs, followed by severe generalized choreic movements.

Video S4. Individual 3 at the Age of 12 Years Individual 3, with the *de novo* p.Phe77Ser, presented with hypotonia, developmental delay, poor visual contact, a virtual absence of purposeful hand movements, stereotypies (hand clapping), and generalized choreic movements with myoclonic jerks.

Video S5. Individual 4 at the Age of 14 Years Individual 4, with the *de novo* p.Val43del variant, at the age of 14 years presented some motor clumsiness and hand stereotypies (washing, clapping).

**Figure 1:**
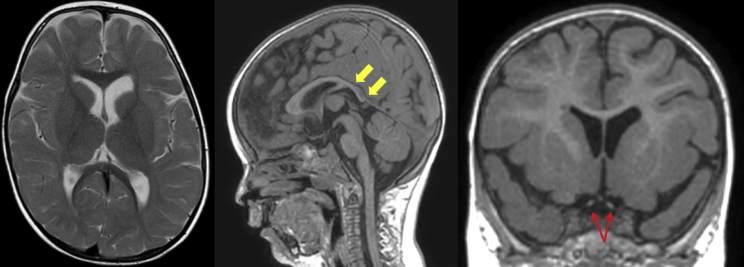
Brain MRI Scan of Individual 1, Who Harbors a *De Novo* VAMP2 p.Ser75Pro Variant, at the Age of 2 Years The panel shows axial T2-weighted, sagittal T1-weighted, and coronal T1-weighted MR images. There is some generalized delay in the maturation of myelin and a reduced volume of the cerebral white matter posteriorly. (Yellow arrows show a posteriorly slender corpus callosum.) The optic nerves and chiasm are hypoplastic (red arrows).

Seizures or abnormal EEG occurred in four affected individuals. Individual 1 did not present with epileptic seizures, but ictal EEG recording at the age of 15 months showed high-voltage delta activity with interspersed sharp-and-slow-wave complexes over the right central and posterior brain regions. Individual 2 suffered from multiple focal seizures per day; these started shortly after birth and were characterized on EEG by fast rhythmic activity followed by sharp-and-slow-wave complexes (Figure S1). At 12 months, individual 3 presented with infantile spasms that were associated with diffuse EEG paroxysms. Individual 4 developed infrequent staring episodes with eyelid myoclonia at 5 years of age and had a single episode of non-convulsive *status epilepticus* at the age of 11 years. Several anti-epileptic drugs, including valproic acid, vigabatrin, and lamotrigine, have been trialed in individuals 2–4 (see Supplemental Data); beneficial effects of valproic acid treatment were noted in individual 4, who has been seizure-free since the age of 12 years and has had normal follow-up EEGs. Individual 2 underwent a craniotomy for grid placement at the age of 6 months and had a right posterior circulation stroke affecting the thalamic and cortical areas; at the age of 18 months, he had a right temporal lobectomy. Brain magnetic resonance imaging (MRI) was unrevealing in all children except in individual 1, for whom mild myelination delay and a posteriorly slender corpus callosum was observed at the age of 2 years (Figure 1).

The clinical features summarized above are consistent with a diagnosis of neurodevelopmental impairment with variable neurological features in all five affected individuals. Extensive initial genetic and biochemical diagnostic investigations for a range of genetic conditions, including non-syndromic ID, epileptic encephalopathies (EEs), EEs with dyskinesia, metabolic disorders, and mitochondrial diseases, were unrevealing (see Supplemental Data). Affected children were recruited for genetic analysis through the use of whole-exome sequencing (WES) at five centers. Written informed consent was obtained for all individuals and their relatives, after which DNA was extracted from peripheral lymphocytes according to standard protocols. The study was approved by the local ethics committee at University College London Hospitals (project 06/N076) and at the participating institutions. Variants of interest in *VAMP2* were identified by WES of trios and confirmed by Sanger sequencing in all cases. Libraries were prepared from parents’ and affected individuals’ DNA, and exomes were captured and sequenced on Illumina sequencers. Raw data were processed and filtered with established pipelines and then annotated, and the Exome variant server ESP6500 was used for assessments of variant frequency in the control population (see Supplemental Data). Only exonic and donor and acceptor splicing variants were considered. Priority was given to rare variants (that had a genomic evolutionary rate profiling [GERP] score >2 and were present at <1% in public databases, including those of the 1000 Genomes Project, NHLBI Exome Variant Server, Complete Genomics 69, and Exome Aggregation Consortium [ExAC v0.2]). Synonymous variants were not considered. Following their respective analysis pipelines,^15^, ^16^, ^17^, ^18^ participating centers generated a list of candidate variants filtered against variants from public databases according to modes of inheritance, then compared their results through international research networks and variant databases.^19^, ^20^

**Figure 2:**
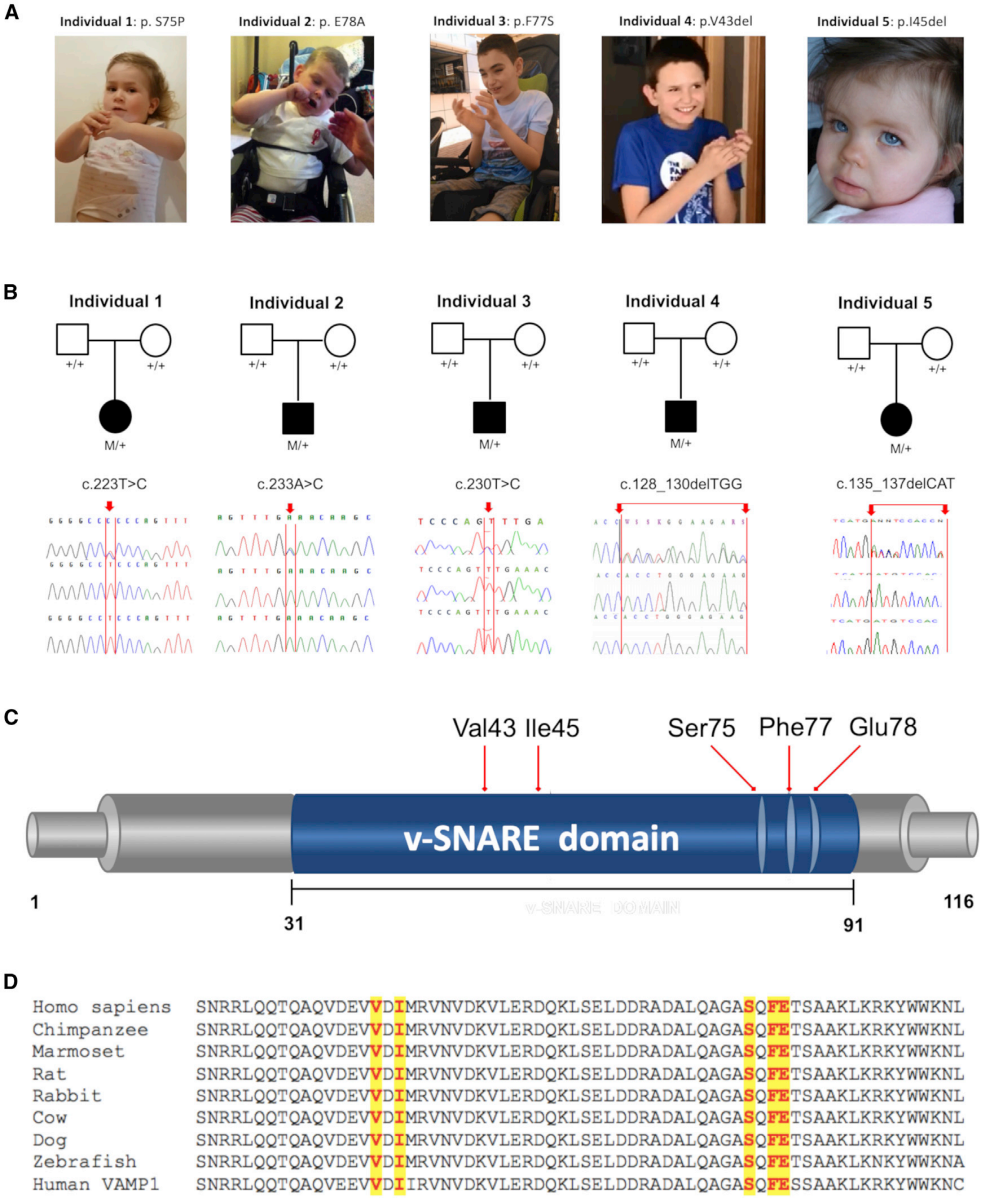
*VAMP2* Intragenic *De Novo* Variants Identified in This Study (A) Individuals carrying *de novo VAMP2* intragenic variants; note the hand stereotypies. (B) Sanger sequences of five kindreds with *de novo VAMP2* intragenic variants. Chromatograms of individuals 1–5 and their parents confirm the *de-novo* occurrence of the *VAMP2* variants in all cases. M/+ denotes the indicated *VAMP2* variant in the heterozygous state, and +/+ denotes homozygous wild-type sequence. Mutant bases in the probands are indicated by a red arrow. (C) Schematic depiction of the human VAMP2 protein (GenBank: NP_055047.2) indicating the positions of the variants identified in this study. (D) Multiple alignment showing complete conservation across species and VAMP1 homolog (GenBank: NP_055046.1) of the residues affected by the variants identified in this study (these variants are highlighted in yellow). Human VAMP2 (GenBank: NP_055047.2), chimpanzee VAMP2 (UniProt: JAA33755.1), marmoset VAMP2 (UniProt: JAB33896.1), rat VAMP2 (NP_036795.1), rabbit VAMP2 (XP_008268978.1), cow VAMP2 (GenBank: NP_776908.1), dog VAMP2 (GenBank: XP_005620068.1), zebrafish VAMP2 (GenBank: NP_956299.1).

Three *de novo* non-synonymous variants in *VAMP2* [NM_014232: c.223T>C (p.Ser75Pro), c.230T>C (p.Phe77Ser), c.233A>C (p.Glu78Ala)] were identified in three affected individuals (1–3) recruited and studied at different centers as part of different research initiatives (see Supplemental Data). We then analyzed the genetic data from the SYNaPS Study Group collection of exomes and genomes from over 4,000 individuals affected with early-onset neurological disorders (including ∼250 children with undiagnosed neurodevelopmental impairment and epilepsy) for variants in *VAMP2* and identified a child (individual 4), carrying a *de novo* single amino acid deletion at position 43 [NM_014232: c.128_130delTGG (p.Val43del)] (Figures 2A and 2B). We next used web-based tools^19^, ^20^ to screen *VAMP2* variants within exome and genome datasets from established international collaborations; this process identified an additional child (individual 5) carrying a *de novo* single-amino-acid deletion at position 45 [GenBank: NM_014232, c.135_137delCAT (p.Ile45del)] (see Supplemental Data).

All the identified variants were absent from the Genome Aggregation Database and ExAC, and all displayed high conservation (mean: GERP^++^ 5.26) and *in silico* pathogenic predictor (mean: CADD_Phred 26.9) scores (see Supplemental Data). In the ExAC database (last accessed January 30, 2018), which contains exomes from 60,706 unrelated individuals, there are no listed loss-of-function variants in *VAMP2,* and only two non-synonymous variants (p.Asn49Lys [p.Val50Met]) are present within the SNARE motif (amino acids 31–91).

**Figure 3:**
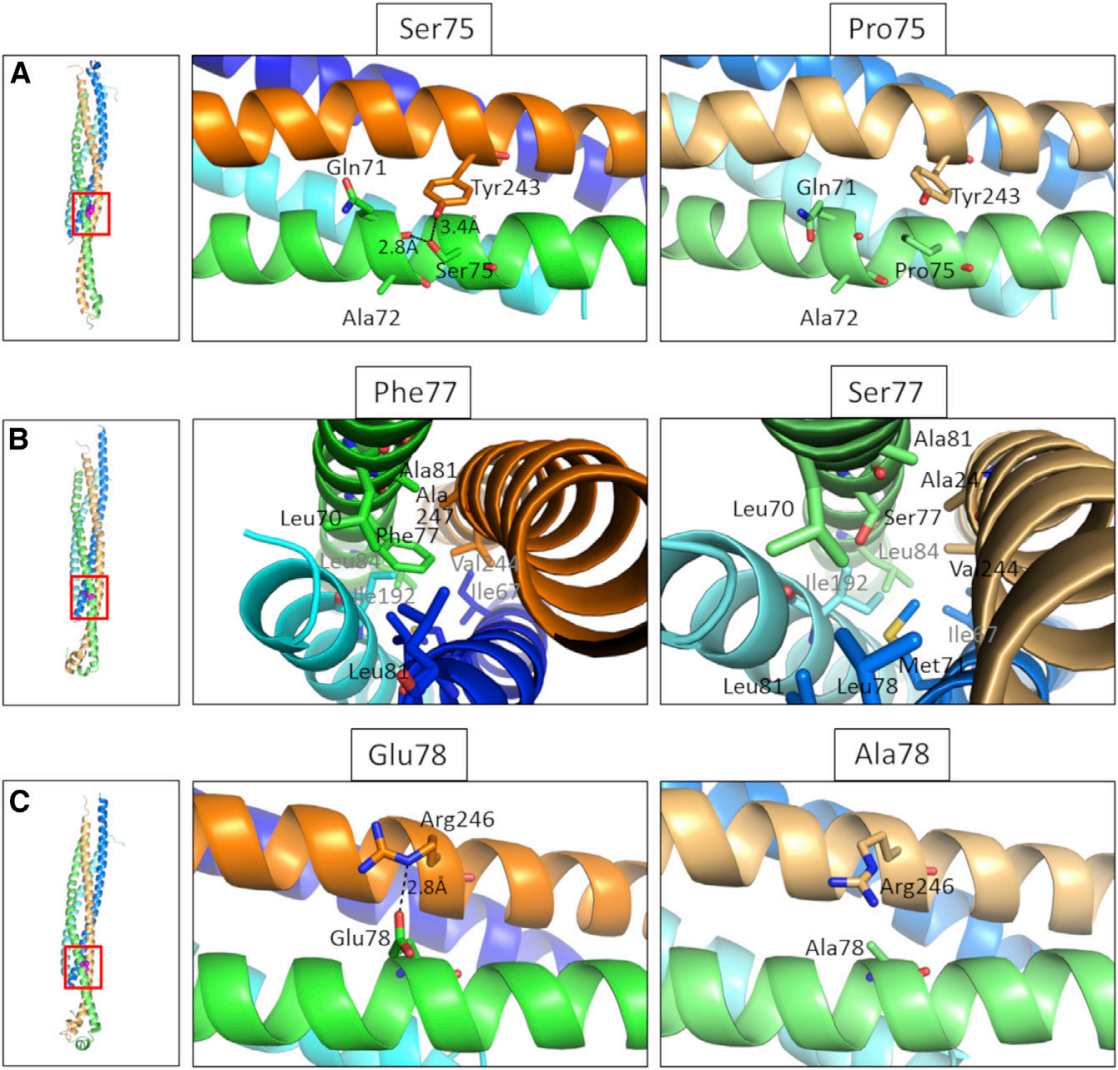
Molecular Modeling of the Identified *De Novo* VAMP2 Non-Synonymous Variants Comparison between the p.Ser75Pro (A), p.Phe77Ser (B), and p.Glu78Ala (C) mutant conformation within the SNARE complex (left panel, red square). The wild-type conformation is shown in the middle panel, and the mutated residues are shown in the right panel. Variant p.Ser75Pro causes the loss of two hydrogen bonds, one interchain between Ser75 of VAMP2 and Tyr243 of STX1A and one intrachain between Ser75 and Gln71; variant p.Phe77Ser introduces a hydrophilic residue in an otherwise hydrophobic region; and variant p.Glu78Ala causes the loss of a hydrogen bond between Glu78 of VAMP2 and Arg246 of STX1A. Modeling of the VAMP2 ectodomain (green for WT, light green for mutants) in complex with STX1A (orange for WT, light orange for mutants) and Snap25 (blue and cyan for WT, marine and aquamarine for mutants); configurations are as seen 100 ns into the molecular dynamic simulation. The complexes were modeled from the humanized 3HD7 complex. Water molecules and ions are not shown.

The *de novo* non-synonymous variants identified in this study cluster in close proximity within the C-terminal portion of the SNARE motif (Figure 2C). Interspecies alignment of protein sequences generated with Clustal Omega show that all mutations occur within the SNARE motif at residues highly conserved through evolution (Figure 2D). Figure 3 shows positions of the mutated amino acids within a 3D structure of the VAMP2 ectodomain in complex with STX1A and SNAP25. Replacement analysis shows that the p.Ser75Pro variant will result in the loss of two hydrogen bonds, one interchain between Ser75 of VAMP2 and Tyr243 of STX1A and one intrachain between Ser75 and Gln71, although the p.Phe77Ser variant introduces a hydrophilic residue in an otherwise hydrophobic region and the p.Glu78Ala variant disrupts the hydrogen bond between Glu78 of VAMP2 and Arg246 of STX1A.

To determine whether these disease-associated variants affect VAMP2 structure and SNARE complex stability, we performed 100 ns molecular dynamics (MD) simulations by using a humanized version of the neuronal SNARE complex (PDB 3HD7, see Supplemental Data). During the simulations, the WT and p.Ser75Pro seemed to reach a stationary state, but major rearrangements were still observed for p.Phe77Ser and p.Glu78Ala at the end of the simulation. This was evident in their backbone root-mean-square deviation (RMSD) and radius of gyration, which measure the divergence of the mutant protein structure from its initial structure over the course of the simulation. In all cases, the most mobile portion of the chain was that close to the C terminus, as seen in their root mean squared fluctuation (RMSF). The RMSF further indicates that in all cases, the variants increase the mobility of the backbone, and this effect is particularly evident for p.Glu78Ala. Overall rearrangements of the complex are shown in Figures S2–S3.

To examine *VAMP2* expression across CNS regions, we used microarray data (Affymetrix Exon 1.0 ST) from human post-mortem brain tissues as previously described.^21^ This analysis showed the highest *VAMP2* expression in the putamen and the frontal lobes (Figure S4).

**Figure 4:**
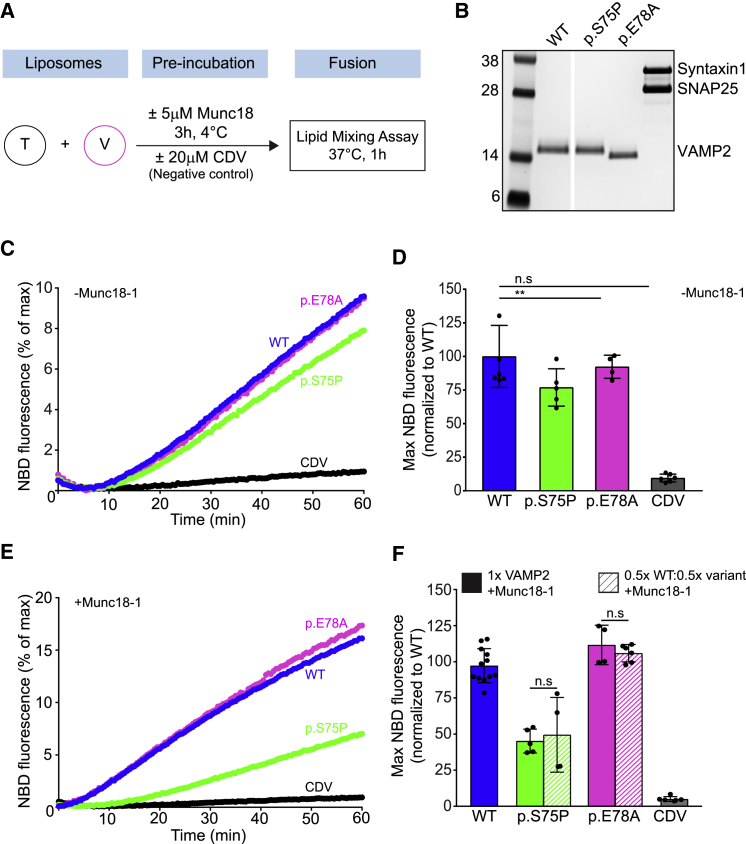
Disease-Associated *VAMP2* Variants Result in Reduced Fusion Rates (A) Scheme showing the liposome fusion assay. (B) The SDS-PAGE and Coomassie-stained gel image of VAMP2 WT, VAMP2 disease-associated variants (p.Ser75Pro [p.Glu78Ala]), and t-SNARE (syntaxin 1 and SNAP25) reconstitution into donor v- and acceptor t-liposomes, respectively. (C) Line graphs showing the average basal (without Munc18-1) increase that occurs in NBD fluorescence as a result of fusion between the v-liposome and t-SNARE liposomes carrying WT or VAMP2 disease variants (p.Ser75Pro [p.Glu78Ala]). Liposome fusion reaction in the presence of CDV was used as negative control. (D) Basal fusion quantification, normalized to WT, at the endpoint (60 min) as described in (C). (E) Line graphs of liposome fusion reaction as in (C), in the presence of 5 μM Munc18-1. (F) Endpoint fusion quantification, normalized to WT, (60 min) of experiment as described in (E). Bar graphs also showed endpoint quantification of a similar experiment that used a v-liposome that contained a mixture of WT and mutant VAMP2 proteins. Data were from at least four independent replicates and presented as means plus SD. ^∗^p < 0.05; ^∗∗^ p < 0.01; ^∗∗∗^ p < 0.001; n.s., not significant (p > 0.05).

To evaluate the functional consequence of *VAMP2* variants, we employed the reconstituted, lipid-mixing assay based on NBD (N-[7-nitro-2-1, 3-benzoxadiazol-4-yl])-to-RHO (lissamine rhodamine B) energy transfer (see Supplemental Data). In this assay, the VAMP2 (wild-type [WT] or mutant) was included in the fluorescent donor liposomes, whereas the t-SNAREs were reconstituted into the non-fluorescent acceptor liposomes. We read out membrane fusion between the donor and acceptor liposome mixing by quantifying increased fluorescence resulting from the dequenching of NBD fluorescence (Figure 4A). To this end, we purified WT VAMP2 and the variant protein along with the t-SNARE complex by using a bacterial expression system as previously described.^22^, ^23^ We were able to purify the p.Ser75Pro and p.Glu78Ala variants, and Coomassie-stained SDS-PAGE analysis showed that these variants were structurally intact and highly pure with no contamination (Figure S5). However, all attempts to isolate the p.Phe77Ser were unsuccessful. We therefore limited our *in vitro* fusion analysis to the two remaining non-synonymous variants (p.Ser75Pro and p.Glu78Ala).

As shown in Figures 4C–4F, the VAMP2 disease-associated variant p.Ser75Pro reduced the rate and extent of fusion compared to that seen with VAMP2 WT, whereas the p.Glu78Ala variant had little to no effect (Figures 4C and 4D). The reduction in the fusion associated with p.Ser75Pro was estimated to be approximately 25% that in the WT, suggesting that the introduction of a proline residue at this site most likely interferes with the proper assembly of the SNARE proteins and thus affects VAMP2 fusion properties, whereas the fusion profile associated with the p.Glu78Ala was indistinguishable from that of the WT.

Earlier studies have shown that Munc18 chaperones SNARE assembly via interactions with the VAMP2 C-terminal region.^12^, ^24^ We therefore investigated the effect of the disease variants under Munc18-activated conditions. As expected, inclusion of Munc18-1 produced an approximately 2-fold increase in the rate and extent of fusion when WT VAMP2 was used (Figure 4E). Strikingly, Munc18 could not activate the fusion mediated by the VAMP2 p.Ser75Pro variant (Figure 4E). Consequently, we observed a significant (>90%) loss-of-function phenotype with the p.Ser75Pro variant under these conditions. In contrast, Munc18 was able to activate the fusion mediated by VAMP2 p.Glu78Ala, confirming that this variant does not affect the SNARE assembly process or its activation. To accurately emulate the physiological make-up of the individuals carrying heterozygous *de novo VAMP2* variants, we also tested the effect of replacing half the copies of WT VAMP2 with the disease variants (Figure S4). Remarkably, in the case of p.Ser75Pro, the fusion profile for the mixed v-liposomes (50:50 WT:mutant) was identical to the fusion profile for the homogenous samples containing only the mutant proteins (Figure 4F; Figure S4). This implies that p.Ser75Pro mutant dominantly interferes with WT (Figure 4F), and this could readily explain the pathological phenotype observed with this variant.

Our genetic and functional studies show that *de novo* mutations in *VAMP2* cause neurodevelopmental impairment associated with variable clinical features. Individuals 1–3, carrying *de novo* non-synonymous variants affecting the C terminus of the VAMP2 SNARE motif (residues 75, 77, and 78), presented a severe neurological phenotype with motor impairment (and inability to walk), central visual deficits, hyperkinetic movements, and, in two of them, epilepsy starting in infancy. Individuals 4 and 5, carrying *de novo* single-amino-acid deletions involving residues at positions 43 and 45, presented a less severe neurological involvement, acquired the ability to walk, and were able to pronounce a few words. MD simulations showed that missense mutations in the C terminus induce higher flexibility of this region within the assembled SNARE complexes. The *in vitro* lipid-mixing assay revealed a significant defect in vesicle fusion as a consequence of the p.Ser75Pro variant, but p.Glu78Ala had no clear functional consequence. The pathophysiological phenotype for the p.Glu78Ala variant might be due to impaired interactions with regulatory proteins that were not included in the *in vitro* assay. Notably, the assembly of the C-terminal region of the SNARE proteins is considered critical to driving membrane fusion,^5^, ^25^ and several synaptic regulatory proteins modulate vesicle fusion by binding the C-terminal portion of the SNARE complex.^12^, ^23^, ^24^ Thus, mutations affecting this region could disturb the SNARE complex assembly by less-efficient partnering of cognate SNARE proteins and/or disrupt its association with regulatory elements such as Munc18-1 or Synaptotagmin. In the physiological context, this would manifest as the perturbation of Ca^2+^-triggered neurotransmitter release. Even a slight alteration of the fusion kinetics *in vitro* would translate to a dramatic effect on the release of neurotransmitters release at the neuronal synapses. This might explain the severe neurodevelopmental impairment observed in the VAMP2 synaptopathy. Interestingly, variants affecting the Ser75 residue have previously been shown to impair the Munc18-1 stimulatory activity by impairing its ability to regulate trans-SNARE zippering,^12^, ^23^ and variants involving residue Glu78 can also affect Ca^2+^-regulated neurotransmitter release.^26^

The present work adds to the evidence that neurodevelopmental disorders (NDDs) have a strong genetic component and encompass a range of frequently co-existing conditions, including ID, developmental delay (DD), and autism spectrum disorders (ASDs).^27^, ^28^ Neurodevelopmental impairment, epilepsy, and movement disorders also frequently co-exist.^29^, ^30^ Rare variants in genes that encode a number of presynaptic proteins involved in Ca^2+^-regulated neurotransmitter release have been identified in individuals affected by a spectrum of neurological disorders. These include the following:

1. variants in *SNAP25* (MIM: 60322) isoforms *SNAP25a* and *SNAP25b;* these variants have been identified in association with ID, seizures, and myasthenia^31^, ^32^
2. variants in *SYT1* (MIM: 185605), which encodes the Ca^2+^-sensor synaptotagmin-1 required for evoked synchronous fusion; these variants are found in individuals with NDDs and hyperkinetic movements^33^, ^34^
3. variants in genes encoding the RIM interactor PNKD or the SNAP25 and synaptotagmin-1 interactor PRRT2; these variants have been identified in different forms of dyskinesias and seizures (MIM: 128200; MIM: 60575)^35^, ^36^
4. variants in *UNC13A* (MIM: 609894), encoding the synaptic regulator Munc13-1; these variants have been linked to an NDD with involuntary movements^37^
5. variants in *STXBP1* (MIM: 602926), encoding Munc18-1; these variants cause NDDs with epilepsy and autistic features^38^

The phenotypes associated with the VAMP2 synaptopathy reported here are reminiscent of the variability reported in some individuals who have *de-novo* variants in *STXBP1* or in *SYT1* and who can present with a combination of neurodevelopmental impairment, stereotypies, hyperkinetic movements (including chorea and dystonia), and EEG anomalies or epileptic syndromes of variable severity.^33^, ^39^

Notably, a heterozygous mutation in a synaptobrevin homolog, *VAMP1*, which encodes a protein involved in vesicle fusion mainly at neuromuscular synapses,^40^ has been linked to spastic ataxia in families from Newfoundland.^41^ More recently, biallelic mutations in *VAMP1* have been identified in association with a phenotype of congenital hypotonia and muscle weakness, and in three of these families neurophysiological evidence of presynaptic neuromuscular transmission impairment was detected and led to a diagnosis of presynaptic congenital myasthenic syndrome.^42^, ^43^, ^44^

In conclusion, we have identified a neurodevelopmental disease that is variably associated with additional neurological features, including epilepsy and hyperkinetic movements, and that is caused by *de novo* mutations in *VAMP2*. These results further delineate an emerging spectrum of human core synaptopathies caused by variants in genes that encode SNAREs and essential regulatory components of the synaptic machinery. The hallmark of these disorders is impaired presynaptic neurotransmission at nerve terminals; this impaired neurotransmission results in a wide array of (often overlapping) clinical features, including neurodevelopmental impairment, weakness, seizures, and abnormal movements. The genetic synaptopathy caused by *VAMP2* mutations highlights the key roles of this gene in human brain development and function. Variability in the effects of different *VAMP2* mutants under *in vitro* conditions points toward mutation-specific mechanisms underlying the presynaptic defect of the affected children, and this variability highlights a promising area of future research.

## Declaration of Interests

The authors declare no competing interests.

## Accession Numbers

The accession numbers for the DNA sequences reported in this paper are in the Leiden Open Variation Database: 00181522, 00181523, 00181524, 00181525, 00181526.

## Web Resources

CADD, https://cadd.gs.washington.edu/
ClustalX, https://www.ebi.ac.uk/Tools/msa/clustalw2/
Ensembl, http://www.ensembl.org/
Exome Aggregation Consortium (ExAC), http://exac.broadinstitute.org
Exome Variant Server, http://evs.gs.washington.edu/
Exome Variant Server of the National Heart, Lung, and Blood Institute Grand Opportunity (NHLBI GO) Exome Sequencing Project (accessed February 2014), http://evs.gs.washington.edu/EVS/
Genome Analysis Toolkit (GATK), https://www.broadinstitute.org/gatk/
GenotypeTissue Expression (GTEx) Project, http://www.gtexportal.org
GnomAD, https://gnomad.broadinstitute.org/
GTEx, http://www.gtexportal.org/home/
Interactive bio-software, https://www.interactive-biosoftware.com/doc/alamut-visual
NCBI ClinVar database, https://www.ncbi.nlm.nih.gov/clinvar/
Online Mendelian Inheritance in Man (OMIM), http://omim.org/
Picard, http://broadinstitute.github.io/picard/
Primer-BLAST, https://www.ncbi.nlm.nih.gov/tools/primer-blast/
UCSC Genome Browser, http://genome.ucsc.edu/
UniProt database, https://www.uniprot.org/

## Supplemental Data

Document S1 Supplemental Recruitment and Diagnosis, Supplemental Case Reports, Figures S1–S6, Table S1, Supplemental Methods, Functional Analyses, Consortia and Networks, and Supplemental References

Document S2. Article plus Supplemental Data
