# Supplementary material for: Mutations in the Neuronal Vesicular SNARE VAMP2 Affect Synaptic Membrane Fusion and Impair Human Neurodevelopment
Source: Am J Hum Genet. 2019 Mar 28;104(4):721–30. doi: 10.1016/j.ajhg.2019.02.016 (PMC6451933; doi:10.1016/j.ajhg.2019.02.016)
Supplement: Document S1 — Supplemental Recruitment and Diagnosis, Supplemental Case Reports, Figures S1–S6, Table S1, Supplemental Methods, Functional Analyses, Consortia and Networks, and Supplemental References [file mmc1.pdf]

The American Journal of Human Genetics, Volume *104*

Supplemental Data

Mutations in the Neuronal Vesicular SNARE *VAMP2* Affect Synaptic Membrane Fusion and Impair Human Neurodevelopment

Vincenzo Salpietro, Nancy T. Malintan, Isabel Llano-Rivas, Christine G. Spaeth, Stephanie Efthymiou, Pasquale Striano, Jana Vandrovcova, Maria C. Cutrupi, Roberto Chimenz, Emanuele David, Gabriella Di Rosa, Anna Marce-Grau, Miquel Raspall-Chaure, Elena Martin-Hernandez, Federico Zara, Carlo Minetti, Deciphering Developmental Disorders Study, SYNAPS Study Group, Oscar D. Bello, Rita De Zorzi, Sara Fortuna, Andrew Dauber, Mariam Alkhawaja, Tipu Sultan, Kshitij Mankad, Antonio Vitobello, Quentin Thomas, Frederic Tran Mau-Them, Laurence Faivre, Francisco Martinez-Azorin, Carlos E. Prada, Alfons Macaya, Dimitri M. Kullmann, James E. Rothman, Shyam S. Krishnakumar, and Henry Houlden

Supplemental Data

Subject recruitment and diagnosis

Case reports

Figures

Tables

Methods

Consortia and network involved in this study

References

Subject recruitment and diagnosis

For each affected individual, clinical data, brain imaging and EEG were reviewed by the clinicians (geneticists, neurologists and child neurologists, paediatricians) from the participating centres. Genomic DNA was extracted from the whole blood or saliva of the affected individuals and their parents after informed consent for DNA analysis was provided by the families. The study was approved by the ethics committee of the University College London (07/Q0512/26) and additional local ethics committees of the participating centres. Parents of the affected individuals (and when available unaffected siblings) were recruited for segregation analysis, which was carried out using Sanger sequencing. The individuals diagnosed with neurodevelopmental impairment including intellectual disability (ID), developmental delay (DD), autism spectrum disorder (ASD), Rett syndrome (RTT)- like stereotypies, and epileptic encephalopathy (EE) were recruited in the different Institutions participating to the study. Based on the International League against epilepsy (ILAE) classification, an EE was defined in the patients as refractory seizures and cognitive slowing or regression associated with frequent, ongoing epileptiform activity.[1]

Based on the RTT diagnostic criteria,[2] the affected individuals from this cohort having at least 1/4 main RTT criteria and ≥4 supportive criteria have been diagnosed with RTT-like features, as previously reported. [3-5]

ID was defined based on the presence of significant deficits conceptual, social and/or practical skills associated to significant deficits in adaptive behavior.[6]

Detailed epilepsy and medical histories were obtained as well as the results of investigations including EEG and MRI studies. The 4 individuals carrying de novo *VAMP2* intragenic variants reported in the present study were recruited from different research groups and consortia in the UK and internationally. Patient 1 was followed up at University Hospital “Gaetano Martino” (Messina, Italy) and was genetically investigated by trio- whole exome sequencing (WES) as part of the SYNAPS Study Group (http://neurogenetics.co.uk/synaptopathies-synaps/) initiative. Patient 2 was recruited and studied by trios WES at Cincinnati Children's Hospital Medical Center (Ohio, USA). Patient 3 was recruited at the Genetic Unit in Cruces University Hospital (Bilbao, Spain) and investigated by trio WES at the Instituto de Investigaciòn Hospital 12 de Octubre (i+12) (Madrid, Spain). Patient 4 was followed up at University Hospital Vall d’Hebron (Barcelona, Spain) and was identified by reviewing the SYNAPS Study Group exome dataset during the replication cohort screening phase of this study. Patient 5 was followed up at University Hospital of Dijon (Bourgogne, France). Routine clinical genetic and metabolic screenings performed during initial workup was negative in each case, which warranted further investigation on a research basis. All families gave written informed consent for inclusion in the study, including for the publication of photographs and videos.

Supplemental case reports

Patient 1

Patient 1 is a 3-year-old Italian girl born at term after an uneventful pregnancy. Familial history was negative for neurological disorders. Her growth parameters, including occipito-frontal circumference (OFC) at birth were within normal range. She has a history of hypotonia and developmental delay since the first month of life (Video S1). She became able to hold her neck at 10 months of age and she acquired the ability of sitting autonomously only at the age of 2 years and 6 months; at the present age of 3 years she still is unable to walk. All other major developmental milestones, were delayed, and her current cognitive and social skills are severely impaired. Since the first months of life, impaired visual fixation was evident, with only occasional and brief episodes of eye contact (Video S2). During the first year of her life, a virtual absence of purposeful hand movements was also noticed (Video S2). At around 6 months of age a complex hyperkinetic movement disorder became evident. This consists in distal choreic-predominant movements (mainly involving upper and lower limbs) associated to dystonic postures of the neck and the trunk. She also exhibit since the first year of life complex motor stereotypies which include arm flapping, RTT-like hand-predominant stereotypies including hand-wringing, repeated clapping, rocking and raspberry blowing. She also present since infancy frequent automatisms (hand to mouth) and frequent episodes of staring (Videos S1-S2). Brain MRI scan performed at the age of 2 years showed some generalised delay in the maturation of myelin, with a reduced volume of the cerebral white matter posteriorly; also, the optic nerves and chiasm were found hypoplastic. Because of the poor visual contact she underwent funduscopic examination which was reported as normal and she was diagnosed with cortical visual impairment. Until the present age of 3 years, she did not presented clinically with epileptic seizures, but ictal EEG recording performed at the age of 15 months showed high- voltage delta activity with interposed sharp wave-slow wave complexes (predominant over the right central and posterior brain regions). She was never put under anti-epileptic drugs (AEDs). She has not showed developmental regression. Currently, she has severe speech impairment (aphasia). Trio WES identified a de novo missense variant in *VAMP2* [NM_014232: c.223T>C (p.Ser75Pro)].

Patient 2

Patient 2 is a 10-year-old Caucasian boy born at term by normal delivery. At birth, his growth parameters were normal. Since the first month of life, he started experiencing multiple episodes per day of focal seizures, with ictal EEG recording showing fast rhythmic activity followed by sharp wave-slow wave complexes over the right occipital areas. Delay in his developmental milestones, as well difficulties with language, social interaction and behavior were noticed since his first infancy. He is non-verbal and does not follow verbal commands. He has severe self-injurious behaviours. He exhibited poor visual fixation since the first months of life and was lately diagnosed with cortical visual impairment (although he can respond to bright colours). During his childhood, frequency and severity of seizures increased with up to 100 episodes per day. At 6 months of age he underwent craniotomy for grid placement and had a right posterior circulation stroke affecting the thalamic and cortical areas. At 18 months of age he had a right temporal lobectomy and subsequent resection of areas of focal cortical dysplasia posterior to the temporal lobe. Around the age of 2 years the Patient developed a hyperkinetic movement disorder with severe chorea associated to dystonic

postures (Video S3). At the age of 6 years he underwent deep brain stimulation which mildly improved the severity of his movement disorder. In addition to neurodevelopmental impairment, epilepsy and abnormal movements, he presented complex motor stereotypies, including hand stereotypies (e.g., wringing), rocking, oral automatisms (hand to mouth), and virtual absence of purposeful hand movements (Video S3). He also present behaviour abnormalities with episodes of self-injuries, head banging, screaming spells, and exaggerated startle response. Because of his behaviour, he was put under risperidone and escitalopram and paliperidone. At his current age of 10 years, he present motor impairment (he can sit independently but he has backward falling and cannot stand nor walk without assistance), and his seizures are now significantly decreased in frequency and severity with only 1-2 episodes per month. Brain MRI, microarray and metabolic studies were performed and resulted as normal. He performed array-CGH that was normal. Trio-WES identified a variant in *VAMP2* [NM_014232: c.233>C (p.Glu78Ala)] as the only rare *de-novo* variant in the child.

Patient 3

He is a 13-year-old Spanish boy born at term by normal delivery. At birth, his growth parameters including OFC were normal. At 20 days of life episodes of apneas were detected, with the exclusion of infectious, metabolic or cardiologic causes. At the age of 5 months the Patient was evaluated because of a delay in his developmental milestones. At that time, the child was not yet able to hold his neck; in addition, axial hypotonia, poor visual fixation and stereotyped hand movements (e.g., clapping, washing) were noticed. He presented a severe delay in motor milestones and never attained the ability to walk (and became wheelchair-dependent). The Patient exhibited since the first months of his life poor visual fixation since the first months of life and was lately diagnosed with cortical visual impairment. During his infancy he developed a hyperkinetic movement disorder characterized by the combination of choreic movements (predominant in the upper limbs). At the age of 12 months he presented with episodes consisting in brief lapse of consciousness with staring and upward rolling of the eyes; these episodes were associated to generalized paroxysm and lentified below activity on EEG. He was put under valproic acid and then vigabatrine, but episodes changed becoming generalized spasms with disorganization and paroxysms on EEG. At the present age of 13 years he is under clonazepam treatment and chetogenic diet and does not present anymore epileptic episodes. He still present flapping, stereotyped hand movements, and choreic hyperkinetic movements (Video S4). He is non-verbal and present difficulties in social interaction. He underwent multiple metabolic and genetic test including array CGH and Sanger sequencing of different genes (*MECP2, CDKL5, FOXG1*) which resulted normal. Trio-WES identified a variant in *VAMP2* [NM_014232: c.230T>C (p.Phe77Ser)] that was confirmed *de-novo* by Sanger sequencing.

Patient 4

He is a 14-year-old Caucasian boy who was born at term after an uneventful pregnancy and a normal delivery. There is no history of neurological disorders in the family. Both parents and 2 younger sibs are healthy. Global developmental delay was already suspected at 6 months of age. Further milestone achievements confirmed the patient's neurodevelopmental disorder including mild hypotonia, gross motor delay (he attained independent ambulation after 3 age years) and some degrees of clumsiness and incoordination (Video S5). Severe receptive and expressive language disorder was also evident since infancy and, at his present age of 14 years, he is able to understand simple verbal commands and use gestural expressive communication and a 5- 10 simple words. His visual contact and fixation were present since early infancy, but he presented impaired social communication, abnormal behaviour with restricted interests and prominent hand stereotypies (Video S5) including Rett syndrome (RTT) – like features (e.g., hand wringing, washing, clapping) that

led to the diagnosis of Autistic Spectrum Disorder (ASD). Currently, clinical examination does not show definite dysmorphic features; there have not been signs of developmental regression and OFC is normal. No motor or sensory anomalies are apparent, and he is able to walk without assistance. Brain MRI, visual evoked potentials, metabolic screening, Multiplex Ligation-dependent Probe Amplification (MLPA) for sub telomeric deletions, array-CGH and panel sequencing of 55 genes linked to non-syndromic intellectual disability were all normal. He developed infrequent staring episodes with eyelid myoclonus at 5 years of age. Several EEG recordings showed both generalized and multifocal interictal epileptiform discharges and sodium valproate was started. He remained seizure-free until the age of 11 years when he suffered a single episode consistent with non-convulsive status epilepticus, described as with impaired consciousness and subtle clonic movements involving the right arm during which the patient could maintain stance or even walk around. Valproic acid dose was adjusted and he has remained seizure free ever since and with normal follow-up EEGs. WES identified a de novo single amino acid deletion at position 43 [NM_014232: c.128_130delTGG (p.Val43del)].

Patient 5

Patient 5 is a 3-year-old French girl born at term after an uneventful pregnancy. Familial history was negative for neurological or genetic disorders. Her growth parameters, including OFC at birth were within normal range. She has a history of hypotonia since birth and developmental delay was evident since the first month of life. She became able to hold her neck at the age of 9 months and to sit with support at the age of 12 months. All other major developmental milestones were delayed, and her current cognitive and social skills are severely impaired. His visual contact and fixation were present since early infancy, but she presented mild convergent strabismus (right > left), hyperopia and astigmatism. She has impaired verbal and non-verbal communication, abnormal behaviour and stereotypies including arms flapping and hand washing and clapping, that led to the diagnosis of Autistic Spectrum Disorder (ASD). She never presented seizures and her EEG was unrevealing. Currently, clinical examination does not show definite dysmorphic features. She has a wide nasal bridge, anteverted nares, thick lips, full cheeks, stellar iris, and excessive skin in the neck. At the present age of 3 years, she is able to pronounce 4-5 words and became capable of walking when parents give her one hand. She currently benefits from speech therapy, psychomotricity and physiotherapy. She has a normal height and weight growth. Brain MRI, visual evoked potentials, metabolic screening, Multiplex Ligation-dependent Probe Amplification (MLPA) for sub-telomeric deletions and array-CGH were all normal. WES identified a de novo single amino acid deletion at position 45 [NM_014232: c.135_137delCAT (p.Ile45del)].

Supplemental Figures

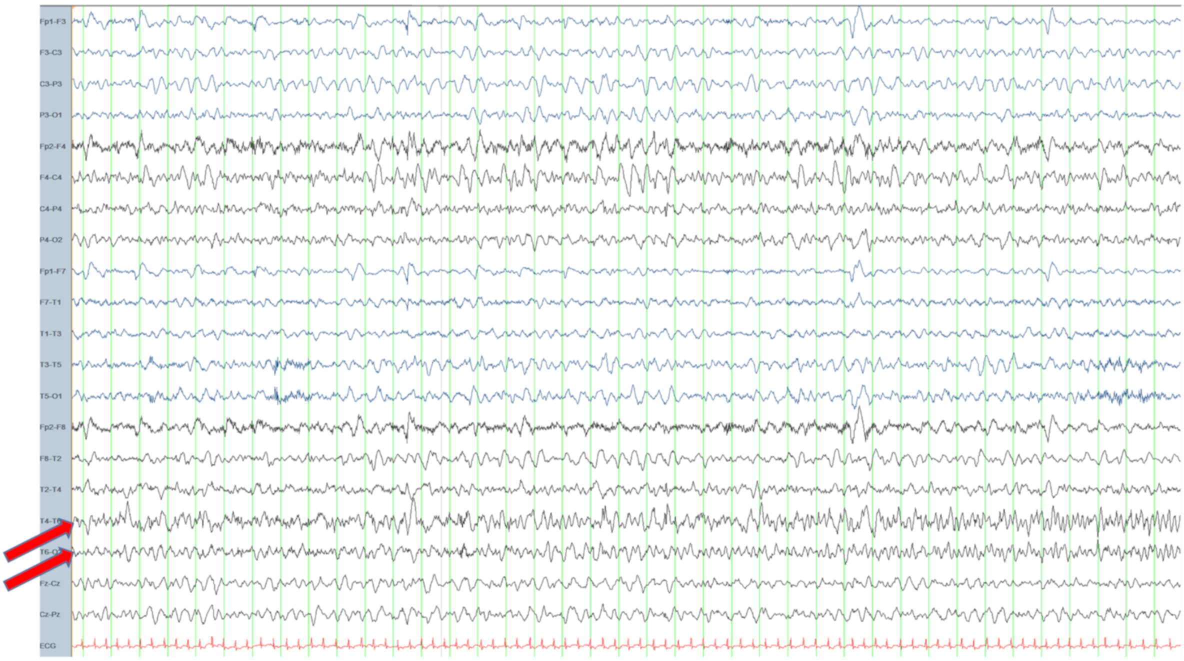

Supplementary Figure 1. Ictal EEG showing a focal seizure.

Ictal EEG recording from Patient 2 (E78A) at the age of 8 months showing a focal seizure characterized by fast rhythmic activity intermixed with high-voltage polymorphic theta activity and sharp waves over the right anterior temporal brain areas. The EKG trace shows concomitant sinusal tachycardia.

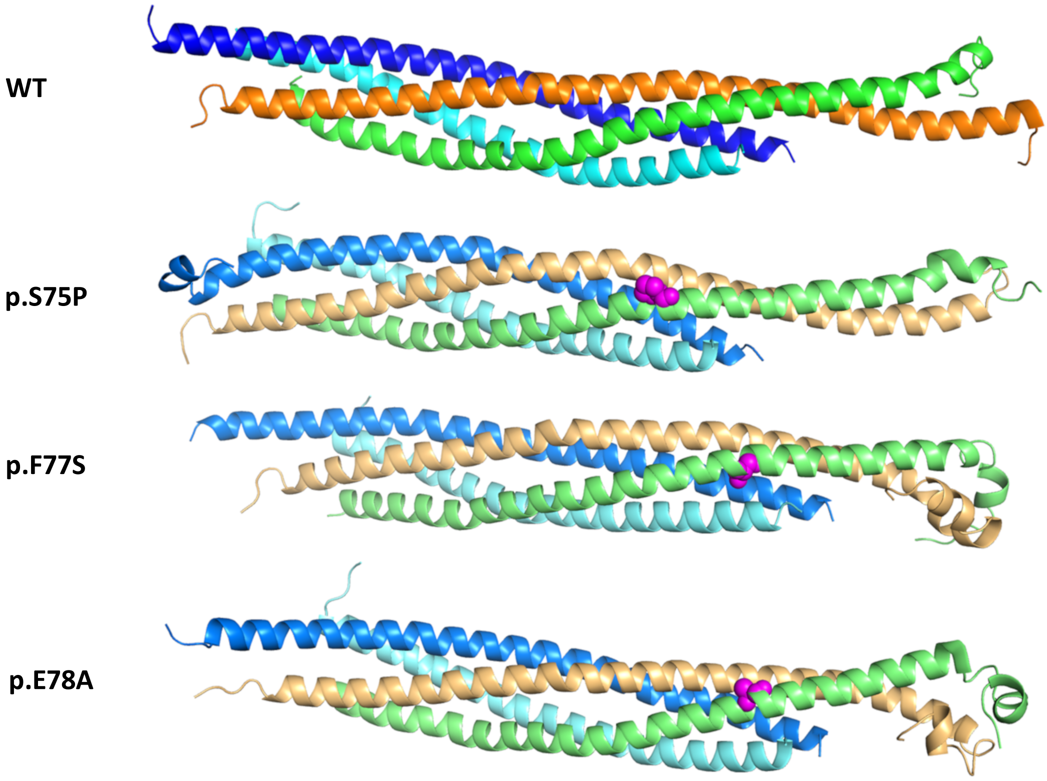

Supplementary Figure 2. Molecular Modelling of identified *VAMP2* variants.

Rearrangement of the VAMP2 ectodomain/Stx1a/Snap25 complex after 100 ns of molecular dynamics simulation for the wild type VAMP 2 protein and its mutants. VAMP2 ectodomain is depicted in green for Wild Type, light green for mutants; Syntaxin-1A is depicted in orange for Wild Type, light orange for mutants; Synaptosomal-associated protein 25 chains are represented in blue and cyan for Wild Type, marine and aquamarine for mutants. Point of mutation is represented with magenta spheres.

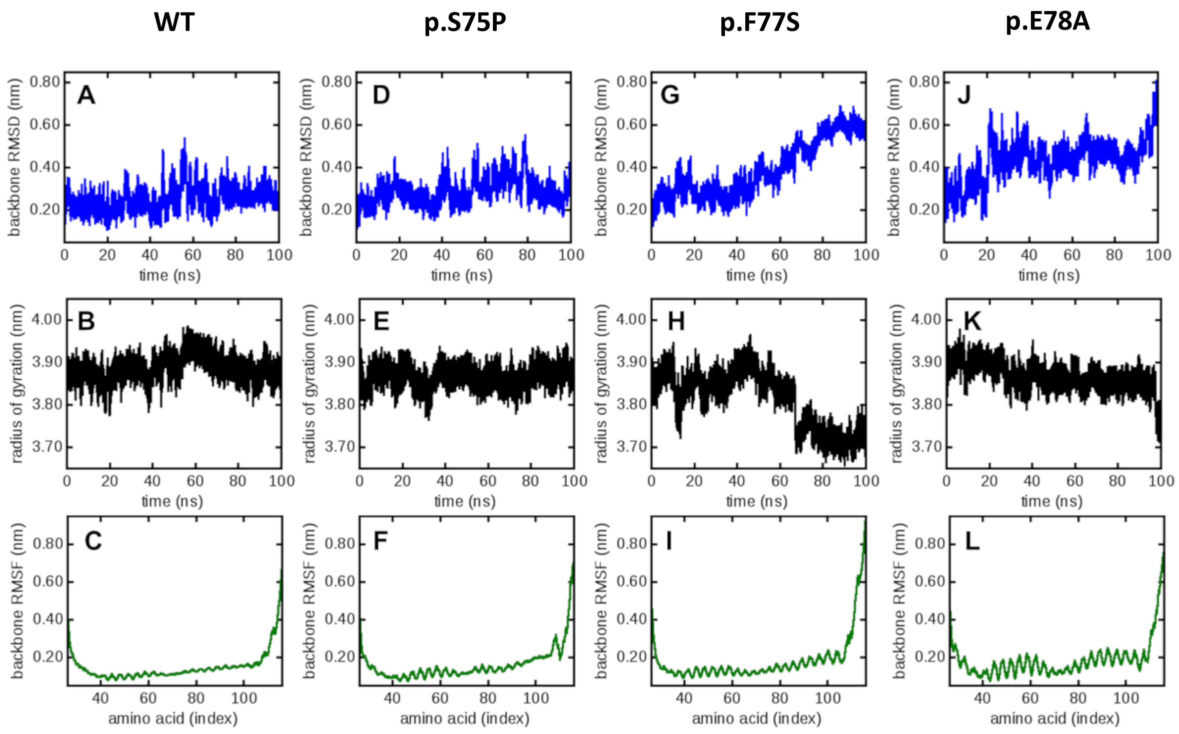

Supplementary Figure 3. Backbone Root Mean Square Deviation of identified *VAMP2* variants.

Backbone Root Mean Square Deviation (RMSD, blue), backbone radius of gyration (black), and backbone RMSF (green) for (A-C) VAMP2 wilde type and its mutants (D-F) S75P, (G-I) F77S, and (J-L) E78A.

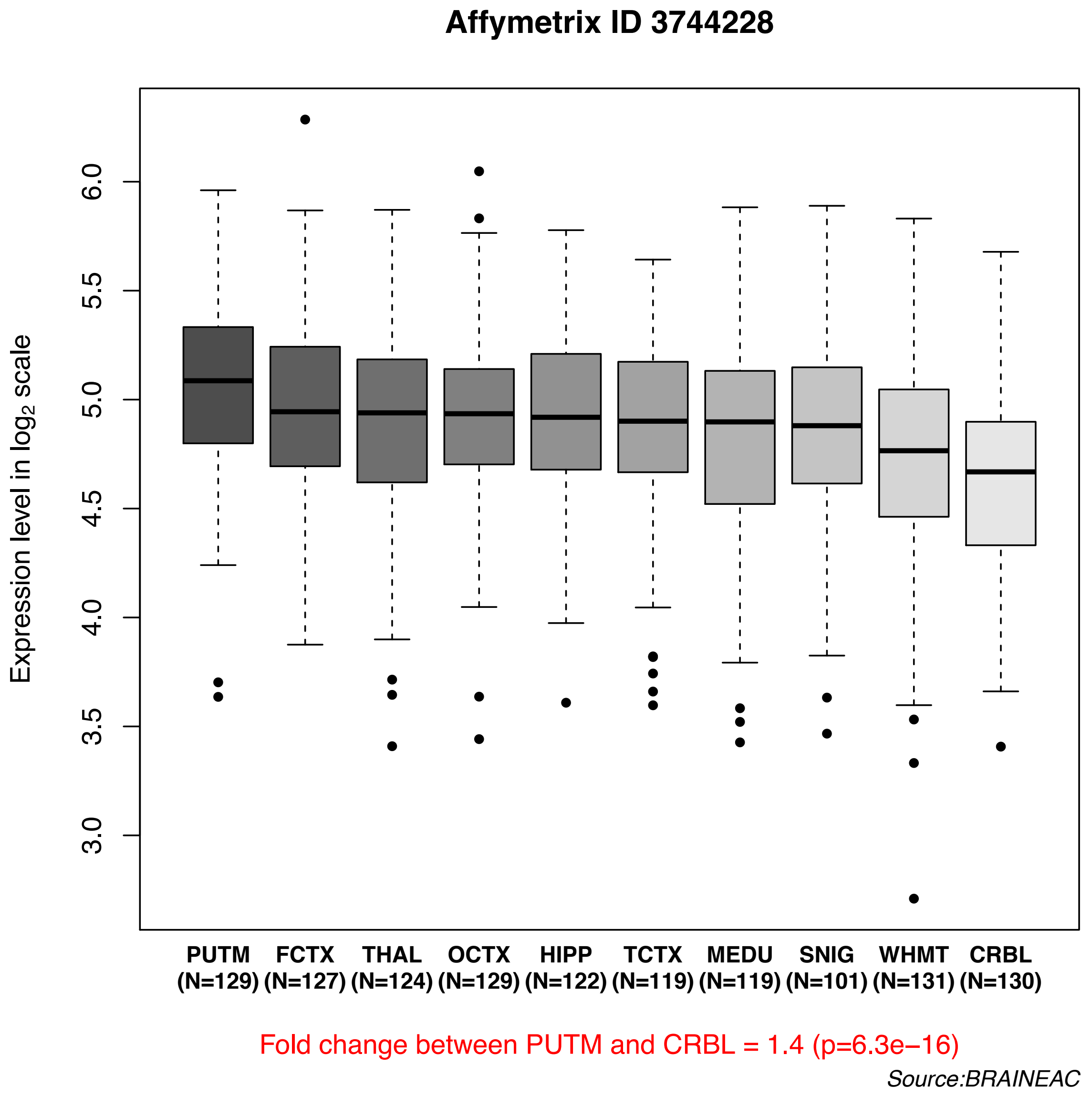

Supplementary Figure 4. Regional brain expression of *VAMP2*

Brain expression values of *VAMP2* show higher expression levels in the striatum and also other CNS regions, especially FCTX (frontal cortex), THAL (thalamus) and OCTX (occipital cortex), and less in TCTX (temporal cortex), HIPP (hippocampus), WHMT (white matter), SNIG (substantia nigra), MEDU (medulla), and CRBL (cerebellar cortex).

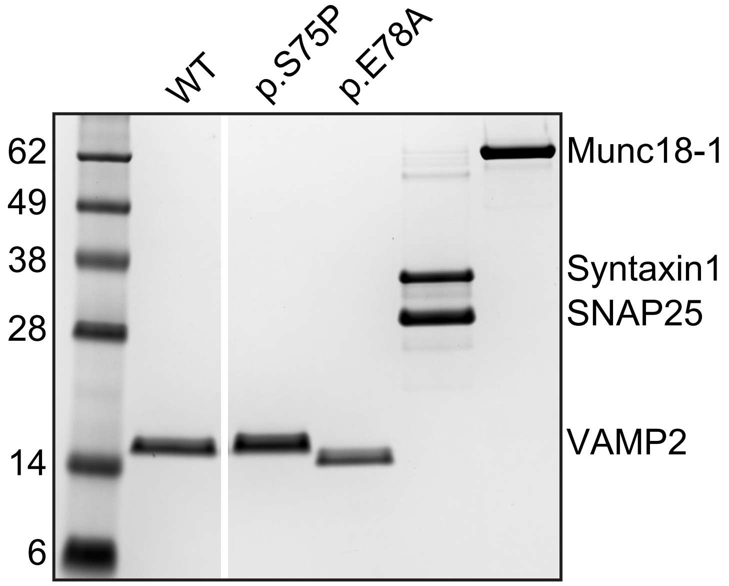

Supplementary Figure 5. Disease variants VAMP2 proteins expression and purification.

The SDS-PAGE and Coomassie-stained gel image showing the disease variants VAMP2 proteins (A67P, S75P and E78A) expressed and purified to similar integrity to the WT protein. The t-SNARE (Syntaxin1 and SNAP25) and Munc18-1 proteins used in the experiments were also analysed on gel. All proteins were resolved to their corresponding molecular size, indicating reasonable protein quality.

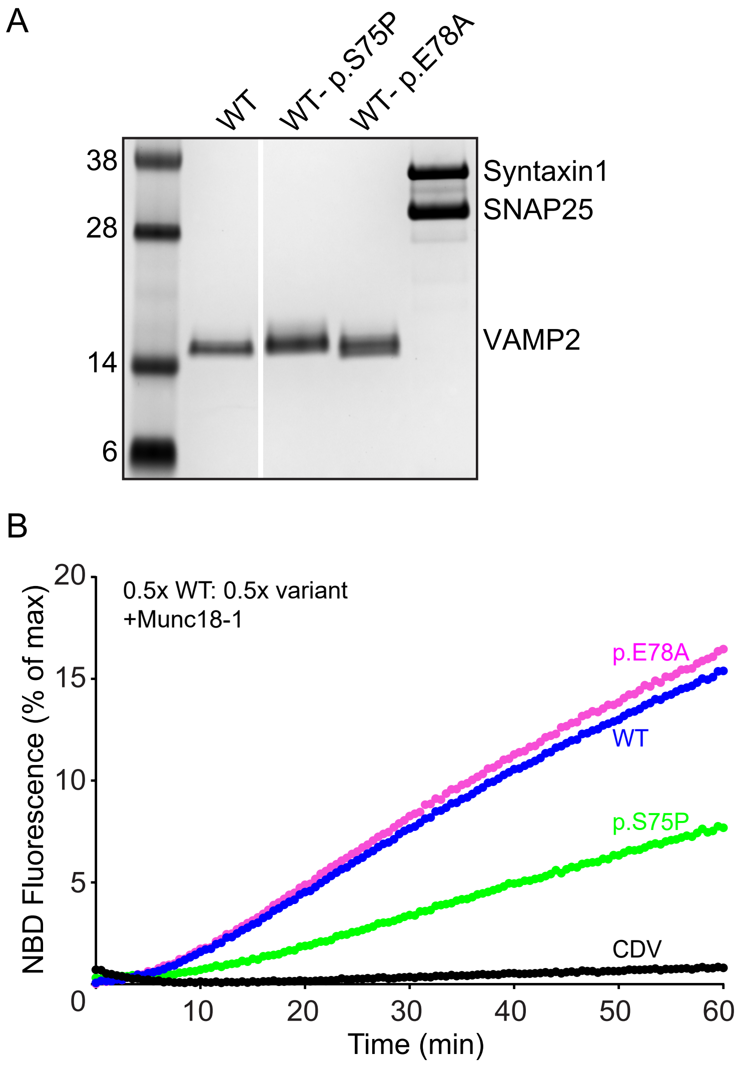

Supplementary Figure 6. Disease variants VAMP2 proteins cause loss-of-function in SNARE-mediated liposome fusion assay.

(A) The SDS-PAGE and Coomassie-stained gel image showing donor v-liposomes reconstituted with VAMP2 wildtype (WT), and mixture of WT and disease variants VAMP2 (A67P, S75P, E78A). Gel also shows t-SNARE (Syntaxin1 and SNAP25) acceptor t-liposomes. (B), Line graphs showing the average (with Munc18-1) increase in NBD fluorescence due to fusion between WT or WT-disease variants VAMP2 (A67P, S75P, E78A) v- and t-SNARE liposomes. Liposome fusion reaction in the presence of CDV was used as negative control.

Supplemental Table 1. De novo *VAMP2* variants identified in this study

Patient	Genomic coordinates (GRCh37/hg19)	Variant	dbSNP138	1000G	ESP6500	ExAC	gnomAD	SIFT	PolyPhen	Mutation Taster	CADD_PHRED	$GERP^{++}$
1	17:8064985-8064985	NM_014232: c.223T>C; p.Ser75Pro	-	-	-	-	-	D(0)	D(0.925)	D(1)	27.3	5.26
2	17:8064978-8064978	NM_014232: c.230T>C; p.Phe77Ser	-	-	-	-	-	D(0)	D(0.997)	D(1)	27.7	5.26
3	17:8064975-8064975	NM_014232: c.233A>C; p.Glu78Ala	-	-	-	-	-	D(0)	D(0.56)	D(1)	25.9	5.26
4	17:8065077-8065080	NM_014232: c.128_130delTG G ; p.Val43del	-	-	-	-	-	-	-	-	-	-
5	17:8065071-8065073	NM_014232: c.135_137delCAT; p.Ile45del	-	-	-	-	-	-	-	-	-	-

Supplemental Methods

Genetic analyses

All research centres involved in this study followed a trio-based WES approach to identify the de novo *VAMP2* variants as the cause of the neurodevelopmental phenotypes of the patients. Most of the methods used by the centres were detailed previously. The SYNAPS Study Group (neurogenetics.co.uk/synaptopathies-synaps) analysed approximately 335 trios of children with neurodevelopmental impairment (as part of a larger cohort of ~4,750 individuals affected with early-onset neurological disorders). Following their respective analysis pipelines, participating centres generated a list of candidate variants filtered against public database variants and according to modes of inheritance. All variants reported in the present study were determined independently by participating centres. Connecting the different contributing centres was facilitated by the web-based tools.[7]

WES data analysis and variant calling

Libraries were prepared from parent and patient DNA, and exomes were captured and sequenced by trio-WES on Illumina sequencers. Raw data were processed and filtered with established pipelines at the academic laboratories involved in the study.[8-12] In Patient 2, 3 and 5, the sequence reads were aligned against the human reference genome (GRCh37/hg19) using Burrows-Wheeler Aligner (BWA) in order to obtain candidate variants. Single-nucleotide variants (SNVs) and short insertion or deletion variants (indels) were identified using Haplotype Caller of GATK (v3.3.0) according to the Best Practices for variant analysis. In Patients 1 and 4, variant (single nucleotide variants and indels) calling and filtering was performed using the Genome Analysis Tool Kit (GATK; see URLs). In all cases, variants that did not adhere to the following criteria were excluded from further analysis: (1) allele balance of >0.70, (2) QUAL of <20, (3) QD of <5 and (4) coverage of <20×.

Variants were annotated and the Exome variant server ESP6500 (evs.gs.washington.edu) was used to assess variant frequency in the control population. In the index case trio WES (Patient 1) the average sequencing depth of the on-target regions was 78.2 reads per nucleotide, with 96.8% of the regions covered at least 20X.

In all five trios studied, only exonic and donor/acceptor splicing variants were considered. Synonymous variants were also excluded. Priority was given to rare variants (<1% in public databases, including 1000 Genomes Project, NHLBI Exome Variant Server, Complete Genomics 69, and ExAC with a GERP++ score > 2). PCR and Sanger sequencing were conducted according to standard methods (detailed conditions of the primers used, and sequencing methods are available upon request). The filtered variants were confirmed by the conventional Sanger sequencing according to standard methods (available upon request).

Variants filtering and identification of *VAMP2* variants

Following their respective analysis pipelines, participating centres generated a list of candidate variants filtered against public database variants and according to modes of inheritance. All participating centres prioritized autosomal recessive and dominant de novo mutations in the analysis and annotated variants using the Variant Effect Predictor (Ensembl release 75) based on Sequence Ontology nomenclature: missense variant, initiator codon variant, splice donor or acceptor variant, frameshift variant, stop lost, stop gained, in frame insertion or deletion. To exclude likely benign amino acid changes, missense variants were further considered only if predicted damaging by at least 3 out of 5 in-silico methods we used (PolyPhen-2, SIFT, Mutation Taster, Condel and CADD, see URLs). Variants that were not present in both the mother and the father of the probands were considered de novo. In recessive filtering, homozygous, hemizygous or compound heterozygous variants were included. Variants present in >1% of our internal exome dataset at the UCL Institute of Neurology (containing ~ 5000 exomes from individuals affected with a range of neurological disorders) were excluded. Exome data were analyzed for variants in genes linked before to NDDs, epilepsy and RTT-like presentations, and for variants in possible new genes. Genes involved in EE and RTT-like presentations were retrieved from the literature.[4,5] Based on values from the ExAC database (containing 60,706 individuals), we also prioritized variants in genes with high probability of being LoF intolerant (i.e., ExAC pLI >0.9) and highly constrained for missense variations (Z-score >2). In our analysis, we also prioritized variants affecting domains important to genes function (UniProt database) and variants in genes implicated in brain development and function (literature) and variants in genes predominantly expressed in the central nervous system (GTEx database). In the case of new candidates, variants in genes whose homologues or functionally correlated genes are already established causing similar neurological disorders were also prioritized. In Patient 1 who was analyzed in the discovery phase of this study, the only de novo variant identified by WES and confirmed by Sanger sequencing was in *VAMP2* [NM_014232: c.223T>C (p.Ser75Pro)]. Similarly to the index case (Patient 1), also in other research and diagnostic laboratories the identified de novo variants in *VAMP2* [NM_014232: c.230T>C (p.Phe77Ser), c.233A>C (p.Glu78Ala), c.128_130delTGG (p.Val43del), c.135_137delCAT (p.Ile45del)] were prioritized (Supplemental Table 1).

VAMP2 mutations thus emerged in each participating centre as the most likely explanation for the individuals disease pathogenesis, as supported by crucial role of the gene in synaptic transmission and the high conservation and biological importance of the affected residues within the C- terminus of the v-SNARE domain. Variants were submitted to Leiden Open (source) Variation Database (LOVD; www.lovd.nl). All the de novo *VAMP2* variants were found with trio WES and confirmed in all cases by trio Sanger sequencing. All variants in were annotated with the transcript NM_014232. For trio Sanger, the following sets of intronic primers designed by Primer3 were used for both PCR and sequencing: Forward (5’- CTGTGTGTCCTTGGCATGTT- 3’) Reverse (5’-ATACCCCATTCACCCACCTG -3’). PCR products were amplified using 50 ng of DNA, with standard FastStart PCR reagents (Roche), on an ABI Veriti Thermal Cycler (Applied Biosystems). PCR products were purified using Exo-SAP (Exonuclease I and Shrimp Alkaline Phosphatase; incubated at 37°C for 15 minutes followed by inactivation by heating to 80°C for 15 minutes) and sequencing PCR was performed bi-directionally using BigDye Terminator Ready Reaction Mix kit version 3.1 (Applied Biosystems) and analysed on an ABI 3730xl capillary sequencer.

Replication cohort screening

As part of our replication cohort analysis we then searched for variants in the *VAMP2* gene within genetic datasets from undiagnosed patients recruited in the SYNAPS Study Group which contains approximately 335 trios of children with neurodevelopmental impairment as part of a larger cohort of ~4,750 individuals affected with early-onset neurological disorders. This led to the identification of Patient 4 carrying a de novo single amino acid deletion at position 43 [NM_014232: c.128_130delTGG (p.Val43del)]. Another de novo single amino acid deletion at position 45 [NM_014232: c.135_137delCAT (p.Ile45del)] was identified by comparing results with international colleagues through the Web-based tool GeneMatcher.[13-15]

Functional analyses

Recombinant proteins expression and purification

All recombinant protein constructs used in the experiments were expressed in bacteria BL21 (DE3) E. coli cell lines. The pTW34-trans-SNARE construct co-encoding the 6-histidine-tagged mouse SNAP25 and rat Syntaxin1a (pTW34-t-SNARE) was expressed and purified as previously described.[16, 17] Upon overnight affinity pulldown on the Ni-NTA beads at 4 °C, t-SNARE proteins were eluted into an n-Octyl-D-glucopyranoside (OG) buffer A (25 mM HEPES-OH pH7.4, 400 mM KCl, 10 % glycerol, 1 % OG and 1 mM DTT) containing 300 mM imidazole pH7.4. The pET-6-histidines-SUMO-tagged constructs encoding the rat Munc18-1, full-length wildtype (WT) mouse VAMP2 and cytoplasmic domain of VAMP2 (CDV) were described.[18] Site-directed mutagenesis using the WT VAMP2 as template was performed to generate the disease variants VAMP2 containing amino acid substitution at Serine75 to Proline (S75P) and Glutame78 to Alanine (E78A). The WT and variants VAMP2 proteins were then expressed and purified as described previously (Shen J et al; 2007). The His6-SUMO proteins were incubated for 3-hour at 4 °C on the Ni-NTA affinity beads, followed by washing using OG buffer for VAMP2 and buffer A for Munc18-1. The SUMO-tag was removed by overnight cleaving on beads using 50 ul of SUMO-protease at 4 °C. The concentration of SUMO-protease used for tag-cleaving is typically at 4-7 mg/ml. The VAMP2 proteins were eluted into OG buffer and Munc18-1 was eluted into buffer A without OG. Purified proteins were evaluated on SDS-PAGE and Coomassie-stained. Protein concentrations were determined by colourimetry reaction using Bradford dye.[19]

Proteoliposome reconstitution for lipid mixing assay

The purified SNARE proteins were reconstituted into lipid vesicles (Avanti Polar Lipids) by detergent dilution and dialysis. Proteoliposomes were isolated by floatation using Nycodenz density gradient as previously described.[17] The t-SNARE protein was reconstituted into palmitoyl-2-oleoyl phosphatidylcholine (POPC): 1,2 dioleoyl phosphatidylserine (DOPS) at 85:15 mol% liposomes at protein to lipid ratio of 1:400. The VAMP2 proteins used in the reconstitution were into POPC, DOPS and the fluorescent probes NBD-PE and rhodamine- PE (1.5 mol% each) at protein to lipid ratio of 1:150, mimicking the reported VAMP2 densities on the synaptic vesicles.[20, 21] Proteoliposomes containing mixture of WT VAMP2 and mutant proteins were reconstituted at protein to lipid ratio of 1:300 each.

Liposome fusion assay

The lipid-mixing assay utilised the Föster resonance energy transfer (FRET) principle.[22, 23] In this assay, v- SNARE liposomes contained PE-lipids labelled with fluorophores Nitro-2-1, 3 benzoxadiazol-4yl- phosphatidylethanolamine (NBD-PE) and lissamine rhodamine B (RHO). Within a closed distance in a liposome, NBD fluorescence is quenched in the presence of rhodamine. Upon fusion of v-SNARE liposomes with the unlabeled t-SNARE liposomes, the spatial interaction between NBD and rhodamine molecules increased. Dequenching of NBD, concomitant with increase in NBD fluorescence provide a measure of liposome fusion.[22] Liposome fusion assay was performed by mixing 5 µl of the donor liposome (VAMP2) with 45 µl of the acceptor liposome (t-SNAREs). Liposomes mixes were pre-incubated in the presence or absence of Munc18-1 on ice for 3-hour to allow for trans-SNARE complex assembly. Fusion of liposomes was monitored by the change in NBD

fluorescence at 538 nm using a fluorescent microplate reader. After 60 min, 10 µl of 2.5 % w/v n-dodecyl-β-maltoside was added to lyse all vesicles to estimate the maximum NBD fluorescence.[13]

Molecular modelling and dynamic stimulations

We build a model of each mutant ectodomain by homology modelling with the humanised wild type (WT) as a template. We follow the behaviour of the WT and each mutant along time by means of full atom molecular dynamics simulations in water solvent. The soluble WT VAMP2 fragment was built from the first complex of the asymmetric units of structure PDB ID 3HD7 representing the neuronal SNARE complex from Rat.[24] We humanized the complex by mutating two residues (V278I, V283I) in Chain B (protein Stx1a). The reported VAMP2 chain (Chain A) does not include the mutation (V8A) from Rat to Human and therefore it was left as it is. The same applied to the other two chains (chain C e D, SNAP25). The humanised complex was then placed in a cubic box, and minimized. All the other mutants were constructed from this minimised humanised complex. All the complexes containing the mutants, including the WT, were then placed in a triclinic box and minimised. A water layer of 0.8 nm and Na^+ ions to neutralize the system were added, and a second minimization was performed. In all cases we used AMBER99SB-ILDN force field and Simple Point Charge water. On all systems we performed NVP and NPT equilibrations for 100 ps, followed by 100 ns NPT production run at 300 K. The temperature was controlled with a modified Berendsen thermostat[25] the pressure with an isotropic Parrinello-Rahman at 1 bar. The iteration time step was set to 2 fs with the Verlet integrator and LINCS constraint.[26] We used periodic boundary conditions. Configurations were sampled every 10ps. All the simulations and their analysis were run as implemented in the GROMACS.[27] During the simulations, the WT and S75P seem to reach a stationary state while major rearrangements are still observed for F77S and E78A at end-simulation as emerges in their backbone root mean squared deviation (RMSD, Supplementary Figure 3 A, D, G, J) and radius of gyration (RMSD, Supplementary Figure 3B, E, H, K). In all cases the most mobile portion of the chain is that close to the C-term as seen in their root mean squared fluctuation (RMSF, Supplementary Figure 3 C, F, I, L). The RMSF further indicates that the in all cases the mutations enhance the mobility of the backbone, an effect particularly evident for E78A (Supplementary Figure 3L).

Consortia and networks involved in this study

The Deciphering Developmental Disorders (DDD) Study (http://www.ddduk.org/)

Central DDD Team

Wellcome Trust Sanger Institute, Wellcome Trust Genome Campus, Hinxton, Cambridge, CB10 1SA, UK & The Ethox Centre, Nuffield Department of Population Health, University of Oxford, Old Road Campus, Oxford, OX3 7LF, UK

DDD Management Team (*Principal Investigator)

Jeffrey C. Barrett, Nigel P. Carter, Helen V. Firth, David R. Fitzpatrick, Matthew E. Hurles*, Michael Parker, Caroline F. Wright

DDD Laboratory Team

Kirsty Ambridge, Daniel M. Barrett, Tanya Bayzetinova, Susan Gribble, Netravathi Krishnappa, Laura E. Mason, Elena Prigmore, Diana Rajan

DDD Model Organisms

Eve L. Coomber, Sebastian S. Gerety

DDD Informatics Team

Stephen Clayton, Tomas W. Fitzgerald, Philip Jones, Ray Miller, Adrian R. Tivey DDD Analysis Team Nadia Akawi, Saeed Al-Turki, Jeffrey C. Barrett, Tomas W. Fitzgerald, Matthew E. Hurles, Wendy D. Jones, Daniel King, Margriet van Kogelenberg, Jeremy McRae, Katherine I. Morley, Vijaya Parthiban, Alejandro Sifrim

DDD Ethics, Social Science and Policy Team

Anna Middleton, Michael Parker, Caroline F. Wright

Wellcome Trust Sanger Institute Staff

Wellcome Trust Sanger Institute, Wellcome Trust Genome Campus, Hinxton, Cambridge, CB10 1SA, UK

DECIPHER Team:Paul Bevan, Eugene Bragin, G. Jawahar Swaminathan

WTSI Pipelines Staff (sample QC, genotyping, pulldown, sequencing, informatics)

Rob Andrews, John Burton, Suzannah J. Bumpstead, Sarah Edkins, Peter Ellis, Emma Gray, David Jones, Carol Scott, Douglas Simpkin, Danielle Walker, Sara Widaa

WTSI FISH Team

Ruby Banerjee, Beiyuan Fu, Sandra Louzada Gomes Pereira, Fentang Yang

UK NHS Regional Genetic Services (* local Principal Investigator)

Aberdeen (North of Scotland Regional Genetics Service, NHS Grampian, Department of Medical Genetics Medical School, Foresterhill, Aberdeen, AB25 2ZD, UK)

Recruiting Consultant Clinical Geneticists: John Dean*, Ruth McGowan, Alison Ross

Research Nurse/ Genetic Counsellors: Mariella D'Alessandro Diagnostic Laboratory Scientists: Paul Batstone, Shalaka Samant

Belfast (Northern Ireland Regional Genetics Centre, Belfast Health and Social Care Trust, Belfast City Hospital, Lisburn Road, Belfast, BT9 7AB, UK)

Recruiting Consultant Clinical Geneticists: Tabib Dabir, Deirdre Donnelly, Alex Magee, Vivienne McConnell, Shane McKee*, Fiona Stewart

Research Nurse/ Genetic Counsellors: Claire Kirk Diagnostic Laboratory Scientists: Mervyn Humphreys, Susan McNerlan

Birmingham (West Midlands Regional Genetics Service, Birmingham Women's NHS Foundation Trust, Birmingham Women's Hospital, Edgbaston, Birmingham, B15 2TG, UK)

Recruiting Consultant Clinical Geneticists: Louise Brueton, Trevor Cole*, Nicola Cooper, Helen Cox, Joanna Jarvis, Derek Lim, Jenny Morton, Andrew Norman, Chirag Patel, Nicola Ragge, Saba Sharif, Mark Tein, Julie Vogt, Denise Williams

Research Nurse/ Genetic Counsellors: Gail Kirby

Diagnostic Laboratory Scientists: David Bohanna, Kirsten McKay, Dominic J McMullan

Bristol (Bristol Genetics Service (Avon, Somerset, Gloucs and West Wilts), University Hospitals Bristol NHS Foundation Trust, St Michael's Hospital, St Michael's Hill, Bristol, BS2 8DT, UK) *Recruiting Consultant Clinical Geneticists*: Ruth Newbury-Ecob*, Sarah Smithson Research *Nurse/ Genetic Counsellors*: Rose Hawkins

Diagnostic Laboratory Scientists: Eileen Roberts, Christopher Wragg Cambridge (East Anglian Medical Genetics Service, Box 134, Cambridge University Hospitals NHS Foundation Trust, Cambridge Biomedical Campus, Cambridge ,CB2 0QQ, UK)

Recruiting Consultant Clinical Geneticists: Ruth Armstrong, Helen Firth*, Simon Holden, Sarju Mehta, Soo-Mi Park, Joan Paterson, Lucy Raymond, Richard Sandford, Geoff Woods

Research Nurse/ Genetic Counsellors: Jonathan Roberts, Sarah Wilcox

Diagnostic Laboratory Scientists: Ingrid Simonic, Becky Treacy Cardiff (Institute Of Medical Genetics, University Hospital Of Wales, Heath Park, Cardiff, CF14 4XW, UK and Department of Clinical Genetics, Block 12, Glan Clwyd Hospital, Rhyl, Denbighshire, LL18 5UJ, UK)

Recruiting Consultant Clinical Geneticists: Hayley Archer, Sally Davies, Dhavendra Kumar, Emma McCann*, Daniela T. Pilz*, Annie Procter

Research Nurse/ Genetic Counsellors: Karenza Evans Diagnostic

Laboratory Scientists: Sian Morgan, Hood Mugalaasi Dublin (National Centre for Medical Genetics, Our Lady's Children's Hospital, Crumlin, Dublin 12, Ireland)

Recruiting Consultant Clinical Geneticists: Sally Ann Lynch*

Research Nurse/ Genetic Counsellors: Rosie O'Shea Dundee (East of Scotland Regional Genetics Service, Human Genetics Unit, Pathology Department, NHS Tayside, Ninewells Hospital, Dundee, DD1 9SY, UK)

Recruiting Consultant Clinical Geneticists: Jonathan Berg*, David Goudie, Susann Schweiger Research Nurse/ Genetic Counsellors: Debbie Rice

Diagnostic Laboratory Scientists: David Baty, Norman Pratt Edinburgh (MRC Human Genetics Unit, MRC IGMM, University of Edinburgh, Western General Hospital, Edinburgh, EH4 2XU, UK)

Recruiting Consultant Clinical Geneticists: David R. FitzPatrick*, Wayne Lam, Anne Lampe *Research Nurse/ Genetic Counsellors*: Philip Greene

Diagnostic Laboratory Scientists: Eddy Maher, David Moore

Exeter (Peninsula Clinical Genetics Service, Royal Devon and Exeter NHS Foundation Trust, Clinical Genetics Department, Royal Devon & Exeter Hospital (Heavitree), Gladstone Road, Exeter, EX1 2ED, UK)

Recruiting Consultant Clinical Geneticists: Carole Brewer, Bruce Castle, Emma Kivuva*, Julia Rankin, Charles Shaw-Smith, Claire Turner, Peter Turnpenny

Research Nurse/ Genetic Counsellors: Gemma Devlin, Sarah Everest

Diagnostic Laboratory Scientists: Sian Ellard, Carolyn Tysoe Glasgow (West of Scotland Regional Genetics Service, NHS Greater Glasgow and Clyde, Institute Of Medical Genetics, Yorkhill Hospital, Glasgow, G3 8SJ, UK)

Recruiting Consultant Clinical Geneticists: Rosemarie Davidson, Carol Gardiner, Shelagh Joss, Esther Kinning, Victoria Murday, John Tolmie*, Margo Whiteford

Research Nurse/ Genetic Counsellors: Alexis Duncan

Diagnostic Laboratory Scientists: Gordon Lowther, Nicola Williams Leeds (Yorkshire Regional Genetics Service, Leeds Teaching Hospitals NHS Trust, Department of Clinical Genetics, Chapel Allerton Hospital, Chapeltown Road, Leeds, LS7 4SA, UK)

Recruiting Consultant Clinical Geneticists: Chris Bennett, Moira Blyth*, Emma Hobson, Alison Kraus, Katrina Prescott*, Audrey Smith, Jenny Thomson

Research Nurse/ Genetic Counsellors: Miranda Squires

Diagnostic Laboratory Scientists: Andrea Coates, Sarah Hewitt, Paul Roberts

Leicester (Leicestershire Genetics Centre, University Hospitals of Leicester NHS Trust, Leicester Royal Infirmary (NHS Trust), Leicester, LE1 5WW, UK)

Recruiting Consultant Clinical Geneticists: Pradeep Vasudevan*

Research Nurse/ Genetic Counsellors: Beckie Kaemba, Sandra Kazembe

Diagnostic Laboratory Scientists: Lara Cresswell

Liverpool (Merseyside and Cheshire Genetics Service, Liverpool Women's NHS Foundation Trust, Department of Clinical Genetics, Royal Liverpool Children's Hospital Alder Hey, Eaton Road, Liverpool, L12 2AP, UK)

Recruiting Consultant Clinical Geneticists: Astrid Weber*, Alan Fryer, Lynn Greenhalgh, Elizabeth Sweeney Research Nurse/ Genetic Counsellors: Gillian Roberts, Vivienne Sutton *Diagnostic Laboratory Scientists*: Angela Douglas, Una Maye

London - North West Thames (North West Thames Regional Genetics Centre, North West London Hospitals NHS Trust, The Kennedy Galton Centre, Northwick Park And St Mark's NHS Trust Watford Road, Harrow, HA1 3UJ, UK)

Recruiting Consultant Clinical Geneticists: Birgitta Bernhard, Angela Brady, Natalie Canham*, Neeti Ghali, Susan Holder, Anthony Vandersteen, Emma Wakeling

Research Nurse/ Genetic Counsellors: Cheryl Sequeira, Roldan Singzon

Diagnostic Laboratory Scientists: Louise Bourdon, Stewart Payne

London - Great Ormond Street (North East Thames Regional Genetics Service, Great Ormond Street Hospital for Children NHS Foundation Trust, Great Ormond Street Hospital, Great Ormond Street, London, WC1N 3JH, UK)

Recruiting Consultant Clinical Geneticists: Jane Hurst*, Melissa Lees, Elisabeth Rosser, Richard Scott

Research Nurse/ Genetic Counsellors: Kate Brunstrom, Georgina Hollingsworth

Diagnostic Laboratory Scientists: Lucy Jenkins, Jonathon Waters

London – Guy's (South East Thames Regional Genetics Centre, Guy's and St Thomas' NHS Foundation Trust, Guy's Hospital, Great Maze Pond, London, SE1 9RT, UK)

Recruiting Consultant Clinical Geneticists: Fiona Connell, Charu Deshpande, Frances Flinter, Melita Irving, Dragana Josifova, Shehla Mohammed*, Leema Robert

Research Nurse/ Genetic Counsellors: Tina Fendick, Caroline Langman

Diagnostic Laboratory Scientists: Caroline Ogilvie, Michael Yau

London - St George's (South West Thames Regional Genetics Centre, St George's Healthcare NHS Trust, St George's, University of London, Cranmer Terrace, London, SW17 0RE, UK) *Recruiting Consultant Clinical Geneticists*: Frances Elmslie, Tessa Homfray, Sahar Mansour*, Meriel McEntagart, Anand Saggar, Kate Tatton-Brown

Research Nurse/ Genetic Counsellors: Uruj Anjum

Diagnostic Laboratory Scientists: Karen Marks, Rohan Taylor

Manchester (Manchester Centre for Genomic Medicine, St Mary's Hospital, Central Manchester University Hospitals NHS Foundation Trust, Manchester Academic Health Science Centre, Manchester M13 9WL)

Recruiting Consultant Clinical Geneticists: Kate Chandler, Jill Clayton-Smith*, Yanick Crow, Elizabeth Jones, Bronwyn Kerr, Kay Metcalfe

Research Nurse/ Genetic Counsellors: Carina Donnelly, Zara Skitt

Diagnostic Laboratory Scientists: Lorraine Gaunt, Emma Miles Newcastle (Northern Genetics Service, Newcastle upon Tyne Hospitals NHS Foundation Trust, Institute of Human Genetics, International Centre for Life, Central Parkway, Newcastle upon Tyne, NE1 3BZ, UK)

Recruiting Consultant Clinical Geneticists: John Burn, Richard Fisher, Judith Goodship, Alex Henderson, Tara Montgomery, Miranda Splitt*, Michael Wright

Research Nurse/ Genetic Counsellors: Linda Sneddon

Diagnostic Laboratory Scientists: David Bourn, Stephen Hellens

Nottingham (Nottingham Regional Genetics Service, City Hospital Campus, Nottingham University Hospitals NHS Trust, The Gables, Hucknall Road, Nottingham NG5 1PB, UK) *Recruiting Consultant Clinical Geneticists*: Abhijit Dixit, Jacqueline Eason*, Ajoy Sarkar, Nora Shannon, Mohnish Suri

Research Nurse/ Genetic Counsellors: Ann Selby

Diagnostic Laboratory Scientists: Gareth Cross, Katherine Martin Oxford (Oxford Regional Genetics Service, Oxford Radcliffe Hospitals NHS Trust, The Churchill Old Road, Oxford, OX3 7LJ, UK)

Recruiting Consultant Clinical Geneticists: Edward Blair, Richard Gibbons, Usha Kini*, Sue Price, Debbie Shears, Helen Stewart

Research Nurse/ Genetic Counsellors: Julie Phipps, Abigail Pridham, Hellen Purnell

Diagnostic Laboratory Scientists: Susan Clasper, Anneke Seller Sheffield (Sheffield Regional Genetics Services, Sheffield Children's NHS Trust, Western Bank, Sheffield, S10 2TH, UK) *Recruiting Consultant Clinical Geneticists*: Meena Balasubramanian, Diana Johnson, Michael Parker*

Research Nurse/ Genetic Counsellors: Louise Nevitt, Stuart Ingram, Cat Taylor

Diagnostic Laboratory Scientists: Emma Shearing, Kath Smith

Southampton/Wessex (Wessex Clinical Genetics Service, University Hospital Southampton, Princess Anne Hospital, Coxford Road, Southampton, SO16 5YA, UK and Wessex Regional Genetics Laboratory, Salisbury NHS Foundation Trust, Salisbury District Hospital, Odstock Road, Salisbury, Wiltshire, SP2 8BJ, UK and Faculty of Medicine, University of Southampton)

Recruiting Consultant Clinical Geneticists: Munaza Ahmed, Diana Baralle, Amanda Collins, Nicola Foulds, Katherine Lachlan, I. Karen Temple*, Diana Wellesley

Research Nurse/ Genetic Counsellors: Lucy Harrison, Audrey Torokwa Diagnostic Laboratory scientists: David J. Bunyan, Morag N. Collinson

The Synaptopathies and Paroxysmal Syndromes (SYNaPS) Study Group

(http://neurogenetics.co.uk/synaptopathies-synaps/)

Study Group Members:

Vincenzo Salpietro[1], Stephanie Efthymiou[2], Yamna Kriouile[3], Mohamed El Khorassani[3], Mhammed Aguennouz[3], Blagovesta Karashova[4], Daniela Avdjieva[4], Hadil Kathom[4], Radka Tincheva[4], Lionel Van Maldergem[5], Wolfgang Nachbauer[6], Sylvia Boesch[6], Larissa Arning[7], Dagmar Timmann[8], Bru Cormand[9], Belen Pérez-Dueñas[10], Gabriella Di Rosa[11], Erica Pironti[11], Jatinder S. Goraya[12], Tipu Sultan[13], Salman Kirmani[14], Shahnaz Ibrahim[15], Farida Jan[15], Jun Mine[16], Selina Banu[17], Pierangelo Veggiotti[18], Michel D. Ferrari[19], Alberto Verrotti[20], Gian Luigi Marseglia[21], Salvatore Savasta[21], Barbara Garavaglia[22], Carmela Scuderi[23], Eugenia Borgione[23], Valeria Dipasquale[24], Maria Concetta Cutrupi[24], Simona Portaro[25], Benigno Monteagudo Sanchez[26], Mercedes Pineda-Marfa'[27], Francina Munell[27], Alfons Macaya[27], Richard Boles[28], Gali Heimer[29], Savvas Papacostas[30], Andreea Manole[1], Nancy Malintan[1], Maria Natalia Zanetti[1], Michael G. Hanna[1], James E. Rothman[1,31], Dimitri M. Kullmann[2], Henry Houlden[1]

Study Group Members Affiliations

[1]Department of Molecular Neuroscience, UCL Institute of Neurology, London WC1N 3BG, UK

[2]Department of Clinical and Experimental Epilepsy, UCL Institute of Neurology, London WC1N 3BG, UK

[3]Children's Hospital of Rabat, University of Rabat, Rabat 6527, Morocco

[4]Department of Paediatrics, Medical University of Sofia, Sofia 1431, Bulgaria

[5]Centre of Human Genetics, University Hospital Liege, Liege 4000, Belgium

[6]Department of Neurology, Medical University Innsbruck, Anichstrasse 35, Innsbruck 6020, Austria

[7]Department of Human Genetics, Ruhr-University Bochum, Bochum 44801, Germany

[8]Braun Neurologische Universitätsklinik Universität *Essen,* Hufelandstr 55, *Essen* D-45122, Germany

[9]Department of Genetics, Universitat de Barcelona, Barcelona 08007, Spain

[10]Hospital Sant Joan de Deu, Esplugues de Llobregat 08950, Barcelona, Spain

[11]Department of Pediatrics, University of Messina, Messina 98123, Italy

[12]Division of Paediatric Neurology, Dayanand Medical College & Hospital, Ludhiana, Punjab 141001, India

[13]Department of Paediatric Neurology, Children's Hospital of Lahore, Lahore 381-D/2, Pakistan

[14]Department of Medical Genetics, Aga Khan University Hospital, Karachi, Karachi City, Sindh 74800,

Pakistan

[15]Department of Paediatric Neurology, Aga Khan University Hospital, Karachi, Karachi City, Sindh 74800, Pakistan

[16]Department of Pediatrics, Shimane University School of Medicine, 89-1 Enya, Izumo, Shimane 693-8501, Japan

[17] Institute of Child Health and Shishu Shastho Foundation Hospital, Mirpur, Dhaka 1216, Bangladesh

[18]Pediatric Neurology Unit, V. Buzzi Children's Hospital, Via Castelvetro 32, 20154 Milan, Italy

[19]Leiden University Medical Center, Albinusdreef 2, Leiden 2333, Netherlands

[20]Paediatric Department, San Salvatore Hospital, University of L'Aquila, L'Aquila, Italy

[21]Department of Pediatrics, University of Pavia, IRCCS Policlinico "San Matteo", Pavia 27100, Italy

[22]IRCCS Foundation, Neurological Institute “Carlo Besta”, Molecular Neurogenetics, 20126 Milan, Italy

[23]Laboratorio di Neuropatologia Clinica, U.O.S. Malattie, Neuromuscolari Associazione OASI Maria SS. ONLUS – IRCCS, Via Conte Ruggero 73, 94018 Troina, Italy

[24]Department of Pediatrics, University Hospital “Gaetano Martino”, University of Messina, Messina 98123, Italy

[25]IRCCS Centro Neurolesi "Bonino Pulejo", SS113, c.da Casazza, 98124 Messina, Italy

[26]Hospital Arquitecto Marcide, Avenida de la Residencia S/N, Ferrol (A Coruña), 15401 Spain

[27]Neuropediatrics Unit, University Hospital Vall d'Hebron, Barcelona 08035, Spain

[28]Courtagen Life Sciences, 12 Gill Street Suite 3700, Woburn, MA 01801 USA

[29]Division of Pediatric Neurology, Edmond and Lily Children's Hospital, Chaim Sheba Medical Center, 52621 Ramat Gan, Israel

[30]The Cyprus Institute of Neurology and Genetics, 1683 Nicosia, Cyprus

[31]Department of Cell Biology, Yale University School of Medicine, New Haven, USA

Supplemental References

[1] Engel J., Jr. (2001). A proposed diagnostic scheme for people with epileptic seizures and with epilepsy: report of the ILAE task force on classification and terminology. Epilepsia. *42*, 796–803

[2] B, Hanefeld F, Percy A, Skjeldal O. (2002). An update on clinically applicable diagnostic criteria in Rett syndrome. Comments to Rett Syndrome Clinical Criteria Consensus Panel Satellite to European Paediatric Neurology Society Meeting, Baden Baden, Germany, 11 September 2001. Eur. J. Paediatr. Neurol. *6*, 293–297

[3] Neul JL, Kaufmann WE, Glaze DG, Christodoulou J, Clarke AJ, Bahi-Buisson N, Leonard H, Bailey ME, Schanen NC, Zappella M, et al. (2010). Rett syndrome: revised diagnostic criteria and nomenclature. Ann. Neurol. *68*, 944-50

[4] Yoo Y, Jung J, Lee YN, Lee Y, Cho H, Na E, Hong J, Kim E, Lee JS, Lee JS, et al. (2017). GABBR2 mutations determine phenotype in rett syndrome and epileptic encephalopathy. Ann. Neurol. *82*, 466-478.

[5] Srivastava S, Desai S, Cohen J, Smith-Hicks C, Barañano K, Fatemi A, Naidu S. (2018). Monogenic disorders that mimic the phenotype of Rett syndrome. Neurogenetics. *19*, 41-47

[6] Schalock, R.L., Buntinx, W.H.E., Borthwick-Duffy, S., Bradley, V., Craig, E.M., Coulter, Craig EM, Gomez SC, Lachapelle Y, Luckasson R, et al. (2010). Intellectual disability: Definition, classification, and system of supports *(11e).*

[7] Sobreira N, Schiettecatte F, Boehm C, Valle D, Hamosh A. (2015). New tools for Mendelian disease gene identification: PhenoDB variant analysis module; and GeneMatcher, a web-based tool for linking investigators with an interest in the same gene. Hum. Mutat. *36*, 425-31.

[8] Huang Z, Sun Y, Fan Y, Wang L, Liu H, Gong Z, Wang J, Yan H, Wang Y, Hu G, et al. (2018). Genetic Evaluation of 114 Chinese Short Stature Children in the Next Generation Era: a Single Center Study. Cell. Physiol. Biochem. *49*, 295-305

[9] Martín-Hernández E, Rodríguez-García ME, Chen CA, Cotrina-Vinagre FJ, Carnicero-Rodríguez P, Bellusci M, Schaaf CP, Martínez-Azorín F. (2018). Mitochondrial involvement in a Bosch-Boonstra-Schaaf optic atrophy syndrome patient with a novel de novo NR2F1 gene mutation. J. Hum. Genet. *63*, 525-528.

[10] Martín-Hernández E, Rodríguez-García ME, Camacho A, Matilla-Dueñas A, García-Silva MT, Quijada-Fraile P, Corral-Juan M, Tejada-Palacios P, de Las Heras RS, Arenas J, et al. (2016). New ATP8A2 gene mutations associated with a novel syndrome: encephalopathy, intellectual disability, severe hypotonia, chorea and optic atrophy. Neurogenetics. *17*, 259-263.

[11] Salpietro V, Lin W, Delle Vedove A, Storbeck M, Liu Y, Efthymiou S, Manole A, Wiethoff S, Ye Q, Saggar A, et al. (2017). Homozygous mutations in VAMP1 cause a presynaptic congenital myasthenic syndrome
Ann. Neurol. *81*, 597-603.

[12] Mencacci NE, Kamsteeg EJ, Nakashima K, R'Bibo L, Lynch DS, Balint B, Willemsen MA, Adams ME, Wiethoff S, Suzuki K, et al. (2016). De-Novo Mutations in PDE10A Cause Childhood-Onset Chorea with Bilateral Striatal Lesions. Am. J. Hum. Genet. *98*, 763-71.

[13] Melia TJ, Weber T, McNew JA, Fisher LE, Johnston RJ, Parlati F, Mahal LK, Sollner TH, Rothman JE. (2002). Regulation of membrane fusion by the membrane-proximal coil of the t-SNARE during zippering of SNAREpins. J. Cell. Biol. *158,* 929-40

[14] Weber T, Zemelman BV, McNew JA, Westermann B, Gmachl M, Parlati F, Söllner TH, Rothman JE. (1998). SNAREpins: minimal machinery for membrane fusion. Cell. *92*, 759-72.

[15] Shen J, Tareste DC, Paumet F, Rothman JE, Melia TJ. (2007). Selective activation of cognate SNAREpins by Sec1/Munc18 proteins. Cell. *128*, 183-95

[16] Bradford MM. (1976). A rapid and sensitive method for the quantitation of microgram quantities of protein utilizing the principle of protein-dye binding. Anal Biochem. *72*, 248-54.

[17] Walch-Solimena C, Blasi J, Edelmann L, Chapman ER, von Mollard GF, Jahn R. (1995). The t-SNAREs syntaxin 1 and SNAP-25 are present on organelles that participate in synaptic vesicle recycling. J Cell. Biol. *128*, 637-45.

[18] Takamori S, Holt M, Stenius K, Lemke EA, Grønborg M, Riedel D, Urlaub H, Schenck S, Brügger B, Ringler P, Müller SA, Rammner B, Gräter F, Hub JS, De Groot BL, Mieskes G, Moriyama Y, Klingauf J, Grubmüller H, Heuser J, Wieland F, Jahn R. (2006). Molecular anatomy of a trafficking organelle. Cell. *127,* 831-46

[19] Struck DK, Hoekstra D, Pagano RE. (1981). Use of resonance energy transfer to monitor membrane fusion. Biochemistry. *20*, 4093-9.

[20] Scott BL, Van Komen JS, Liu S, Weber T, Melia TJ, McNew JA. Liposome fusion assay to monitor intracellular membrane fusion machines. (2003). Methods Enzymol. *372,* 274-300.

[21] Stein A, Weber G, Wahl MC, Jahn R. (2009). Helical extension of the neuronal SNARE complex into the membrane. Nature. *460,* 525-8.

[22] Bussi G, Donadio D, Parrinello M. (2007). Canonical sampling through velocity rescaling. J. Chem. Phys. *126*, 014101.

[23] Hess B. (2008). P-LINCS: A Parallel Linear Constraint Solver for Molecular Simulation. J. Chem. Theory. Comput. *4,* 116-22.

[24] Pronk S, Páll S, Schulz R, Larsson P, Bjelkmar P, Apostolov R, Shirts MR, Smith JC, Kasson PM, van der Spoel D, Hess B, Lindahl E.(2013). GROMACS 4.5: a high-throughput and highly parallel open source molecular simulation toolkit. Bioinformatics. *29*, 845-54
