# Supplementary material for: Mutations in the Neuronal Vesicular SNARE VAMP2 Affect Synaptic Membrane Fusion and Impair Human Neurodevelopment
Source: Am J Hum Genet. 2019 Mar 28;104(4):721–30. doi: 10.1016/j.ajhg.2019.02.016 (PMC6451933; doi:10.1016/j.ajhg.2019.02.016)
Supplement: Document S2. Article plus Supplemental Data [file mmc7.pdf]

Mutations in the Neuronal Vesicular SNARE *VAMP2* Affect Synaptic Membrane Fusion and Impair Human Neurodevelopment

Vincenzo Salpietro,[1,2,3,24] Nancy T. Malintan,[4,24] Isabel Llano-Rivas,[5] Christine G. Spaeth,[6] Stephanie Efthymiou,[3,4] Pasquale Striano,[1,2] Jana Vandrovcova,[3] Maria C. Cutrupi,[7] Roberto Chimenz,[7] Emanuele David,[8] Gabriella Di Rosa,[9] Anna Marce-Grau,[10] Miquel Raspall-Chaure,[10] Elena Martin-Hernandez,[11] Federico Zara,[12] Carlo Minetti,[1,2] Deciphering Developmental Disorders Study, SYNAPS Study Group, Oscar D. Bello,[4] Rita De Zorzi,[13] Sara Fortuna,[14] Andrew Dauber,[15] Mariam Alkhawaja,[16] Tipu Sultan,[17] Kshitij Mankad,[18] Antonio Vitobello,[19,20] Quentin Thomas,[20] Frederic Tran Mau-Them,[19,20] Laurence Faivre,[20,21] Francisco Martinez-Azorin,[22] Carlos E. Prada,[6] Alfons Macaya,[10] Dimitri M. Kullmann,[4] James E. Rothman,[4,23] Shyam S. Krishnakumar,[4,23,*] and Henry Houlden[3,*]

VAMP2 encodes the vesicular SNARE protein VAMP2 (also called synaptobrevin-2). Together with its partners syntaxin-1A and synaptosomal-associated protein 25 (SNAP25), VAMP2 mediates fusion of synaptic vesicles to release neurotransmitters. VAMP2 is essential for vesicular exocytosis and activity-dependent neurotransmitter release. Here, we report five heterozygous *de novo* mutations in *VAMP2* in unrelated individuals presenting with a neurodevelopmental disorder characterized by axial hypotonia (which had been present since birth), intellectual disability, and autistic features. In total, we identified two single-amino-acid deletions and three non-synonymous variants affecting conserved residues within the C terminus of the VAMP2 SNARE motif. Affected individuals carrying *de novo* non-synonymous variants involving the C-terminal region presented a more severe phenotype with additional neurological features, including central visual impairment, hyperkinetic movement disorder, and epilepsy or electroencephalography abnormalities. Reconstituted fusion involving a lipid-mixing assay indicated impairment in vesicle fusion as one of the possible associated disease mechanisms. The genetic synaptopathy caused by *VAMP2 de novo* mutations highlights the key roles of this gene in human brain development and function.

Chemical synaptic transmission relies on precisely coordinated, activity-dependent neurotransmitter release.[1] A fundamental step in this pathway is the fusion of synaptic vesicles with the presynaptic plasma membrane. Soluble N-ethylmaleimide-sensitive factor attachment protein receptor (SNARE) proteins mediate membrane fusion and are essential for the fusion of synaptic vesicles.[1,2] At mammalian central nervous system (CNS) synapses, neuronal SNAREs consist of vesicle-associated membrane protein 2 (VAMP2, also called synaptobrevin-2) on the vesicle membrane (v-SNARE) and the binary complex of syntaxin1A (STX1A) and synaptosomal-associated protein 25 Kd (SNAP25) on the plasma membrane (target or t-SNARE).[3] The v- and t-SNARE proteins assemble in a polarized manner starting from the N termini distal from the membranes and proceeding towards the C termini and are held together by discrete interacting residues (numbered -7 to +8), including 15 hydrophobic contacts and central ionic residues.[4] This "zippering" process pulls the membranes together and provides the energy to fuse

[1]Pediatric Neurology and Muscular Diseases Unit, IRCCS Istituto Giannina Gaslini, Genoa 16147, Italy; [2]Department of Neurosciences, Rehabilitation, Ophthalmology, Genetics, Maternal and Child Health, University of Genoa, Genoa 16132, Italy; [3]Department of Molecular Neuroscience, UCL Institute of Neurology, University College London, London WC1N 3BG, UK; [4]Department of Clinical and Experimental Epilepsy, UCL Institute of Neurology, University College London, London WC1N 3BG, UK; [5]Department of Medical Genetics, Hospital Universitario Cruces, Greater Bilbao 48903, Spain; [6]Division of Human Genetics, Department of Pediatrics, University of Cincinnati, Cincinnati Children's Hospital Medical Center, Cincinnati, Ohio 45229-3026, USA; [7]Division of Human Genetics, Department of the Adult and Developmental Age Human Pathology, University of Messina, Messina 98125, Italy; [8]Papardo University Hospital, Viale Ferdinando Stagno d'Alcontres, Contrada Papardo, Messina 98158, Italy; [9]Division of Child Neurology and Psychiatry, Department of the Adult and Developmental Age Human Pathology, University of Messina, Messina 98125, Italy; [10]Department of Pediatric Neurology, University Hospital Vall d'Hebron, Barcelona 08035, Spain; [11]Unidad de Enfermedades Mitocondriales-Metabólicas Hereditarias, Departamento de Pediatría, Hospital 12 de Octubre, Madrid 28041, Spain; [12]Laboratory of Neurogenetics and Neuroscience, G. Gaslini Institute, Genova 16147, Italy; [13]Center of Excellence in Biocrystallography, Department of Chemical and Pharmaceutical Sciences, University of Trieste, Trieste 34127, Italy; [14]Department of Chemical and Pharmaceutical Sciences, University of Trieste, Trieste 34127, Italy; [15]Division of Endocrinology, Cincinnati Center for Growth Disorders, Cincinnati Children's Hospital Medical Center, Cincinnati, Ohio 45229-3026, USA; [16]Prince Hamzah Hospital, Ministry of Health, Amman 11181, Jordan; [17]Department of Pediatric Neurology, Institute of Child Health and The Children's Hospital Lahore, 381-D/2, Lahore 54600, Pakistan; [18]Department of Neuroradiology, Great Ormond Street Hospital for Children, London WC1N 3JH, UK; [19]Unité Fonctionnelle Innovation en Diagnostic Genomique des Maladies Rares, Center Hospitalier Universitaire Dijon Bourgogne, Dijon 21079, France; [20]Inserm, UMR 1231, Genetique des Anomalies du Development, Université de Bourgogne, Dijon 21079, France; [21]Center de Référence Anomalies du Développement et Syndromes Malformatifs, Hôpital d'Enfants, Dijon 21079, France; [22]Centro de Investigación Biomédica en Red de Enfermedades Raras (CIBERER), Instituto de Investigación Hospital 12 de Octubre (i+12), Madrid 28041, Spain; [23]Department of Cell Biology, Yale University School of Medicine, New Haven, CT 06520, USA
[24]These authors contributed equally
*Correspondence: s.krishnakumar@ucl.ac.uk (S.S.K.), h.houlden@ucl.ac.uk (H.H.)
https://doi.org/10.1016/j.ajhg.2019.02.016.
© 2019 The Authors. This is an open access article under the CC BY license (http://creativecommons.org/licenses/by/4.0/).

the lipid bilayers.[5,6] The SNAREs alone are sufficient to drive fusion of synaptic vesicles, but this process is tightly regulated by a number of synaptic proteins to enable Ca^{2+}-regulated neurotransmitter release.[7] The key regulatory elements at excitatory CNS synapses include chaperones (Munc18 and Munc13), the primary Ca^{2+} sensor synaptotagmin-1, and the auxiliary protein complexin.[7–10]

VAMP2 (MIM: 185881) encodes a neuronal v-SNARE essential for the fusion of synaptic vesicles at mammalian central nerve terminals.[5–7] Introduction of specific engineered mutations affecting its SNARE motif has been reported to alter vesicle fusion *in vitro* by impairing either formation of the SNARE complex or the interaction of VAMP2 with other (auxiliary) presynaptic proteins.[11,12] *Vamp2*$^{-/-}$ mice present severely decreased rates of both spontaneous and Ca^{2+}-triggered synaptic-vesicle fusion, and these mice die immediately after birth.[13] Also, synapses from VAMP2-deficient mice display changes in synaptic-vesicle morphology and size—and delayed stimulus-dependent endocytosis.[14] Thus, VAMP2 exerts a complex influence on synaptic transmission; it plays fundamental roles in vesicle fusion, neurotransmitter release, and vesicle endocytosis. Despite the critical role of VAMP2 in presynaptic molecular events, little is known of the consequences of disrupted *VAMP2* function in human neurodevelopment.

Here, we describe five unrelated individuals who had shown hypotonia since birth and who had intellectual disability (ID) with autistic features, including variable motor stereotypies resembling Rett syndrome (RTT), and, in some children, also central visual impairment, hyperkinetic movements, and epilepsy and/or electroencephalography (EEG) abnormalities. Table 1 summarizes the detailed phenotypes of the individuals (1–5), aged between 3 and 14 years.

In all affected children, family histories, pregnancies, and birth histories were unremarkable, and neurodevelopmental impairment occurred within the first year of life. The earliest sign of neurological involvement was axial hypotonia at birth. Poor visual fixation (with only brief and occasional visual contact, lasting up to a few seconds) had been evident since the first months of life in three affected individuals (1–3); these individuals were later diagnosed with central visual impairment (Table 1). Three children (individuals 1–3) exhibited a hyperkinetic movement disorder starting in the first year of life (Videos S1, S2, S3, and S4). Abnormal movements ranged from dystonic posturing (mainly involving the trunk, neck, and lower limbs) and moderate chorea (individuals 1 and 3) to a mixed-movement disorder with severe chorea and dystonic posturing (individual 2) or myoclonic jerks (individual 3). All children showed autistic features, typically including flapping or flailing of the arms, as well as hand wringing or clapping. Additional repetitive behavior patterns included body rocking and head banging. Self-injurious behaviors were evident in individual 2. A virtual absence of purposeful hand movements was present in all cases (Table 1, Videos S1, S2, S3, S4, and S5). Motor development in individuals 1–3 was severely impaired, and these children had not attained the ability to walk. Severe language impairment was present in the three more severely affected children (individuals 1–3), none of whom had attained meaningful speech production, but individuals 4 and 5 were capable of saying 5–10 words (Table 1).

Seizures or abnormal EEG occurred in four affected individuals. Individual 1 did not present with epileptic seizures, but ictal EEG recording at the age of 15 months showed high-voltage delta activity with interspersed sharp-and-slow-wave complexes over the right central and posterior brain regions. Individual 2 suffered from multiple focal seizures per day; these started shortly after birth and were characterized on EEG by fast rhythmic activity followed by sharp-and-slow-wave complexes (Figure S1). At 12 months, individual 3 presented with infantile spasms that were associated with diffuse EEG paroxysms. Individual 4 developed infrequent staring episodes with eyelid myoclonia at 5 years of age and had a single episode of non-convulsive *status epilepticus* at the age of 11 years. Several anti-epileptic drugs, including valproic acid, vigabatrin, and lamotrigine, have been trialed in individuals 2–4 (see Supplemental Data); beneficial effects of valproic acid treatment were noted in individual 4, who has been seizure-free since the age of 12 years and has had normal follow-up EEGs. Individual 2 underwent a craniotomy for grid placement at the age of 6 months and had a right posterior circulation stroke affecting the thalamic and cortical areas; at the age of 18 months, he had a right temporal lobectomy. Brain magnetic resonance imaging (MRI) was unrevealing in all children except in individual 1, for whom mild myelination delay and a posteriorly slender corpus callosum was observed at the age of 2 years (Figure 1).

The clinical features summarized above are consistent with a diagnosis of neurodevelopmental impairment with variable neurological features in all five affected individuals. Extensive initial genetic and biochemical diagnostic investigations for a range of genetic conditions, including non-syndromic ID, epileptic encephalopathies (EEs), EEs with dyskinesia, metabolic disorders, and mitochondrial diseases, were unrevealing (see Supplemental Data). Affected children were recruited for genetic analysis through the use of whole-exome sequencing (WES) at five centers. Written informed consent was obtained for all individuals and their relatives, after which DNA was extracted from peripheral lymphocytes according to standard protocols. The study was approved by the local ethics committee at University College London Hospitals (project 06/N076) and at the participating institutions. Variants of interest in *VAMP2* were identified by WES of trios and confirmed by Sanger sequencing in all cases. Libraries were prepared from parents' and affected individuals' DNA, and exomes were captured and sequenced on Illumina sequencers. Raw data were processed and filtered with established pipelines and then annotated, and the Exome variant server ESP6500 was used for assessments

Table 1. Clinical Features of Individuals with *De Novo VAMP2* Mutations

Individual Number Gender Age	Variant	Growth/ OFC	Hypotonia/ DD	ID	Epileptic Seizures	EEG	ASD	RTT-Like Features	Movement Disorder	Central Visual Defects	Speech Impairment	Brain Imaging	Additional Features
1 F 3 yr	c.223T>C, p.Ser75Pro	normal	yes	severe	no	high-voltage delta activity, sharp wave-slow wave complexes	yes	stereotyped hand movements, absent purposeful hand movements	choreic movement, flapping, dystonic postures	yes	absent speech	thin corpus callosum, delayed myelination	inability to walk
2 M 10 yr	c.233A>C, p.Glu78Ala	normal	yes	severe	focal seizures, GTCS	fast rhythmic activity, sharp wave-slow wave complexes	yes	body rocking, head banging, screaming, absent purposeful hand movements	generalized chorea	yes	absent speech	unremarkable	abnormal behavior, self-injury, inability to walk
3 M 13yr	c.230T>C, p.Phe77Ser	normal	yes	severe	infantile spasms, convulsive status epilepticus	disorganized EEG paroxysms	yes	stereotyped hand movements, absent purposeful hand movements	choreic movement, myoclonic jerks	yes	absent speech	unremarkable	abnormal behavior, inability to walk, severe constipation
4 M 14yr	c.128_130delTGG, p.Val43del	normal	yes	moderate	focal seizures	generalized and multifocal abnormalities	yes	stereotyped hand movements (wringing), absent purposeful hand movements	no	no	only 5–10 spoken words	unremarkable	clumsiness, abnormal behavior
5 F 3 yr	c.135_137delCAT, p.Ile45del	normal	yes	moderate	no	disorganized EEG paroxysms	yes	stereotyped hand movements (washing)	no	no	only 5 spoken words	unremarkable	abnormal behavior

Abbreviations are as follows: ASD = autism spectrum disorder; DD = developmental delay; EEG = electroencephalography; FC = focal seizures; GTCS = generalized tonic-clonic seizures; ID = intellectual disability; and OFC = occipital-frontal circumference. Variants are named according to the GenBank: NM_014232 reference transcript.

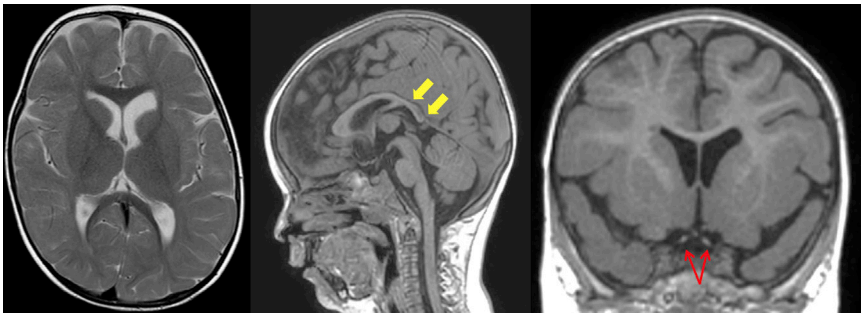

Figure 1. Brain MRI Scan of Individual 1, Who Harbors a *De Novo* VAMP2 p.Ser75-Pro Variant, at the Age of 2 Years
The panel shows axial T2-weighted, sagittal T1-weighted, and coronal T1-weighted MR images. There is some generalized delay in the maturation of myelin and a reduced volume of the cerebral white matter posteriorly. (Yellow arrows show a posteriorly slender corpus callosum.) The optic nerves and chiasm are hypoplastic (red arrows).

of variant frequency in the control population (see Supplemental Data). Only exonic and donor and acceptor splicing variants were considered. Priority was given to rare variants (that had a genomic evolutionary rate profiling [GERP] score >2 and were present at <1% in public databases, including those of the 1000 Genomes Project, NHLBI Exome Variant Server, Complete Genomics 69, and Exome Aggregation Consortium [ExAC v0.2]). Synonymous variants were not considered. Following their respective analysis pipelines,[15–18] participating centers generated a list of candidate variants filtered against variants from public databases according to modes of inheritance, then compared their results through international research networks and variant databases.[19,20]

Three *de novo* non-synonymous variants in *VAMP2* [NM_014232: c.223T>C (p.Ser75Pro), c.230T>C (p.Phe77Ser), c.233A>C (p.Glu78Ala)] were identified in three affected individuals (1–3) recruited and studied at different centers as part of different research initiatives (see Supplemental Data). We then analyzed the genetic data from the SYNaPS Study Group collection of exomes and genomes from over 4,000 individuals affected with early-onset neurological disorders (including ~250 children with undiagnosed neurodevelopmental impairment and epilepsy) for variants in *VAMP2* and identified a child (individual 4), carrying a *de novo* single amino acid deletion at position 43 [NM_014232: c.128_130delTGG (p.Val43del)] (Figures 2A and 2B). We next used web-based tools[19,20] to screen *VAMP2* variants within exome and genome datasets from established international collaborations; this process identified an additional child (individual 5) carrying a *de novo* single-amino-acid deletion at position 45 [GenBank: NM_014232, c.135_137delCAT (p.Ile45del)] (see Supplemental Data).

All the identified variants were absent from the Genome Aggregation Database and ExAC, and all displayed high conservation (mean: $GERP^{++}$ 5.26) and *in silico* pathogenic predictor (mean: CADD_Phred 26.9) scores (see Supplemental Data). In the ExAC database (last accessed January 30, 2018), which contains exomes from 60,706 unrelated individuals, there are no listed loss-of-function variants in *VAMP2,* and only two non-synonymous variants (p.Asn49Lys [p.Val50Met]) are present within the SNARE motif (amino acids 31–91).

The *de novo* non-synonymous variants identified in this study cluster in close proximity within the C-terminal portion of the SNARE motif (Figure 2C). Interspecies alignment of protein sequences generated with Clustal Omega show that all mutations occur within the SNARE motif at residues highly conserved through evolution (Figure 2D). Figure 3 shows positions of the mutated amino acids within a 3D structure of the VAMP2 ectodomain in complex with STX1A and SNAP25. Replacement analysis shows that the p.Ser75Pro variant will result in the loss of two hydrogen bonds, one interchain between Ser75 of VAMP2 and Tyr243 of STX1A and one intrachain between Ser75 and Gln71, although the p.Phe77Ser variant introduces a hydrophilic residue in an otherwise hydrophobic region and the p.Glu78Ala variant disrupts the hydrogen bond between Glu78 of VAMP2 and Arg246 of STX1A.

To determine whether these disease-associated variants affect VAMP2 structure and SNARE complex stability, we performed 100 ns molecular dynamics (MD) simulations by using a humanized version of the neuronal SNARE complex (PDB 3HD7, see Supplemental Data). During the simulations, the WT and p.Ser75Pro seemed to reach a stationary state, but major rearrangements were still observed for p.Phe77Ser and p.Glu78Ala at the end of the simulation. This was evident in their backbone root-mean-square deviation (RMSD) and radius of gyration, which measure the divergence of the mutant protein structure from its initial structure over the course of the simulation. In all cases, the most mobile portion of the chain was that close to the C terminus, as seen in their root mean squared fluctuation (RMSF). The RMSF further indicates that in all cases, the variants increase the mobility of the backbone, and this effect is particularly evident for p.Glu78Ala. Overall rearrangements of the complex are shown in Figures S2–S3.

To examine *VAMP2* expression across CNS regions, we used microarray data (Affymetrix Exon 1.0 ST) from human post-mortem brain tissues as previously described.[21] This analysis showed the highest *VAMP2* expression in the putamen and the frontal lobes (Figure S4).

To evaluate the functional consequence of *VAMP2* variants, we employed the reconstituted, lipid-mixing assay based on NBD (N-[7-nitro-2-1, 3-benzoxadiazol-4-yl])-to-RHO (lissamine rhodamine B) energy transfer (see Supplemental Data). In this assay, the VAMP2 (wild-type [WT] or mutant) was included in the fluorescent donor liposomes, whereas the t-SNAREs were reconstituted into the non-fluorescent acceptor liposomes. We read out membrane

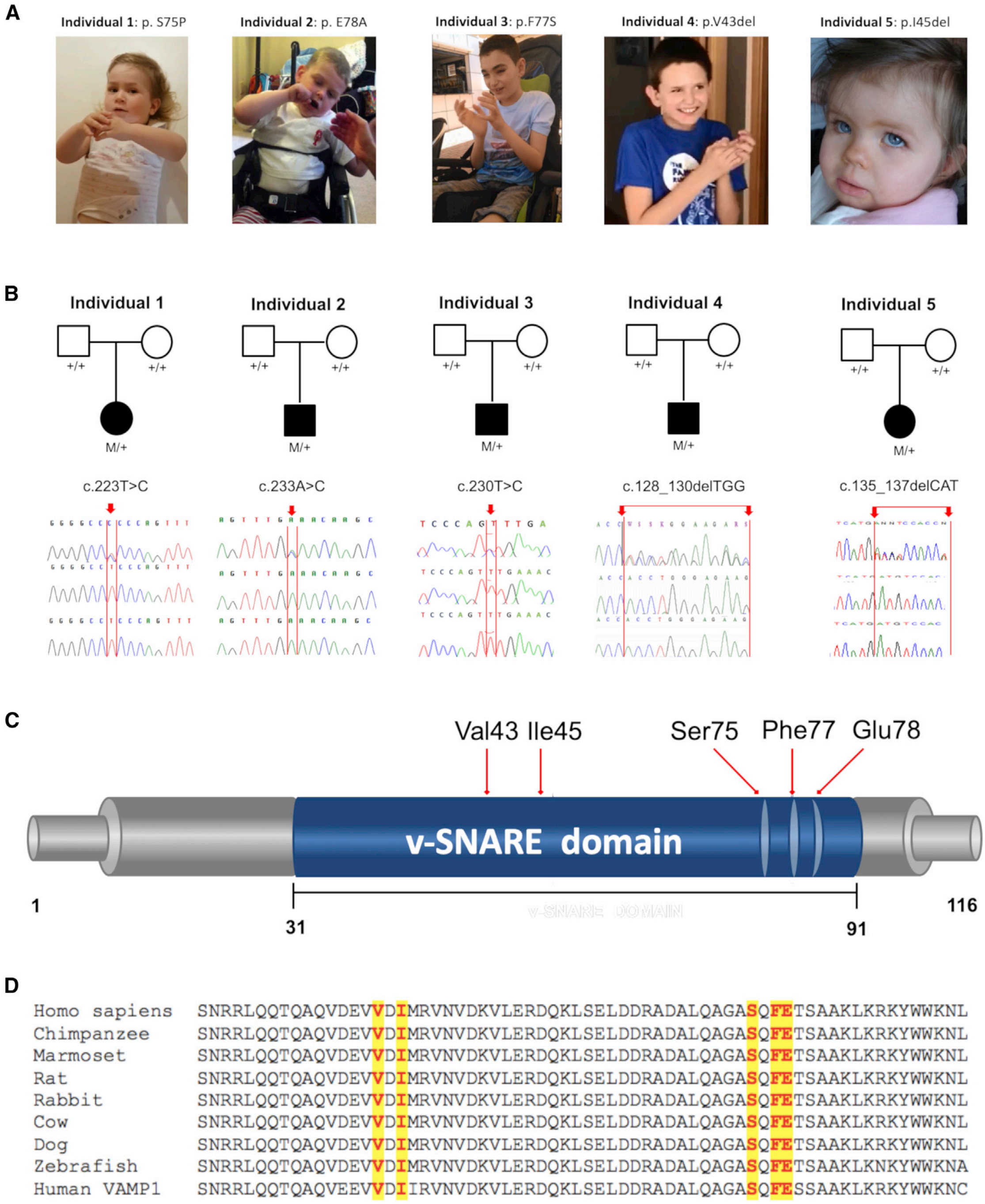

Figure 2. *VAMP2* Intragenic *De Novo* Variants Identified in This Study

(A) Individuals carrying *de novo VAMP2* intragenic variants; note the hand stereotypies.

(B) Sanger sequences of five kindreds with *de novo VAMP2* intragenic variants. Chromatograms of individuals 1–5 and their parents confirm the *de-novo* occurrence of the *VAMP2* variants in all cases. M/+ denotes the indicated *VAMP2* variant in the heterozygous state, and +/+ denotes homozygous wild-type sequence. Mutant bases in the probands are indicated by a red arrow.

(C) Schematic depiction of the human VAMP2 protein (GenBank: NP_055047.2) indicating the positions of the variants identified in this study.

(D) Multiple alignment showing complete conservation across species and VAMP1 homolog (GenBank: NP_055046.1) of the residues affected by the variants identified in this study (these variants are highlighted in yellow). Human VAMP2 (GenBank: NP_055047.2), chimpanzee VAMP2 (UniProt: JAA33755.1), marmoset VAMP2 (UniProt: JAB33896.1), rat VAMP2 (NP_036795.1), rabbit VAMP2 (XP_008268978.1), cow VAMP2 (GenBank: NP_776908.1), dog VAMP2 (GenBank: XP_005620068.1), zebrafish VAMP2 (GenBank: NP_956299.1).

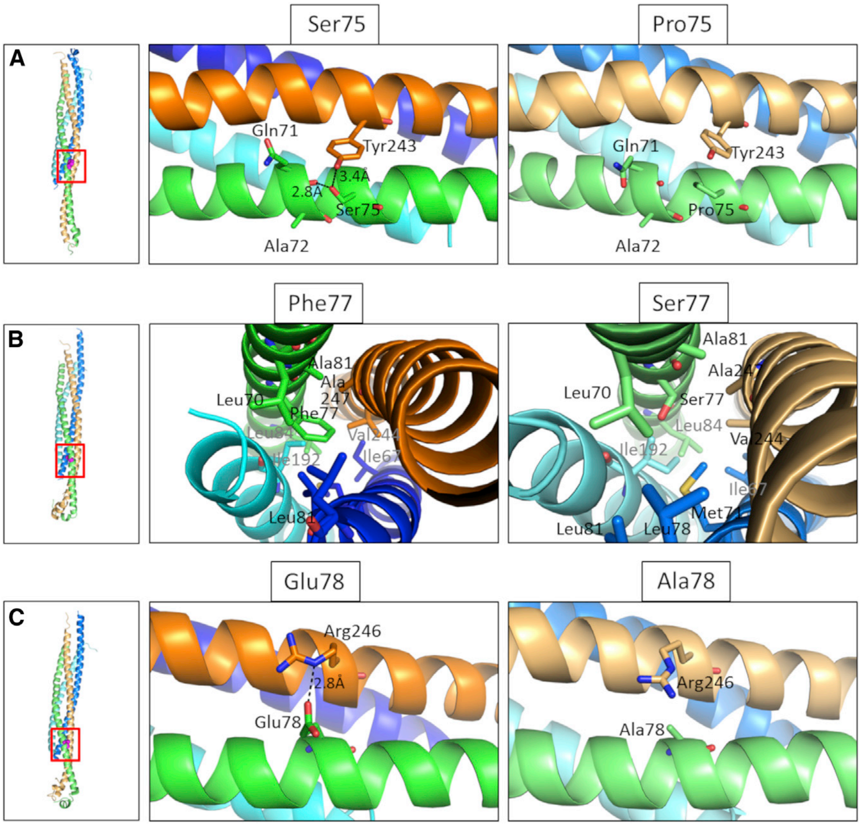

Figure 3. Molecular Modeling of the Identified *De Novo* VAMP2 Non-Synonymous Variants

Comparison between the p.Ser75Pro (A), p.Phe77Ser (B), and p.Glu78Ala (C) mutant conformation within the SNARE complex (left panel, red square). The wild-type conformation is shown in the middle panel, and the mutated residues are shown in the right panel. Variant p.Ser75Pro causes the loss of two hydrogen bonds, one interchain between Ser75 of VAMP2 and Tyr243 of STX1A and one intrachain between Ser75 and Gln71; variant p.Phe77Ser introduces a hydrophilic residue in an otherwise hydrophobic region; and variant p.Glu78Ala causes the loss of a hydrogen bond between Glu78 of VAMP2 and Arg246 of STX1A. Modeling of the VAMP2 ectodomain (green for WT, light green for mutants) in complex with STX1A (orange for WT, light orange for mutants) and Snap25 (blue and cyan for WT, marine and aquamarine for mutants); configurations are as seen 100 ns into the molecular dynamic simulation. The complexes were modeled from the humanized 3HD7 complex. Water molecules and ions are not shown.

fusion between the donor and acceptor liposome mixing by quantifying increased fluorescence resulting from the dequenching of NBD fluorescence (Figure 4A). To this end, we purified WT VAMP2 and the variant protein along with the t-SNARE complex by using a bacterial expression system as previously described.[22,23] We were able to purify the p.Ser75Pro and p.Glu78Ala variants, and Coomassie-stained SDS-PAGE analysis showed that these variants were structurally intact and highly pure with no contamination (Figure S5). However, all attempts to isolate the p.Phe77Ser were unsuccessful. We therefore limited our *in vitro* fusion analysis to the two remaining non-synonymous variants (p.Ser75Pro and p.Glu78Ala).

As shown in Figures 4C–4F, the VAMP2 disease-associated variant p.Ser75Pro reduced the rate and extent of fusion compared to that seen with VAMP2 WT, whereas the p.Glu78Ala variant had little to no effect (Figures 4C and 4D). The reduction in the fusion associated with p.Ser75Pro was estimated to be approximately 25% that in the WT, suggesting that the introduction of a proline residue at this site most likely interferes with the proper assembly of the SNARE proteins and thus affects VAMP2 fusion properties, whereas the fusion profile associated with the p.Glu78Ala was indistinguishable from that of the WT.

Earlier studies have shown that Munc18 chaperones SNARE assembly via interactions with the VAMP2 C-terminal region.[12,24] We therefore investigated the effect of the disease variants under Munc18-activated conditions. As expected, inclusion of Munc18-1 produced an approximately 2-fold increase in the rate and extent of fusion when WT VAMP2 was used (Figure 4E). Strikingly, Munc18 could not activate the fusion mediated by the VAMP2 p.Ser75Pro variant (Figure 4E). Consequently, we observed a significant (>90%) loss-of-function phenotype with the p.Ser75Pro variant under these conditions. In contrast, Munc18 was able to activate the fusion mediated by VAMP2 p.Glu78Ala, confirming that this variant does not affect the SNARE assembly process or its activation. To accurately emulate the physiological make-up of the individuals carrying heterozygous *de novo VAMP2* variants, we also tested the effect of replacing half the copies of WT VAMP2 with the disease variants (Figure S4). Remarkably, in the case of p.Ser75Pro, the fusion profile for the mixed v-liposomes (50:50 WT:mutant) was identical to the fusion profile for the homogenous samples containing only the mutant proteins (Figure 4F; Figure S4). This implies that p.Ser75Pro mutant dominantly interferes with WT (Figure 4F), and this could readily explain the pathological phenotype observed with this variant.

Our genetic and functional studies show that *de novo* mutations in *VAMP2* cause neurodevelopmental impairment associated with variable clinical features. Individuals 1–3, carrying *de novo* non-synonymous variants affecting the C terminus of the VAMP2 SNARE motif (residues 75, 77, and 78), presented a severe neurological phenotype with motor impairment (and inability to walk), central visual deficits, hyperkinetic movements, and, in two of

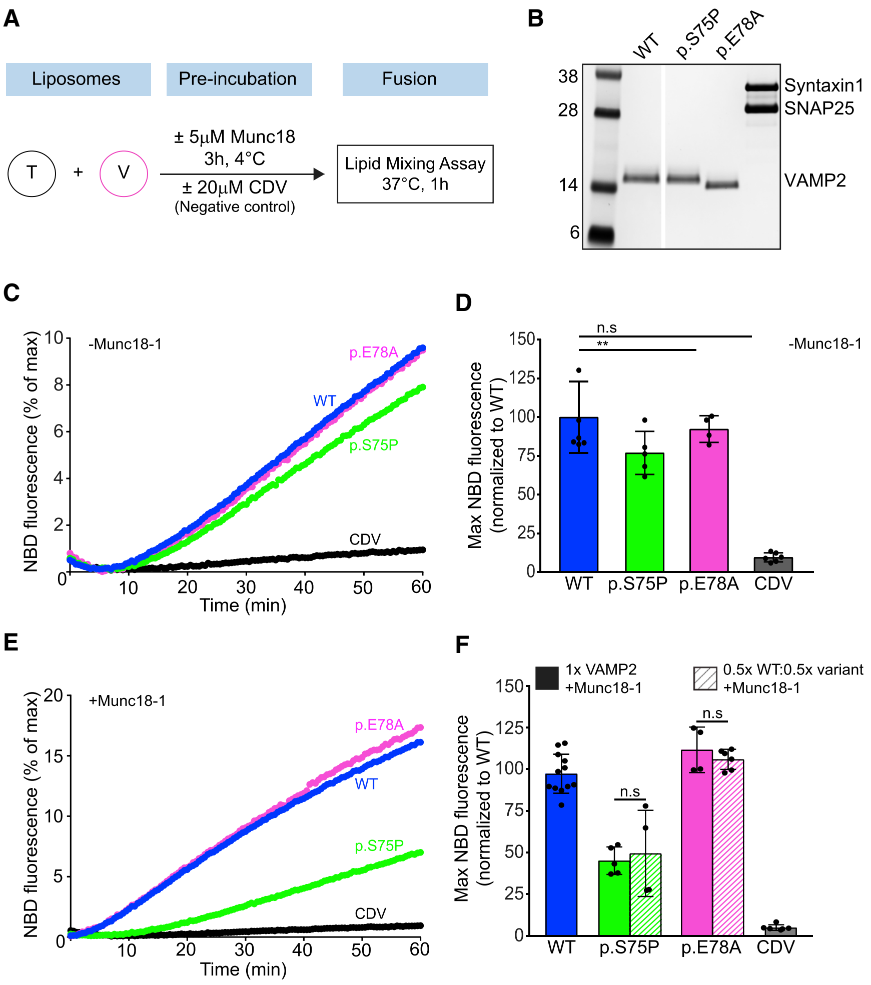

Figure 4. Disease-Associated *VAMP2* Variants Result in Reduced Fusion Rates

(A) Scheme showing the liposome fusion assay.

(B) The SDS-PAGE and Coomassie-stained gel image of VAMP2 WT, VAMP2 disease-associated variants (p.Ser75Pro [p.Glu78Ala]), and t-SNARE (syntaxin 1 and SNAP25) reconstitution into donor v- and acceptor t-liposomes, respectively.

(C) Line graphs showing the average basal (without Munc18-1) increase that occurs in NBD fluorescence as a result of fusion between the v-liposome and t-SNARE liposomes carrying WT or VAMP2 disease variants (p.Ser75Pro [p.Glu78Ala]). Liposome fusion reaction in the presence of CDV was used as negative control.

(D) Basal fusion quantification, normalized to WT, at the endpoint (60 min) as described in (C).

(E) Line graphs of liposome fusion reaction as in (C), in the presence of 5 μM Munc18-1.

(F) Endpoint fusion quantification, normalized to WT, (60 min) of experiment as described in (E). Bar graphs also showed endpoint quantification of a similar experiment that used a v-liposome that contained a mixture of WT and mutant VAMP2 proteins. Data were from at least four independent replicates and presented as means plus SD. $^{*}p < 0.05$; $^{**}p < 0.01$; $^{***}p < 0.001$; n.s., not significant ($p > 0.05$).

them, epilepsy starting in infancy. Individuals 4 and 5, carrying *de novo* single-amino-acid deletions involving residues at positions 43 and 45, presented a less severe neurological involvement, acquired the ability to walk, and were able to pronounce a few words. MD simulations showed that missense mutations in the C terminus induce higher flexibility of this region within the assembled SNARE complexes. The *in vitro* lipid-mixing assay revealed a significant defect in vesicle fusion as a consequence of the p.Ser75Pro variant, but p.Glu78Ala had no clear functional consequence. The pathophysiological phenotype for the p.Glu78Ala variant might be due to impaired interactions with regulatory proteins that were not included in the *in vitro* assay. Notably, the assembly of the C-terminal region of the SNARE proteins is considered critical to driving membrane fusion,[5,25] and several synaptic regulatory proteins modulate vesicle fusion by binding the C-terminal portion of the SNARE complex.[12,23,24] Thus, mutations affecting this region could disturb the SNARE complex assembly by less-efficient partnering of cognate SNARE proteins and/or disrupt its association with regulatory elements such as Munc18-1 or Synaptotagmin. In the physiological context, this would manifest as the perturbation of Ca^{2+}-triggered neurotransmitter release. Even a slight alteration of the fusion kinetics *in vitro* would translate to a dramatic effect on the release of neurotransmitters release at the neuronal synapses. This might explain the severe neurodevelopmental impairment observed in the VAMP2 synaptopathy. Interestingly, variants affecting the Ser75 residue have previously been shown to impair the Munc18-1 stimulatory activity by impairing its ability to regulate trans-SNARE zippering,[12,23] and variants involving residue Glu78 can also affect Ca^{2+}-regulated neurotransmitter release.[26]

The present work adds to the evidence that neurodevelopmental disorders (NDDs) have a strong genetic component and encompass a range of frequently co-existing conditions, including ID, developmental delay (DD), and autism spectrum disorders (ASDs).[27,28] Neurodevelopmental impairment, epilepsy, and movement disorders also frequently co-exist.[29,30] Rare variants in genes that encode a number of presynaptic proteins involved in Ca^{2+}-regulated neurotransmitter release have been identified in individuals affected by a spectrum of neurological disorders. These include the following:

1. variants in *SNAP25* (MIM: 60322) isoforms *SNAP25a* and *SNAP25b;* these variants have been identified in association with ID, seizures, and myasthenia[31,32]
2. variants in *SYT1* (MIM: 185605), which encodes the Ca^{2+}-sensor synaptotagmin-1 required for evoked

synchronous fusion; these variants are found in individuals with NDDs and hyperkinetic movements[33,34]
3. variants in genes encoding the RIM interactor PNKD or the SNAP25 and synaptotagmin-1 interactor PRRT2; these variants have been identified in different forms of dyskinesias and seizures (MIM: 128200; MIM: 60575)[35,36]
4. variants in *UNC13A* (MIM: 609894), encoding the synaptic regulator Munc13-1; these variants have been linked to an NDD with involuntary movements[37]
5. variants in *STXBP1* (MIM: 602926), encoding Munc18-1; these variants cause NDDs with epilepsy and autistic features[38]

The phenotypes associated with the VAMP2 synaptopathy reported here are reminiscent of the variability reported in some individuals who have *de-novo* variants in *STXBP1* or in *SYT1* and who can present with a combination of neurodevelopmental impairment, stereotypies, hyperkinetic movements (including chorea and dystonia), and EEG anomalies or epileptic syndromes of variable severity.[33,39]

Notably, a heterozygous mutation in a synaptobrevin homolog, *VAMP1*, which encodes a protein involved in vesicle fusion mainly at neuromuscular synapses,[40] has been linked to spastic ataxia in families from Newfoundland.[41] More recently, biallelic mutations in *VAMP1* have been identified in association with a phenotype of congenital hypotonia and muscle weakness, and in three of these families neurophysiological evidence of presynaptic neuromuscular transmission impairment was detected and led to a diagnosis of presynaptic congenital myasthenic syndrome.[42–44]

In conclusion, we have identified a neurodevelopmental disease that is variably associated with additional neurological features, including epilepsy and hyperkinetic movements, and that is caused by *de novo* mutations in *VAMP2*. These results further delineate an emerging spectrum of human core synaptopathies caused by variants in genes that encode SNAREs and essential regulatory components of the synaptic machinery. The hallmark of these disorders is impaired presynaptic neurotransmission at nerve terminals; this impaired neurotransmission results in a wide array of (often overlapping) clinical features, including neurodevelopmental impairment, weakness, seizures, and abnormal movements. The genetic synaptopathy caused by *VAMP2* mutations highlights the key roles of this gene in human brain development and function. Variability in the effects of different *VAMP2* mutants under *in vitro* conditions points toward mutation-specific mechanisms underlying the presynaptic defect of the affected children, and this variability highlights a promising area of future research.

Accession Numbers

The accession numbers for the DNA sequences reported in this paper are in the Leiden Open Variation Database: 00181522, 00181523, 00181524, 00181525, 00181526.

Supplemental Data

Supplemental Data can be found online at https://doi.org/10.1016/j.ajhg.2019.02.016.

Acknowledgements

We gratefully acknowledge all the families for their enthusiastic participation in this study. This study was supported by the Wellcome Trust (WT093205MA, WT104033AIA); the Medical Research Council (H.H. and D.M.K.); the European Community's Seventh Framework Programme (FP7/2007-2013, under grant agreement No. 2012-305121 to H.H.); the Italian CINECA Awards (HP10CRVL7F, 2017), which made available high-performance computing resources and provided support; the Spanish Instituto de Salud Carlos III (ISCIII) (PI15/01791 A.M.); and the European Regional Development Fund (ERDF) (PI17/00487 to F.M-A.). We are also supported by the National Institute for Health Research (NIHR), the University College London Hospitals (UCLH), and the Biomedical Research Center (BRC).

Declaration of Interests

The authors declare no competing interests.

Received: October 10, 2018
Accepted: February 13, 2019
Published: March 28, 2019

Web Resources

CADD, https://cadd.gs.washington.edu/
ClustalX, https://www.ebi.ac.uk/Tools/msa/clustalw2/
Ensembl, http://www.ensembl.org/
Exome Aggregation Consortium (ExAC), http://exac.broadinstitute.org
Exome Variant Server, http://evs.gs.washington.edu/
Exome Variant Server of the National Heart, Lung, and Blood Institute Grand Opportunity (NHLBI GO) Exome Sequencing Project (accessed February 2014), http://evs.gs.washington.edu/EVS/
Genome Analysis Toolkit (GATK), https://www.broadinstitute.org/gatk/
GenotypeTissue Expression (GTEx) Project, http://www.gtexportal.org
GnomAD, https://gnomad.broadinstitute.org/
GTEx, http://www.gtexportal.org/home/
Interactive bio-software, https://www.interactive-biosoftware.com/doc/alamut-visual
NCBI ClinVar database, https://www.ncbi.nlm.nih.gov/clinvar/
Online Mendelian Inheritance in Man (OMIM), http://omim.org/
Picard, http://broadinstitute.github.io/picard/
Primer-BLAST, https://www.ncbi.nlm.nih.gov/tools/primer-blast/
UCSC Genome Browser, http://genome.ucsc.edu/
UniProt database, https://www.uniprot.org/

References

1. Jahn, R., and Fasshauer, D. (2012). Molecular machines governing exocytosis of synaptic vesicles. Nature *490*, 201–207.

2. Hu, C., Ahmed, M., Melia, T.J., Söllner, T.H., Mayer, T., and Rothman, J.E. (2003). Fusion of cells by flipped SNAREs. Science *300*, 1745–1749.
3. Chen, Y.A., Scales, S.J., Patel, S.M., Doung, Y.C., and Scheller, R.H. (1999). SNARE complex formation is triggered by Ca^{2+} and drives membrane fusion. Cell *97*, 165–174.
4. Li, F., Kümmel, D., Coleman, J., Reinisch, K.M., Rothman, J.E., and Pincet, F. (2014). A half-zippered SNARE complex represents a functional intermediate in membrane fusion. J. Am. Chem. Soc. *136*, 3456–3464.
5. Gao, Y., Zorman, S., Gundersen, G., Xi, Z., Ma, L., Sirinakis, G., Rothman, J.E., and Zhang, Y. (2012). Single reconstituted neuronal SNARE complexes zipper in three distinct stages. Science *337*, 1340–1343.
6. Rothman, J.E., and Söllner, T.H. (1997). Throttles and dampers: controlling the engine of membrane fusion. Science *276*, 1212–1213.
7. Weber, T., Zemelman, B.V., McNew, J.A., Westermann, B., Gmachl, M., Parlati, F., Söllner, T.H., and Rothman, J.E. (1998). SNAREpins: minimal machinery for membrane fusion. Cell *92*, 759–772.
8. Melia, T.J., Weber, T., McNew, J.A., Fisher, L.E., Johnston, R.J., Parlati, F., Mahal, L.K., Sollner, T.H., and Rothman, J.E. (2002). Regulation of membrane fusion by the membrane-proximal coil of the t-SNARE during zippering of SNAREpins. J. Cell Biol. *158*, 929–940.
9. Brunger, A.T. (2005). Structure and function of SNARE and SNARE-interacting proteins. Q. Rev. Biophys. *38*, 1–47.
10. Rizo, J., and Rosenmund, C. (2008). Synaptic vesicle fusion. Nat. Struct. Mol. Biol. *15*, 665–674.
11. Hernandez, J.M., Stein, A., Behrmann, E., Riedel, D., Cypionka, A., Farsi, Z., Walla, P.J., Raunser, S., and Jahn, R. (2012). Membrane fusion intermediates via directional and full assembly of the SNARE complex. Science *336*, 1581–1584.
12. Shen, C., Rathore, S.S., Yu, H., Gulbranson, D.R., Hua, R., Zhang, C., Schoppa, N.E., and Shen, J. (2015). The trans-SNARE-regulating function of Munc18-1 is essential to synaptic exocytosis. Nat. Commun. *6*, 8852.
13. Schoch, S., Deák, F., Königstorfer, A., Mozhayeva, M., Sara, Y., Südhof, T.C., and Kavalali, E.T. (2001). SNARE function analyzed in synaptobrevin/VAMP knockout mice. Science *294*, 1117–1122.
14. Deák, F., Schoch, S., Liu, X., Südhof, T.C., and Kavalali, E.T. (2004). Synaptobrevin is essential for fast synaptic-vesicle endocytosis. Nat. Cell Biol. *6*, 1102–1108.
15. Mencacci, N.E., Kamsteeg, E.J., Nakashima, K., R'Bibo, L., Lynch, D.S., Balint, B., Willemsen, M.A., Adams, M.E., Wiethoff, S., Suzuki, K., et al. (2016). De Novo Mutations in PDE10A Cause Childhood-Onset Chorea with Bilateral Striatal Lesions. Am. J. Hum. Genet. *98*, 763–771.
16. Huang, Z., Sun, Y., Fan, Y., Wang, L., Liu, H., Gong, Z., Wang, J., Yan, H., Wang, Y., Hu, G., et al. (2018). Genetic Evaluation of 114 Chinese Short Stature Children in the Next Generation Era: a Single Center Study. Cell. Physiol. Biochem. *49*, 295–305.
17. Martín-Hernández, E., Rodríguez-García, M.E., Camacho, A., Matilla-Dueñas, A., García-Silva, M.T., Quijada-Fraile, P., Corral-Juan, M., Tejada-Palacios, P., de Las Heras, R.S., Arenas, J., et al. (2016). New ATP8A2 gene mutations associated with a novel syndrome: encephalopathy, intellectual disability, severe hypotonia, chorea and optic atrophy. Neurogenetics *17*, 259–263.
18. Salpietro, V., Perez-Dueñas, B., Nakashima, K., San Antonio-Arce, V., Manole, A., Efthymiou, S., Vandrovcova, J., Bettencourt, C., Mencacci, N.E., Klein, C., et al. (2018). A homozygous loss-of-function mutation in PDE2A associated to early-onset hereditary chorea. Mov. Disord. *33*, 482–488.
19. Sobreira, N., Schiettecatte, F., Boehm, C., Valle, D., and Hamosh, A. (2015). New tools for Mendelian disease gene identification: PhenoDB variant analysis module; and GeneMatcher, a web-based tool for linking investigators with an interest in the same gene. Hum. Mutat. *36*, 425–431.
20. Sobreira, N., Schiettecatte, F., Valle, D., and Hamosh, A. (2015). GeneMatcher: A matching tool for connecting investigators with an interest in the same gene. Hum. Mutat. *36*, 928–930.
21. Trabzuni, D., Ryten, M., Walker, R., Smith, C., Imran, S., Ramasamy, A., Weale, M.E., and Hardy, J. (2011). Quality control parameters on a large dataset of regionally dissected human control brains for whole genome expression studies. J. Neurochem. *119*, 275–282.
22. Weber, T., Parlati, F., McNew, J.A., Johnston, R.J., Westermann, B., Söllner, T.H., and Rothman, J.E. (2000). SNAREpins are functionally resistant to disruption by NSF and alphaSNAP. J. Cell Biol. *149*, 1063–1072.
23. Shen, J., Tareste, D.C., Paumet, F., Rothman, J.E., and Melia, T.J. (2007). Selective activation of cognate SNAREpins by Sec1/Munc18 proteins. Cell *128*, 183–195.
24. Südhof, T.C., and Rothman, J.E. (2009). Membrane fusion: Grappling with SNARE and SM proteins. Science *323*, 474–477.
25. Zhang, Y. (2017). Energetics, kinetics, and pathway of SNARE folding and assembly revealed by optical tweezers. Protein Sci. *26*, 1252–1265.
26. Sørensen, J.B., Matti, U., Wei, S.H., Nehring, R.B., Voets, T., Ashery, U., Binz, T., Neher, E., and Rettig, J. (2002). The SNARE protein SNAP-25 is linked to fast calcium triggering of exocytosis. Proc. Natl. Acad. Sci. USA *99*, 1627–1632.
27. Yin, J., Chen, W., Chao, E.S., Soriano, S., Wang, L., Wang, W., Cummock, S.E., Tao, H., Pang, K., Liu, Z., et al. (2018). Otud7a knockout mice recapitulate many neurological features of 15q13.3 microdeletion syndrome. Am. J. Hum. Genet. *102*, 296–308.
28. Reijnders, M.R.F., Miller, K.A., Alvi, M., Goos, J.A.C., Lees, M.M., de Burca, A., Henderson, A., Kraus, A., Mikat, B., de Vries, B.B.A., et al.; Deciphering Developmental Disorders Study (2018). De Novo and inherited loss-of-function variants in TLK2: Clinical and genotype-phenotype evaluation of a distinct neurodevelopmental disorder. Am. J. Hum. Genet. *102*, 1195–1203.
29. McTague, A., Howell, K.B., Cross, J.H., Kurian, M.A., and Scheffer, I.E. (2016). The genetic landscape of the epileptic encephalopathies of infancy and childhood. Lancet Neurol. *15*, 304–316.
30. Carecchio, M., and Mencacci, N.E. (2017). Emerging monogenic complex hyperkinetic disorders. Curr. Neurol. Neurosci. Rep. *17*, 97.
31. Shen, X.M., Selcen, D., Brengman, J., and Engel, A.G. (2014). Mutant SNAP25B causes myasthenia, cortical hyperexcitability, ataxia, and intellectual disability. Neurology *83*, 2247–2255.
32. Fukuda, H., Imagawa, E., Hamanaka, K., Fujita, A., Mitsuhashi, S., Miyatake, S., Mizuguchi, T., Takata, A., Miyake, N., Kramer, U., et al. (2018). A novel missense SNAP25b mutation in two affected siblings from an Israeli family showing seizures and cerebellar ataxia. J. Hum. Genet. *63*, 673–676.

33. Baker, K., Gordon, S.L., Melland, H., Bumbak, F., Scott, D.J., Jiang, T.J., Owen, D., Turner, B.J., Boyd, S.G., Rossi, M., et al.; Broad Center for Mendelian Genomics (2018). SYT1-associated neurodevelopmental disorder: A case series. Brain *141*, 2576–2591.
34. Baker, K., Gordon, S.L., Grozeva, D., van Kogelenberg, M., Roberts, N.Y., Pike, M., Blair, E., Hurles, M.E., Chong, W.K., Baldeweg, T., et al. (2015). Identification of a human synaptotagmin-1 mutation that perturbs synaptic vesicle cycling. J. Clin. Invest. *125*, 1670–1678.
35. Chen, D.H., Matsushita, M., Rainier, S., Meaney, B., Tisch, L., Feleke, A., Wolff, J., Lipe, H., Fink, J., Bird, T.D., and Raskind, W.H. (2005). Presence of alanine-to-valine substitutions in myofibrillogenesis regulator 1 in paroxysmal nonkinesigenic dyskinesia: confirmation in 2 kindreds. Arch. Neurol. *62*, 597–600.
36. Chen, W.J., Lin, Y., Xiong, Z.Q., Wei, W., Ni, W., Tan, G.H., Guo, S.L., He, J., Chen, Y.F., Zhang, Q.J., et al. (2011). Exome sequencing identifies truncating mutations in PRRT2 that cause paroxysmal kinesigenic dyskinesia. Nat. Genet. *43*, 1252–1255.
37. Lipstein, N., Verhoeven-Duif, N.M., Michelassi, F.E., Calloway, N., van Hasselt, P.M., Pienkowska, K., van Haaften, G., van Haelst, M.M., van Empelen, R., Cuppen, I., et al. (2017). Synaptic UNC13A protein variant causes increased neurotransmission and dyskinetic movement disorder. J. Clin. Invest. *127*, 1005–1018.
38. Saitsu, H., Kato, M., Mizuguchi, T., Hamada, K., Osaka, H., Tohyama, J., Uruno, K., Kumada, S., Nishiyama, K., Nishimura, A., et al. (2008). De novo mutations in the gene encoding STXBP1 (MUNC18-1) cause early infantile epileptic encephalopathy. Nat. Genet. *40*, 782–788.
39. Stamberger, H., Nikanorova, M., Willemsen, M.H., Accorsi, P., Angriman, M., Baier, H., Benkel-Herrenbrueck, I., Benoit, V., Budetta, M., Caliebe, A., et al. (2016). STXBP1 encephalopathy: A neurodevelopmental disorder including epilepsy. Neurology *86*, 954–962.
40. Liu, Y., Sugiura, Y., and Lin, W. (2011). The role of synaptobrevin1/VAMP1 in Ca2+-triggered neurotransmitter release at the mouse neuromuscular junction. J. Physiol. *589*, 1603–1618.
41. Bourassa, C.V., Meijer, I.A., Merner, N.D., Grewal, K.K., Stefanelli, M.G., Hodgkinson, K., Ives, E.J., Pryse-Phillips, W., Jog, M., Boycott, K., et al. (2012). VAMP1 mutation causes dominant hereditary spastic ataxia in Newfoundland families. Am. J. Hum. Genet. *91*, 548–552.
42. Salpietro, V., Lin, W., Delle Vedove, A., Storbeck, M., Liu, Y., Efthymiou, S., Manole, A., Wiethoff, S., Ye, Q., Saggar, A., et al.; SYNAPS Study Group (2017). Homozygous mutations in VAMP1 cause a presynaptic congenital myasthenic syndrome. Ann. Neurol. *81*, 597–603.
43. Shen, X.M., Scola, R.H., Lorenzoni, P.J., Kay, C.S., Werneck, L.C., Brengman, J., Selcen, D., and Engel, A.G. (2017). Novel synaptobrevin-1 mutation causes fatal congenital myasthenic syndrome. Ann. Clin. Transl. Neurol. *4*, 130–138.
44. Monies, D., Abouelhoda, M., AlSayed, M., Alhassnan, Z., Alotaibi, M., Kayyali, H., Al-Owain, M., Shah, A., Rahbeeni, Z., Al-Muhaizea, M.A., et al. (2017). The landscape of genetic diseases in Saudi Arabia based on the first 1000 diagnostic panels and exomes. Hum. Genet. *136*, 921–939.

The American Journal of Human Genetics, Volume *104*

Supplemental Data

Mutations in the Neuronal Vesicular SNARE *VAMP2* Affect Synaptic Membrane Fusion and Impair Human Neurodevelopment

Vincenzo Salpietro, Nancy T. Malintan, Isabel Llano-Rivas, Christine G. Spaeth, Stephanie Efthymiou, Pasquale Striano, Jana Vandrovcova, Maria C. Cutrupi, Roberto Chimenz, Emanuele David, Gabriella Di Rosa, Anna Marce-Grau, Miquel Raspall-Chaure, Elena Martin-Hernandez, Federico Zara, Carlo Minetti, Deciphering Developmental Disorders Study, SYNAPS Study Group, Oscar D. Bello, Rita De Zorzi, Sara Fortuna, Andrew Dauber, Mariam Alkhawaja, Tipu Sultan, Kshitij Mankad, Antonio Vitobello, Quentin Thomas, Frederic Tran Mau-Them, Laurence Faivre, Francisco Martinez-Azorin, Carlos E. Prada, Alfons Macaya, Dimitri M. Kullmann, James E. Rothman, Shyam S. Krishnakumar, and Henry Houlden

Supplemental Data

Subject recruitment and diagnosis

Case reports

Figures

Tables

Methods

Consortia and network involved in this study

References

Subject recruitment and diagnosis

For each affected individual, clinical data, brain imaging and EEG were reviewed by the clinicians (geneticists, neurologists and child neurologists, paediatricians) from the participating centres. Genomic DNA was extracted from the whole blood or saliva of the affected individuals and their parents after informed consent for DNA analysis was provided by the families. The study was approved by the ethics committee of the University College London (07/Q0512/26) and additional local ethics committees of the participating centres. Parents of the affected individuals (and when available unaffected siblings) were recruited for segregation analysis, which was carried out using Sanger sequencing. The individuals diagnosed with neurodevelopmental impairment including intellectual disability (ID), developmental delay (DD), autism spectrum disorder (ASD), Rett syndrome (RTT)- like stereotypies, and epileptic encephalopathy (EE) were recruited in the different Institutions participating to the study. Based on the International League against epilepsy (ILAE) classification, an EE was defined in the patients as refractory seizures and cognitive slowing or regression associated with frequent, ongoing epileptiform activity.[1]

Based on the RTT diagnostic criteria,[2] the affected individuals from this cohort having at least 1/4 main RTT criteria and ≥4 supportive criteria have been diagnosed with RTT-like features, as previously reported. [3-5]

ID was defined based on the presence of significant deficits conceptual, social and/or practical skills associated to significant deficits in adaptive behavior.[6]

Detailed epilepsy and medical histories were obtained as well as the results of investigations including EEG and MRI studies. The 4 individuals carrying de novo *VAMP2* intragenic variants reported in the present study were recruited from different research groups and consortia in the UK and internationally. Patient 1 was followed up at University Hospital "Gaetano Martino" (Messina, Italy) and was genetically investigated by trio- whole exome sequencing (WES) as part of the SYNAPS Study Group (http://neurogenetics.co.uk/synaptopathies-synaps/) initiative. Patient 2 was recruited and studied by trios WES at Cincinnati Children's Hospital Medical Center (Ohio, USA). Patient 3 was recruited at the Genetic Unit in Cruces University Hospital (Bilbao, Spain) and investigated by trio WES at the Instituto de Investigaciòn Hospital 12 de Octubre (i+12) (Madrid, Spain). Patient 4 was followed up at University Hospital Vall d'Hebron (Barcelona, Spain) and was identified by reviewing the SYNAPS Study Group exome dataset during the replication cohort screening phase of this study. Patient 5 was followed up at University Hospital of Dijon (Bourgogne, France). Routine clinical genetic and metabolic screenings performed during initial workup was negative in each case, which warranted further investigation on a research basis. All families gave written informed consent for inclusion in the study, including for the publication of photographs and videos.

Supplemental case reports

Patient 1

Patient 1 is a 3-year-old Italian girl born at term after an uneventful pregnancy. Familial history was negative for neurological disorders. Her growth parameters, including occipito-frontal circumference (OFC) at birth were within normal range. She has a history of hypotonia and developmental delay since the first month of life (Video S1). She became able to hold her neck at 10 months of age and she acquired the ability of sitting autonomously only at the age of 2 years and 6 months; at the present age of 3 years she still is unable to walk. All other major developmental milestones, were delayed, and her current cognitive and social skills are severely impaired. Since the first months of life, impaired visual fixation was evident, with only occasional and brief episodes of eye contact (Video S2). During the first year of her life, a virtual absence of purposeful hand movements was also noticed (Video S2). At around 6 months of age a complex hyperkinetic movement disorder became evident. This consists in distal choreic-predominant movements (mainly involving upper and lower limbs) associated to dystonic postures of the neck and the trunk. She also exhibit since the first year of life complex motor stereotypies which include arm flapping, RTT-like hand-predominant stereotypies including hand-wringing, repeated clapping, rocking and raspberry blowing. She also present since infancy frequent automatisms (hand to mouth) and frequent episodes of staring (Videos S1-S2). Brain MRI scan performed at the age of 2 years showed some generalised delay in the maturation of myelin, with a reduced volume of the cerebral white matter posteriorly; also, the optic nerves and chiasm were found hypoplastic. Because of the poor visual contact she underwent funduscopic examination which was reported as normal and she was diagnosed with cortical visual impairment. Until the present age of 3 years, she did not presented clinically with epileptic seizures, but ictal EEG recording performed at the age of 15 months showed high- voltage delta activity with interposed sharp wave-slow wave complexes (predominant over the right central and posterior brain regions). She was never put under anti-epileptic drugs (AEDs). She has not showed developmental regression. Currently, she has severe speech impairment (aphasia). Trio WES identified a de novo missense variant in *VAMP2* [NM_014232: c.223T>C (p.Ser75Pro)].

Patient 2

Patient 2 is a 10-year-old Caucasian boy born at term by normal delivery. At birth, his growth parameters were normal. Since the first month of life, he started experiencing multiple episodes per day of focal seizures, with ictal EEG recording showing fast rhythmic activity followed by sharp wave-slow wave complexes over the right occipital areas. Delay in his developmental milestones, as well difficulties with language, social interaction and behavior were noticed since his first infancy. He is non-verbal and does not follow verbal commands. He has severe self-injurious behaviours. He exhibited poor visual fixation since the first months of life and was lately diagnosed with cortical visual impairment (although he can respond to bright colours). During his childhood, frequency and severity of seizures increased with up to 100 episodes per day. At 6 months of age he underwent craniotomy for grid placement and had a right posterior circulation stroke affecting the thalamic and cortical areas. At 18 months of age he had a right temporal lobectomy and subsequent resection of areas of focal cortical dysplasia posterior to the temporal lobe. Around the age of 2 years the Patient developed a hyperkinetic movement disorder with severe chorea associated to dystonic

postures (Video S3). At the age of 6 years he underwent deep brain stimulation which mildly improved the severity of his movement disorder. In addition to neurodevelopmental impairment, epilepsy and abnormal movements, he presented complex motor stereotypies, including hand stereotypies (e.g., wringing), rocking, oral automatisms (hand to mouth), and virtual absence of purposeful hand movements (Video S3). He also present behaviour abnormalities with episodes of self-injuries, head banging, screaming spells, and exaggerated startle response. Because of his behaviour, he was put under risperidone and escitalopram and paliperidone. At his current age of 10 years, he present motor impairment (he can sit independently but he has backward falling and cannot stand nor walk without assistance), and his seizures are now significantly decreased in frequency and severity with only 1-2 episodes per month. Brain MRI, microarray and metabolic studies were performed and resulted as normal. He performed array-CGH that was normal. Trio-WES identified a variant in *VAMP2* [NM_014232: c.233>C (p.Glu78Ala)] as the only rare *de-novo* variant in the child.

Patient 3

He is a 13-year-old Spanish boy born at term by normal delivery. At birth, his growth parameters including OFC were normal. At 20 days of life episodes of apneas were detected, with the exclusion of infectious, metabolic or cardiologic causes. At the age of 5 months the Patient was evaluated because of a delay in his developmental milestones. At that time, the child was not yet able to hold his neck; in addition, axial hypotonia, poor visual fixation and stereotyped hand movements (e.g., clapping, washing) were noticed. He presented a severe delay in motor milestones and never attained the ability to walk (and became wheelchair-dependent). The Patient exhibited since the first months of his life poor visual fixation since the first months of life and was lately diagnosed with cortical visual impairment. During his infancy he developed a hyperkinetic movement disorder characterized by the combination of choreic movements (predominant in the upper limbs). At the age of 12 months he presented with episodes consisting in brief lapse of consciousness with staring and upward rolling of the eyes; these episodes were associated to generalized paroxysm and lentified below activity on EEG. He was put under valproic acid and then vigabatrine, but episodes changed becoming generalized spasms with disorganization and paroxysms on EEG. At the present age of 13 years he is under clonazepam treatment and chetogenic diet and does not present anymore epileptic episodes. He still present flapping, stereotyped hand movements, and choreic hyperkinetic movements (Video S4). He is non-verbal and present difficulties in social interaction. He underwent multiple metabolic and genetic test including array CGH and Sanger sequencing of different genes (*MECP2, CDKL5, FOXG1*) which resulted normal. Trio-WES identified a variant in *VAMP2* [NM_014232: c.230T>C (p.Phe77Ser)] that was confirmed *de-novo* by Sanger sequencing.

Patient 4

He is a 14-year-old Caucasian boy who was born at term after an uneventful pregnancy and a normal delivery. There is no history of neurological disorders in the family. Both parents and 2 younger sibs are healthy. Global developmental delay was already suspected at 6 months of age. Further milestone achievements confirmed the patient's neurodevelopmental disorder including mild hypotonia, gross motor delay (he attained independent ambulation after 3 age years) and some degrees of clumsiness and incoordination (Video S5). Severe receptive and expressive language disorder was also evident since infancy and, at his present age of 14 years, he is able to understand simple verbal commands and use gestural expressive communication and a 5- 10 simple words. His visual contact and fixation were present since early infancy, but he presented impaired social communication, abnormal behaviour with restricted interests and prominent hand stereotypies (Video S5) including Rett syndrome (RTT) – like features (e.g., hand wringing, washing, clapping) that

led to the diagnosis of Autistic Spectrum Disorder (ASD). Currently, clinical examination does not show definite dysmorphic features; there have not been signs of developmental regression and OFC is normal. No motor or sensory anomalies are apparent, and he is able to walk without assistance. Brain MRI, visual evoked potentials, metabolic screening, Multiplex Ligation-dependent Probe Amplification (MLPA) for sub telomeric deletions, array-CGH and panel sequencing of 55 genes linked to non-syndromic intellectual disability were all normal. He developed infrequent staring episodes with eyelid myoclonus at 5 years of age. Several EEG recordings showed both generalized and multifocal interictal epileptiform discharges and sodium valproate was started. He remained seizure-free until the age of 11 years when he suffered a single episode consistent with non-convulsive status epilepticus, described as with impaired consciousness and subtle clonic movements involving the right arm during which the patient could maintain stance or even walk around. Valproic acid dose was adjusted and he has remained seizure free ever since and with normal follow-up EEGs. WES identified a de novo single amino acid deletion at position 43 [NM_014232: c.128_130delTGG (p.Val43del)].

Patient 5

Patient 5 is a 3-year-old French girl born at term after an uneventful pregnancy. Familial history was negative for neurological or genetic disorders. Her growth parameters, including OFC at birth were within normal range. She has a history of hypotonia since birth and developmental delay was evident since the first month of life. She became able to hold her neck at the age of 9 months and to sit with support at the age of 12 months. All other major developmental milestones were delayed, and her current cognitive and social skills are severely impaired. His visual contact and fixation were present since early infancy, but she presented mild convergent strabismus (right > left), hyperopia and astigmatism. She has impaired verbal and non-verbal communication, abnormal behaviour and stereotypies including arms flapping and hand washing and clapping, that led to the diagnosis of Autistic Spectrum Disorder (ASD). She never presented seizures and her EEG was unrevealing. Currently, clinical examination does not show definite dysmorphic features. She has a wide nasal bridge, anteverted nares, thick lips, full cheeks, stellar iris, and excessive skin in the neck. At the present age of 3 years, she is able to pronounce 4-5 words and became capable of walking when parents give her one hand. She currently benefits from speech therapy, psychomotricity and physiotherapy. She has a normal height and weight growth. Brain MRI, visual evoked potentials, metabolic screening, Multiplex Ligation-dependent Probe Amplification (MLPA) for sub-telomeric deletions and array-CGH were all normal. WES identified a de novo single amino acid deletion at position 45 [NM_014232: c.135_137delCAT (p.Ile45del)].

Supplemental Figures

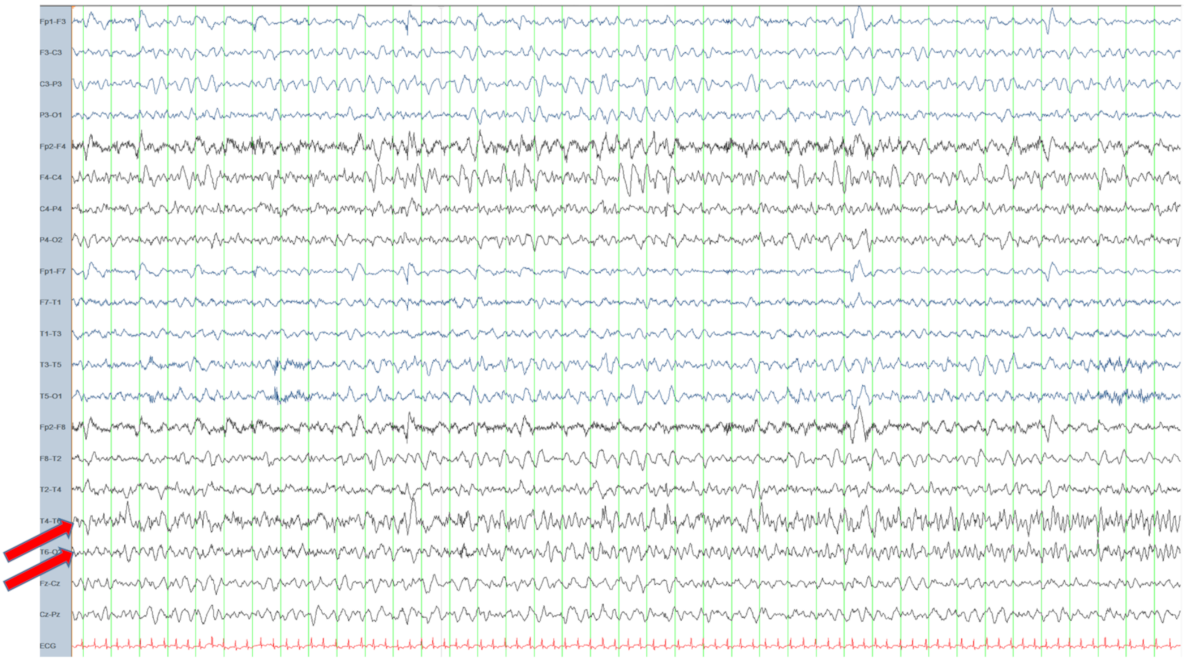

Supplementary Figure 1. Ictal EEG showing a focal seizure.

Ictal EEG recording from Patient 2 (E78A) at the age of 8 months showing a focal seizure characterized by fast rhythmic activity intermixed with high-voltage polymorphic theta activity and sharp waves over the right anterior temporal brain areas. The EKG trace shows concomitant sinusal tachycardia.

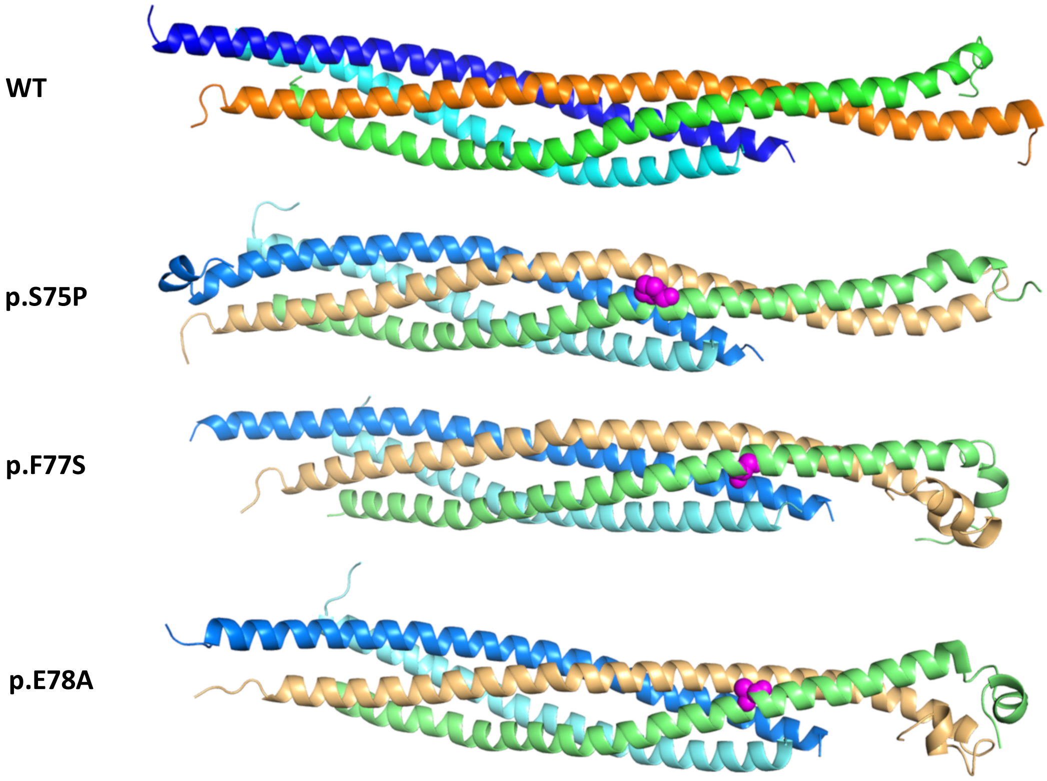

Supplementary Figure 2. Molecular Modelling of identified *VAMP2* variants.

Rearrangement of the VAMP2 ectodomain/Stx1a/Snap25 complex after 100 ns of molecular dynamics simulation for the wild type VAMP 2 protein and its mutants. VAMP2 ectodomain is depicted in green for Wild Type, light green for mutants; Syntaxin-1A is depicted in orange for Wild Type, light orange for mutants; Synaptosomal-associated protein 25 chains are represented in blue and cyan for Wild Type, marine and aquamarine for mutants. Point of mutation is represented with magenta spheres.

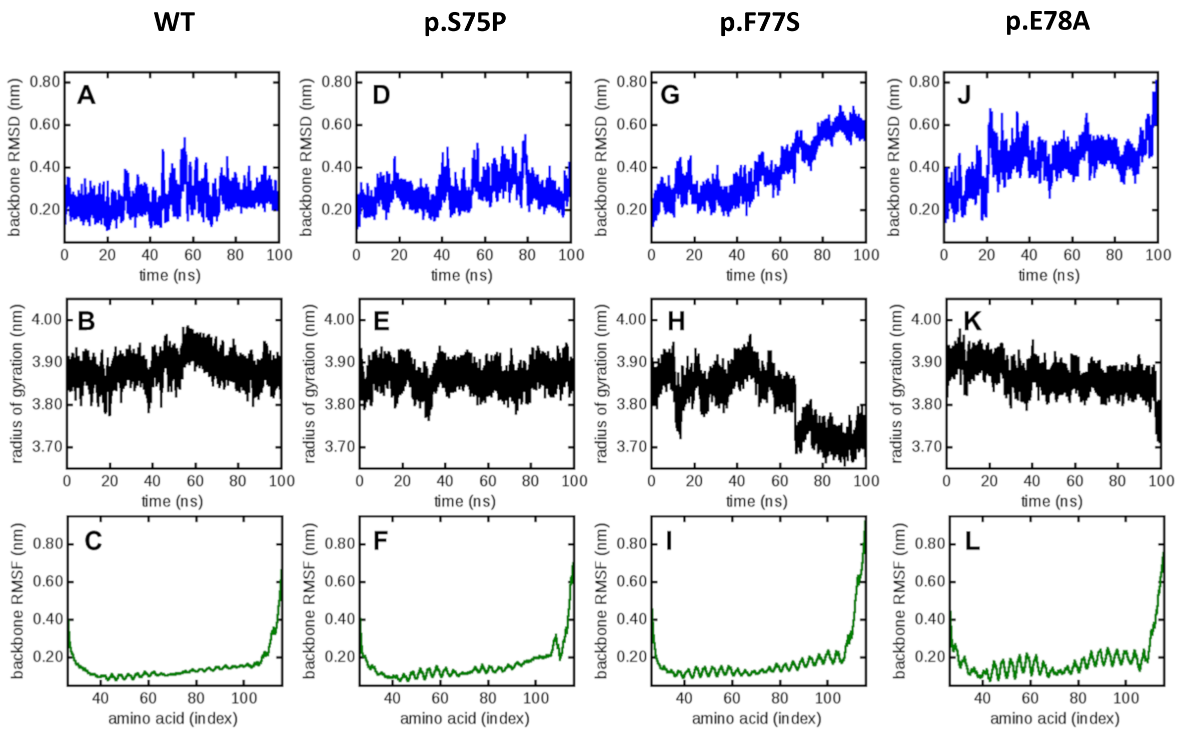

Supplementary Figure 3. Backbone Root Mean Square Deviation of identified *VAMP2* variants.

Backbone Root Mean Square Deviation (RMSD, blue), backbone radius of gyration (black), and backbone RMSF (green) for (A-C) VAMP2 wilde type and its mutants (D-F) S75P, (G-I) F77S, and (J-L) E78A.

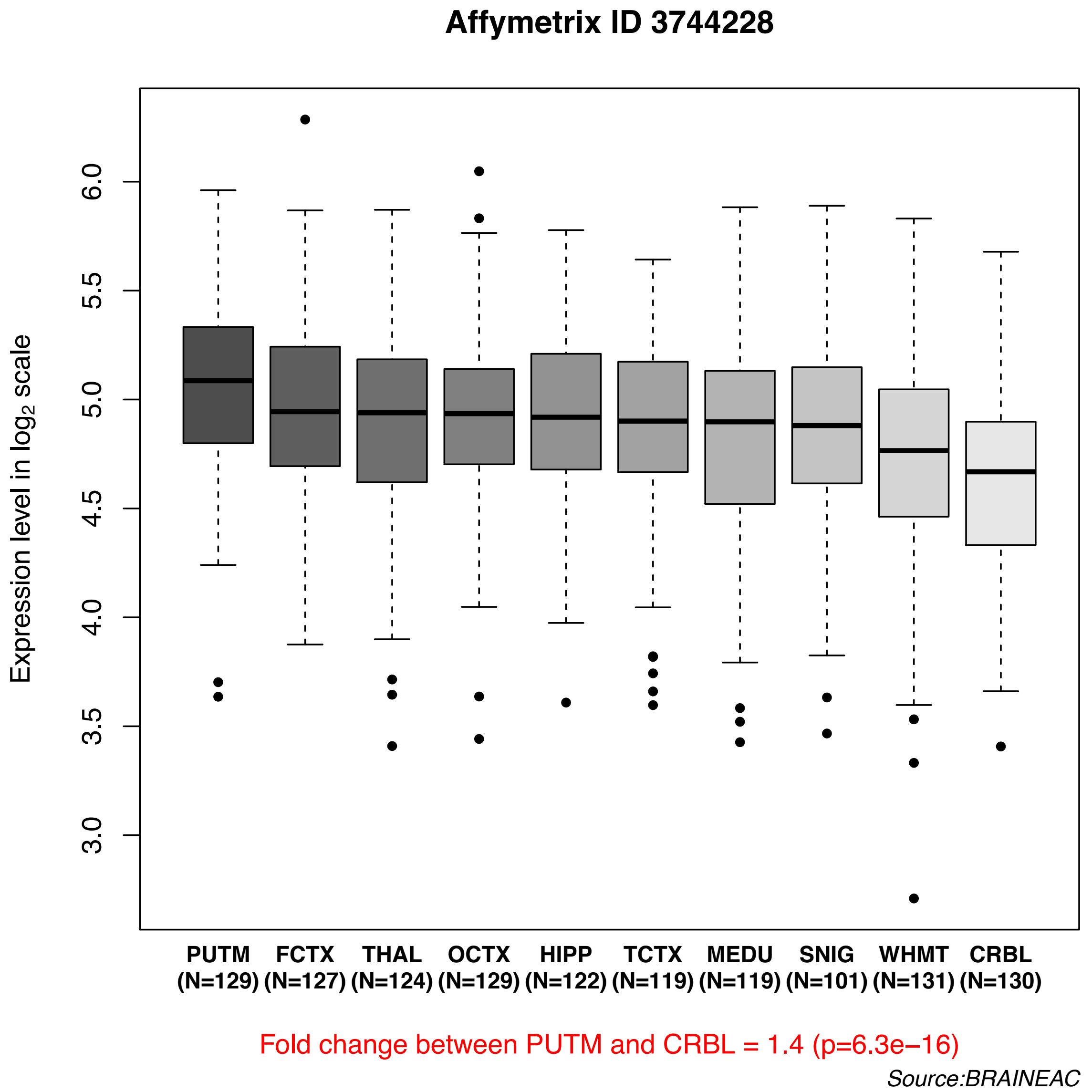

Supplementary Figure 4. Regional brain expression of *VAMP2*

Brain expression values of *VAMP2* show higher expression levels in the striatum and also other CNS regions, especially FCTX (frontal cortex), THAL (thalamus) and OCTX (occipital cortex), and less in TCTX (temporal cortex), HIPP (hippocampus), WHMT (white matter), SNIG (substantia nigra), MEDU (medulla), and CRBL (cerebellar cortex).

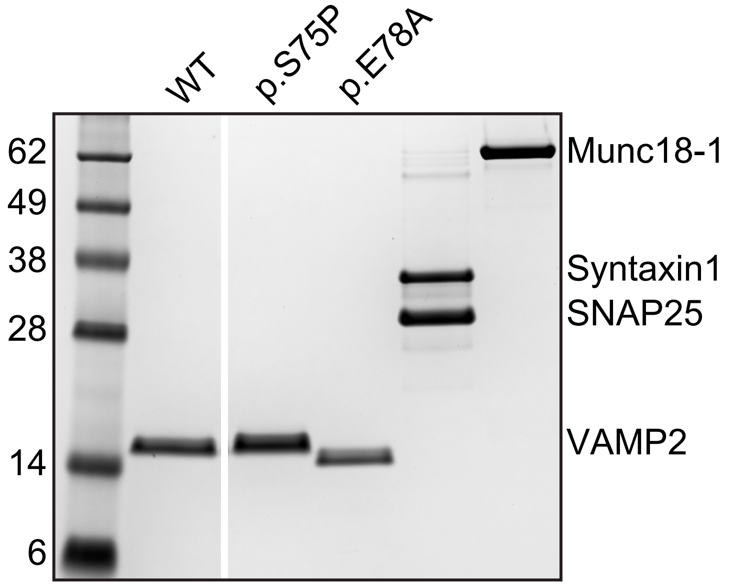

Supplementary Figure 5. Disease variants VAMP2 proteins expression and purification.

The SDS-PAGE and Coomassie-stained gel image showing the disease variants VAMP2 proteins (A67P, S75P and E78A) expressed and purified to similar integrity to the WT protein. The t-SNARE (Syntaxin1 and SNAP25) and Munc18-1 proteins used in the experiments were also analysed on gel. All proteins were resolved to their corresponding molecular size, indicating reasonable protein quality.

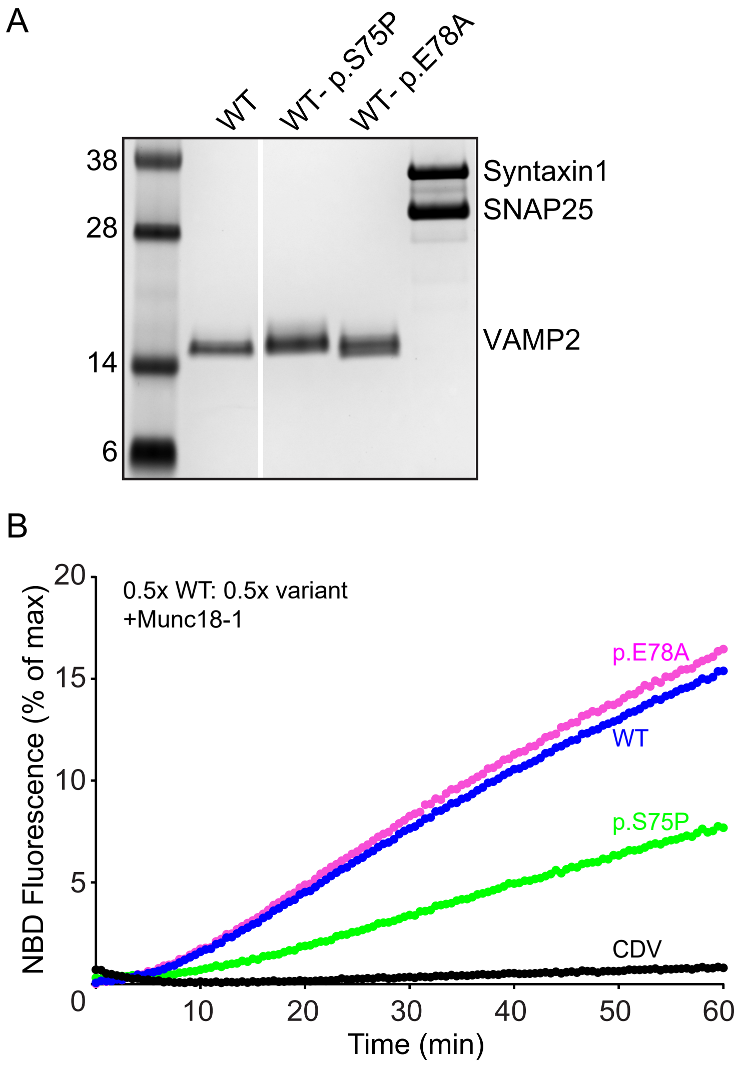

Supplementary Figure 6. Disease variants VAMP2 proteins cause loss-of-function in SNARE-mediated liposome fusion assay.

(A) The SDS-PAGE and Coomassie-stained gel image showing donor v-liposomes reconstituted with VAMP2 wildtype (WT), and mixture of WT and disease variants VAMP2 (A67P, S75P, E78A). Gel also shows t-SNARE (Syntaxin1 and SNAP25) acceptor t-liposomes. (B), Line graphs showing the average (with Munc18-1) increase in NBD fluorescence due to fusion between WT or WT-disease variants VAMP2 (A67P, S75P, E78A) v- and t-SNARE liposomes. Liposome fusion reaction in the presence of CDV was used as negative control.

Supplemental Table 1. De novo *VAMP2* variants identified in this study

Patient	Genomic coordinates (GRCh37/hg19)	Variant	dbSNP138	1000G	ESP6500	ExAC	gnomAD	SIFT	PolyPhen	Mutation Taster	CADD_PHRED	GERP++
1	17:8064985-8064985	NM_014232: c.223T>C; p.Ser75Pro	-	-	-	-	-	D(0)	D(0.925)	D(1)	27.3	5.26
2	17:8064978-8064978	NM_014232: c.230T>C; p.Phe77Ser	-	-	-	-	-	D(0)	D(0.997)	D(1)	27.7	5.26
3	17:8064975-8064975	NM_014232: c.233A>C; p.Glu78Ala	-	-	-	-	-	D(0)	D(0.56)	D(1)	25.9	5.26
4	17:8065077-8065080	NM_014232: c.128_130delTG G ; p.Val43del	-	-	-	-	-	-	-	-	-	-
5	17:8065071-8065073	NM_014232: c.135_137delCAT; p.Ile45del	-	-	-	-	-	-	-	-	-	-

Supplemental Methods

Genetic analyses

All research centres involved in this study followed a trio-based WES approach to identify the de novo *VAMP2* variants as the cause of the neurodevelopmental phenotypes of the patients. Most of the methods used by the centres were detailed previously. The SYNAPS Study Group (neurogenetics.co.uk/synaptopathies-synaps) analysed approximately 335 trios of children with neurodevelopmental impairment (as part of a larger cohort of ~4,750 individuals affected with early-onset neurological disorders). Following their respective analysis pipelines, participating centres generated a list of candidate variants filtered against public database variants and according to modes of inheritance. All variants reported in the present study were determined independently by participating centres. Connecting the different contributing centres was facilitated by the web-based tools.[7]

WES data analysis and variant calling

Libraries were prepared from parent and patient DNA, and exomes were captured and sequenced by trio-WES on Illumina sequencers. Raw data were processed and filtered with established pipelines at the academic laboratories involved in the study.[8-12] In Patient 2, 3 and 5, the sequence reads were aligned against the human reference genome (GRCh37/hg19) using Burrows-Wheeler Aligner (BWA) in order to obtain candidate variants. Single-nucleotide variants (SNVs) and short insertion or deletion variants (indels) were identified using Haplotype Caller of GATK (v3.3.0) according to the Best Practices for variant analysis. In Patients 1 and 4, variant (single nucleotide variants and indels) calling and filtering was performed using the Genome Analysis Tool Kit (GATK; see URLs). In all cases, variants that did not adhere to the following criteria were excluded from further analysis: (1) allele balance of >0.70, (2) QUAL of <20, (3) QD of <5 and (4) coverage of <20×.

Variants were annotated and the Exome variant server ESP6500 (evs.gs.washington.edu) was used to assess variant frequency in the control population. In the index case trio WES (Patient 1) the average sequencing depth of the on-target regions was 78.2 reads per nucleotide, with 96.8% of the regions covered at least 20X.

In all five trios studied, only exonic and donor/acceptor splicing variants were considered. Synonymous variants were also excluded. Priority was given to rare variants (<1% in public databases, including 1000 Genomes Project, NHLBI Exome Variant Server, Complete Genomics 69, and ExAC with a GERP++ score > 2). PCR and Sanger sequencing were conducted according to standard methods (detailed conditions of the primers used, and sequencing methods are available upon request). The filtered variants were confirmed by the conventional Sanger sequencing according to standard methods (available upon request).

Variants filtering and identification of *VAMP2* variants

Following their respective analysis pipelines, participating centres generated a list of candidate variants filtered against public database variants and according to modes of inheritance. All participating centres prioritized autosomal recessive and dominant de novo mutations in the analysis and annotated variants using the Variant Effect Predictor (Ensembl release 75) based on Sequence Ontology nomenclature: missense variant, initiator codon variant, splice donor or acceptor variant, frameshift variant, stop lost, stop gained, in frame insertion or deletion. To exclude likely benign amino acid changes, missense variants were further considered only if predicted damaging by at least 3 out of 5 in-silico methods we used (PolyPhen-2, SIFT, Mutation Taster, Condel and CADD, see URLs). Variants that were not present in both the mother and the father of the probands were considered de novo. In recessive filtering, homozygous, hemizygous or compound heterozygous variants were included. Variants present in >1% of our internal exome dataset at the UCL Institute of Neurology (containing ~ 5000 exomes from individuals affected with a range of neurological disorders) were excluded. Exome data were analyzed for variants in genes linked before to NDDs, epilepsy and RTT-like presentations, and for variants in possible new genes. Genes involved in EE and RTT-like presentations were retrieved from the literature.[4,5] Based on values from the ExAC database (containing 60,706 individuals), we also prioritized variants in genes with high probability of being LoF intolerant (i.e., ExAC pLI >0.9) and highly constrained for missense variations (Z-score >2). In our analysis, we also prioritized variants affecting domains important to genes function (UniProt database) and variants in genes implicated in brain development and function (literature) and variants in genes predominantly expressed in the central nervous system (GTEx database). In the case of new candidates, variants in genes whose homologues or functionally correlated genes are already established causing similar neurological disorders were also prioritized. In Patient 1 who was analyzed in the discovery phase of this study, the only de novo variant identified by WES and confirmed by Sanger sequencing was in *VAMP2* [NM_014232: c.223T>C (p.Ser75Pro)]. Similarly to the index case (Patient 1), also in other research and diagnostic laboratories the identified de novo variants in *VAMP2* [NM_014232: c.230T>C (p.Phe77Ser), c.233A>C (p.Glu78Ala), c.128_130delTGG (p.Val43del), c.135_137delCAT (p.Ile45del)] were prioritized (Supplemental Table 1).

VAMP2 mutations thus emerged in each participating centre as the most likely explanation for the individuals disease pathogenesis, as supported by crucial role of the gene in synaptic transmission and the high conservation and biological importance of the affected residues within the C- terminus of the v-SNARE domain. Variants were submitted to Leiden Open (source) Variation Database (LOVD; www.lovd.nl). All the de novo *VAMP2* variants were found with trio WES and confirmed in all cases by trio Sanger sequencing. All variants in were annotated with the transcript NM_014232. For trio Sanger, the following sets of intronic primers designed by Primer3 were used for both PCR and sequencing: Forward (5'- CTGTGTGTCCTTGGCATGTT- 3') Reverse (5'-ATACCCCATTCACCCACCTG -3'). PCR products were amplified using 50 ng of DNA, with standard FastStart PCR reagents (Roche), on an ABI Veriti Thermal Cycler (Applied Biosystems). PCR products were purified using Exo-SAP (Exonuclease I and Shrimp Alkaline Phosphatase; incubated at 37°C for 15 minutes followed by inactivation by heating to 80°C for 15 minutes) and sequencing PCR was performed bi-directionally using BigDye Terminator Ready Reaction Mix kit version 3.1 (Applied Biosystems) and analysed on an ABI 3730xl capillary sequencer.

Replication cohort screening

As part of our replication cohort analysis we then searched for variants in the *VAMP2* gene within genetic datasets from undiagnosed patients recruited in the SYNAPS Study Group which contains approximately 335 trios of children with neurodevelopmental impairment as part of a larger cohort of ~4,750 individuals affected with early-onset neurological disorders. This led to the identification of Patient 4 carrying a de novo single amino acid deletion at position 43 [NM_014232: c.128_130delTGG (p.Val43del)]. Another de novo single amino acid deletion at position 45 [NM_014232: c.135_137delCAT (p.Ile45del)] was identified by comparing results with international colleagues through the Web-based tool GeneMatcher.[13-15]

Functional analyses

Recombinant proteins expression and purification

All recombinant protein constructs used in the experiments were expressed in bacteria BL21 (DE3) E. coli cell lines. The pTW34-trans-SNARE construct co-encoding the 6-histidine-tagged mouse SNAP25 and rat Syntaxin1a (pTW34-t-SNARE) was expressed and purified as previously described.[16, 17] Upon overnight affinity pulldown on the Ni-NTA beads at 4 °C, t-SNARE proteins were eluted into an n-Octyl-D-glucopyranoside (OG) buffer A (25 mM HEPES-OH pH7.4, 400 mM KCl, 10 % glycerol, 1 % OG and 1 mM DTT) containing 300 mM imidazole pH7.4. The pET-6-histidines-SUMO-tagged constructs encoding the rat Munc18-1, full-length wildtype (WT) mouse VAMP2 and cytoplasmic domain of VAMP2 (CDV) were described.[18] Site-directed mutagenesis using the WT VAMP2 as template was performed to generate the disease variants VAMP2 containing amino acid substitution at Serine75 to Proline (S75P) and Glutame78 to Alanine (E78A). The WT and variants VAMP2 proteins were then expressed and purified as described previously (Shen J et al; 2007). The His6-SUMO proteins were incubated for 3-hour at 4 °C on the Ni-NTA affinity beads, followed by washing using OG buffer for VAMP2 and buffer A for Munc18-1. The SUMO-tag was removed by overnight cleaving on beads using 50 ul of SUMO-protease at 4 °C. The concentration of SUMO-protease used for tag-cleaving is typically at 4-7 mg/ml. The VAMP2 proteins were eluted into OG buffer and Munc18-1 was eluted into buffer A without OG. Purified proteins were evaluated on SDS-PAGE and Coomassie-stained. Protein concentrations were determined by colourimetry reaction using Bradford dye.[19]

Proteoliposome reconstitution for lipid mixing assay

The purified SNARE proteins were reconstituted into lipid vesicles (Avanti Polar Lipids) by detergent dilution and dialysis. Proteoliposomes were isolated by floatation using Nycodenz density gradient as previously described.[17] The t-SNARE protein was reconstituted into palmitoyl-2-oleoyl phosphatidylcholine (POPC): 1,2 dioleoyl phosphatidylserine (DOPS) at 85:15 mol% liposomes at protein to lipid ratio of 1:400. The VAMP2 proteins used in the reconstitution were into POPC, DOPS and the fluorescent probes NBD-PE and rhodamine- PE (1.5 mol% each) at protein to lipid ratio of 1:150, mimicking the reported VAMP2 densities on the synaptic vesicles.[20, 21] Proteoliposomes containing mixture of WT VAMP2 and mutant proteins were reconstituted at protein to lipid ratio of 1:300 each.

Liposome fusion assay

The lipid-mixing assay utilised the Föster resonance energy transfer (FRET) principle.[22, 23] In this assay, v- SNARE liposomes contained PE-lipids labelled with fluorophores Nitro-2-1, 3 benzoxadiazol-4yl- phosphatidylethanolamine (NBD-PE) and lissamine rhodamine B (RHO). Within a closed distance in a liposome, NBD fluorescence is quenched in the presence of rhodamine. Upon fusion of v-SNARE liposomes with the unlabeled t-SNARE liposomes, the spatial interaction between NBD and rhodamine molecules increased. Dequenching of NBD, concomitant with increase in NBD fluorescence provide a measure of liposome fusion.[22] Liposome fusion assay was performed by mixing 5 µl of the donor liposome (VAMP2) with 45 µl of the acceptor liposome (t-SNAREs). Liposomes mixes were pre-incubated in the presence or absence of Munc18-1 on ice for 3-hour to allow for trans-SNARE complex assembly. Fusion of liposomes was monitored by the change in NBD

fluorescence at 538 nm using a fluorescent microplate reader. After 60 min, 10 µl of 2.5 % w/v n-dodecyl-β-maltoside was added to lyse all vesicles to estimate the maximum NBD fluorescence.[13]

Molecular modelling and dynamic stimulations

We build a model of each mutant ectodomain by homology modelling with the humanised wild type (WT) as a template. We follow the behaviour of the WT and each mutant along time by means of full atom molecular dynamics simulations in water solvent. The soluble WT VAMP2 fragment was built from the first complex of the asymmetric units of structure PDB ID 3HD7 representing the neuronal SNARE complex from Rat.[24] We humanized the complex by mutating two residues (V278I, V283I) in Chain B (protein Stx1a). The reported VAMP2 chain (Chain A) does not include the mutation (V8A) from Rat to Human and therefore it was left as it is. The same applied to the other two chains (chain C e D, SNAP25). The humanised complex was then placed in a cubic box, and minimized. All the other mutants were constructed from this minimised humanised complex. All the complexes containing the mutants, including the WT, were then placed in a triclinic box and minimised. A water layer of 0.8 nm and Na^+ ions to neutralize the system were added, and a second minimization was performed. In all cases we used AMBER99SB-ILDN force field and Simple Point Charge water. On all systems we performed NVP and NPT equilibrations for 100 ps, followed by 100 ns NPT production run at 300 K. The temperature was controlled with a modified Berendsen thermostat[25] the pressure with an isotropic Parrinello-Rahman at 1 bar. The iteration time step was set to 2 fs with the Verlet integrator and LINCS constraint.[26] We used periodic boundary conditions. Configurations were sampled every 10ps. All the simulations and their analysis were run as implemented in the GROMACS.[27] During the simulations, the WT and S75P seem to reach a stationary state while major rearrangements are still observed for F77S and E78A at end-simulation as emerges in their backbone root mean squared deviation (RMSD, Supplementary Figure 3 A, D, G, J) and radius of gyration (RMSD, Supplementary Figure 3B, E, H, K). In all cases the most mobile portion of the chain is that close to the C-term as seen in their root mean squared fluctuation (RMSF, Supplementary Figure 3 C, F, I, L). The RMSF further indicates that the in all cases the mutations enhance the mobility of the backbone, an effect particularly evident for E78A (Supplementary Figure 3L).

Consortia and networks involved in this study

The Deciphering Developmental Disorders (DDD) Study (http://www.ddduk.org/)

Central DDD Team

Wellcome Trust Sanger Institute, Wellcome Trust Genome Campus, Hinxton, Cambridge, CB10 1SA, UK & The Ethox Centre, Nuffield Department of Population Health, University of Oxford, Old Road Campus, Oxford, OX3 7LF, UK

DDD Management Team (*Principal Investigator)

Jeffrey C. Barrett, Nigel P. Carter, Helen V. Firth, David R. Fitzpatrick, Matthew E. Hurles*, Michael Parker, Caroline F. Wright

DDD Laboratory Team

Kirsty Ambridge, Daniel M. Barrett, Tanya Bayzetinova, Susan Gribble, Netravathi Krishnappa, Laura E. Mason, Elena Prigmore, Diana Rajan

DDD Model Organisms

Eve L. Coomber, Sebastian S. Gerety

DDD Informatics Team

Stephen Clayton, Tomas W. Fitzgerald, Philip Jones, Ray Miller, Adrian R. Tivey DDD Analysis Team Nadia Akawi, Saeed Al-Turki, Jeffrey C. Barrett, Tomas W. Fitzgerald, Matthew E. Hurles, Wendy D. Jones, Daniel King, Margriet van Kogelenberg, Jeremy McRae, Katherine I. Morley, Vijaya Parthiban, Alejandro Sifrim

DDD Ethics, Social Science and Policy Team

Anna Middleton, Michael Parker, Caroline F. Wright

Wellcome Trust Sanger Institute Staff

Wellcome Trust Sanger Institute, Wellcome Trust Genome Campus, Hinxton, Cambridge, CB10 1SA, UK

DECIPHER Team:Paul Bevan, Eugene Bragin, G. Jawahar Swaminathan

WTSI Pipelines Staff (sample QC, genotyping, pulldown, sequencing, informatics)

Rob Andrews, John Burton, Suzannah J. Bumpstead, Sarah Edkins, Peter Ellis, Emma Gray, David Jones, Carol Scott, Douglas Simpkin, Danielle Walker, Sara Widaa

WTSI FISH Team

Ruby Banerjee, Beiyuan Fu, Sandra Louzada Gomes Pereira, Fentang Yang

UK NHS Regional Genetic Services (* local Principal Investigator)

Aberdeen (North of Scotland Regional Genetics Service, NHS Grampian, Department of Medical Genetics Medical School, Foresterhill, Aberdeen, AB25 2ZD, UK)

Recruiting Consultant Clinical Geneticists: John Dean*, Ruth McGowan, Alison Ross

Research Nurse/ Genetic Counsellors: Mariella D'Alessandro Diagnostic Laboratory Scientists: Paul Batstone, Shalaka Samant

Belfast (Northern Ireland Regional Genetics Centre, Belfast Health and Social Care Trust, Belfast City Hospital, Lisburn Road, Belfast, BT9 7AB, UK)

Recruiting Consultant Clinical Geneticists: Tabib Dabir, Deirdre Donnelly, Alex Magee, Vivienne McConnell, Shane McKee*, Fiona Stewart

Research Nurse/ Genetic Counsellors: Claire Kirk Diagnostic Laboratory Scientists: Mervyn Humphreys, Susan McNerlan

Birmingham (West Midlands Regional Genetics Service, Birmingham Women's NHS Foundation Trust, Birmingham Women's Hospital, Edgbaston, Birmingham, B15 2TG, UK)

Recruiting Consultant Clinical Geneticists: Louise Brueton, Trevor Cole*, Nicola Cooper, Helen Cox, Joanna Jarvis, Derek Lim, Jenny Morton, Andrew Norman, Chirag Patel, Nicola Ragge, Saba Sharif, Mark Tein, Julie Vogt, Denise Williams

Research Nurse/ Genetic Counsellors: Gail Kirby

Diagnostic Laboratory Scientists: David Bohanna, Kirsten McKay, Dominic J McMullan

Bristol (Bristol Genetics Service (Avon, Somerset, Gloucs and West Wilts), University Hospitals Bristol NHS Foundation Trust, St Michael's Hospital, St Michael's Hill, Bristol, BS2 8DT, UK) *Recruiting Consultant Clinical Geneticists*: Ruth Newbury-Ecob*, Sarah Smithson Research *Nurse/ Genetic Counsellors*: Rose Hawkins

Diagnostic Laboratory Scientists: Eileen Roberts, Christopher Wragg Cambridge (East Anglian Medical Genetics Service, Box 134, Cambridge University Hospitals NHS Foundation Trust, Cambridge Biomedical Campus, Cambridge ,CB2 0QQ, UK)

Recruiting Consultant Clinical Geneticists: Ruth Armstrong, Helen Firth*, Simon Holden, Sarju Mehta, Soo-Mi Park, Joan Paterson, Lucy Raymond, Richard Sandford, Geoff Woods

Research Nurse/ Genetic Counsellors: Jonathan Roberts, Sarah Wilcox

Diagnostic Laboratory Scientists: Ingrid Simonic, Becky Treacy Cardiff (Institute Of Medical Genetics, University Hospital Of Wales, Heath Park, Cardiff, CF14 4XW, UK and Department of Clinical Genetics, Block 12, Glan Clwyd Hospital, Rhyl, Denbighshire, LL18 5UJ, UK)

Recruiting Consultant Clinical Geneticists: Hayley Archer, Sally Davies, Dhavendra Kumar, Emma McCann*, Daniela T. Pilz*, Annie Procter

Research Nurse/ Genetic Counsellors: Karenza Evans Diagnostic

Laboratory Scientists: Sian Morgan, Hood Mugalaasi Dublin (National Centre for Medical Genetics, Our Lady's Children's Hospital, Crumlin, Dublin 12, Ireland)

Recruiting Consultant Clinical Geneticists: Sally Ann Lynch*

Research Nurse/ Genetic Counsellors: Rosie O'Shea Dundee (East of Scotland Regional Genetics Service, Human Genetics Unit, Pathology Department, NHS Tayside, Ninewells Hospital, Dundee, DD1 9SY, UK)

Recruiting Consultant Clinical Geneticists: Jonathan Berg*, David Goudie, Susann Schweiger Research Nurse/ Genetic Counsellors: Debbie Rice

Diagnostic Laboratory Scientists: David Baty, Norman Pratt Edinburgh (MRC Human Genetics Unit, MRC IGMM, University of Edinburgh, Western General Hospital, Edinburgh, EH4 2XU, UK)

Recruiting Consultant Clinical Geneticists: David R. FitzPatrick*, Wayne Lam, Anne Lampe *Research Nurse/ Genetic Counsellors*: Philip Greene

Diagnostic Laboratory Scientists: Eddy Maher, David Moore

Exeter (Peninsula Clinical Genetics Service, Royal Devon and Exeter NHS Foundation Trust, Clinical Genetics Department, Royal Devon & Exeter Hospital (Heavitree), Gladstone Road, Exeter, EX1 2ED, UK)

Recruiting Consultant Clinical Geneticists: Carole Brewer, Bruce Castle, Emma Kivuva*, Julia Rankin, Charles Shaw-Smith, Claire Turner, Peter Turnpenny

Research Nurse/ Genetic Counsellors: Gemma Devlin, Sarah Everest

Diagnostic Laboratory Scientists: Sian Ellard, Carolyn Tysoe Glasgow (West of Scotland Regional Genetics Service, NHS Greater Glasgow and Clyde, Institute Of Medical Genetics, Yorkhill Hospital, Glasgow, G3 8SJ, UK)

Recruiting Consultant Clinical Geneticists: Rosemarie Davidson, Carol Gardiner, Shelagh Joss, Esther Kinning, Victoria Murday, John Tolmie*, Margo Whiteford

Research Nurse/ Genetic Counsellors: Alexis Duncan

Diagnostic Laboratory Scientists: Gordon Lowther, Nicola Williams Leeds (Yorkshire Regional Genetics Service, Leeds Teaching Hospitals NHS Trust, Department of Clinical Genetics, Chapel Allerton Hospital, Chapeltown Road, Leeds, LS7 4SA, UK)

Recruiting Consultant Clinical Geneticists: Chris Bennett, Moira Blyth*, Emma Hobson, Alison Kraus, Katrina Prescott*, Audrey Smith, Jenny Thomson

Research Nurse/ Genetic Counsellors: Miranda Squires

Diagnostic Laboratory Scientists: Andrea Coates, Sarah Hewitt, Paul Roberts

Leicester (Leicestershire Genetics Centre, University Hospitals of Leicester NHS Trust, Leicester Royal Infirmary (NHS Trust), Leicester, LE1 5WW, UK)

Recruiting Consultant Clinical Geneticists: Pradeep Vasudevan*

Research Nurse/ Genetic Counsellors: Beckie Kaemba, Sandra Kazembe

Diagnostic Laboratory Scientists: Lara Cresswell

Liverpool (Merseyside and Cheshire Genetics Service, Liverpool Women's NHS Foundation Trust, Department of Clinical Genetics, Royal Liverpool Children's Hospital Alder Hey, Eaton Road, Liverpool, L12 2AP, UK)

Recruiting Consultant Clinical Geneticists: Astrid Weber*, Alan Fryer, Lynn Greenhalgh, Elizabeth Sweeney Research Nurse/ Genetic Counsellors: Gillian Roberts, Vivienne Sutton *Diagnostic Laboratory Scientists*: Angela Douglas, Una Maye

London - North West Thames (North West Thames Regional Genetics Centre, North West London Hospitals NHS Trust, The Kennedy Galton Centre, Northwick Park And St Mark's NHS Trust Watford Road, Harrow, HA1 3UJ, UK)

Recruiting Consultant Clinical Geneticists: Birgitta Bernhard, Angela Brady, Natalie Canham*, Neeti Ghali, Susan Holder, Anthony Vandersteen, Emma Wakeling

Research Nurse/ Genetic Counsellors: Cheryl Sequeira, Roldan Singzon

Diagnostic Laboratory Scientists: Louise Bourdon, Stewart Payne

London - Great Ormond Street (North East Thames Regional Genetics Service, Great Ormond Street Hospital for Children NHS Foundation Trust, Great Ormond Street Hospital, Great Ormond Street, London, WC1N 3JH, UK)

Recruiting Consultant Clinical Geneticists: Jane Hurst*, Melissa Lees, Elisabeth Rosser, Richard Scott

Research Nurse/ Genetic Counsellors: Kate Brunstrom, Georgina Hollingsworth

Diagnostic Laboratory Scientists: Lucy Jenkins, Jonathon Waters

London – Guy's (South East Thames Regional Genetics Centre, Guy's and St Thomas' NHS Foundation Trust, Guy's Hospital, Great Maze Pond, London, SE1 9RT, UK)

Recruiting Consultant Clinical Geneticists: Fiona Connell, Charu Deshpande, Frances Flinter, Melita Irving, Dragana Josifova, Shehla Mohammed*, Leema Robert

Research Nurse/ Genetic Counsellors: Tina Fendick, Caroline Langman

Diagnostic Laboratory Scientists: Caroline Ogilvie, Michael Yau

London - St George's (South West Thames Regional Genetics Centre, St George's Healthcare NHS Trust, St George's, University of London, Cranmer Terrace, London, SW17 0RE, UK) *Recruiting Consultant Clinical Geneticists*: Frances Elmslie, Tessa Homfray, Sahar Mansour*, Meriel McEntagart, Anand Saggar, Kate Tatton-Brown

Research Nurse/ Genetic Counsellors: Uruj Anjum

Diagnostic Laboratory Scientists: Karen Marks, Rohan Taylor

Manchester (Manchester Centre for Genomic Medicine, St Mary's Hospital, Central Manchester University Hospitals NHS Foundation Trust, Manchester Academic Health Science Centre, Manchester M13 9WL)

Recruiting Consultant Clinical Geneticists: Kate Chandler, Jill Clayton-Smith*, Yanick Crow, Elizabeth Jones, Bronwyn Kerr, Kay Metcalfe

Research Nurse/ Genetic Counsellors: Carina Donnelly, Zara Skitt

Diagnostic Laboratory Scientists: Lorraine Gaunt, Emma Miles Newcastle (Northern Genetics Service, Newcastle upon Tyne Hospitals NHS Foundation Trust, Institute of Human Genetics, International Centre for Life, Central Parkway, Newcastle upon Tyne, NE1 3BZ, UK)

Recruiting Consultant Clinical Geneticists: John Burn, Richard Fisher, Judith Goodship, Alex Henderson, Tara Montgomery, Miranda Splitt*, Michael Wright

Research Nurse/ Genetic Counsellors: Linda Sneddon

Diagnostic Laboratory Scientists: David Bourn, Stephen Hellens

Nottingham (Nottingham Regional Genetics Service, City Hospital Campus, Nottingham University Hospitals NHS Trust, The Gables, Hucknall Road, Nottingham NG5 1PB, UK) *Recruiting Consultant Clinical Geneticists*: Abhijit Dixit, Jacqueline Eason*, Ajoy Sarkar, Nora Shannon, Mohnish Suri

Research Nurse/ Genetic Counsellors: Ann Selby

Diagnostic Laboratory Scientists: Gareth Cross, Katherine Martin Oxford (Oxford Regional Genetics Service, Oxford Radcliffe Hospitals NHS Trust, The Churchill Old Road, Oxford, OX3 7LJ, UK)

Recruiting Consultant Clinical Geneticists: Edward Blair, Richard Gibbons, Usha Kini*, Sue Price, Debbie Shears, Helen Stewart

Research Nurse/ Genetic Counsellors: Julie Phipps, Abigail Pridham, Hellen Purnell

Diagnostic Laboratory Scientists: Susan Clasper, Anneke Seller Sheffield (Sheffield Regional Genetics Services, Sheffield Children's NHS Trust, Western Bank, Sheffield, S10 2TH, UK) *Recruiting Consultant Clinical Geneticists*: Meena Balasubramanian, Diana Johnson, Michael Parker*

Research Nurse/ Genetic Counsellors: Louise Nevitt, Stuart Ingram, Cat Taylor

Diagnostic Laboratory Scientists: Emma Shearing, Kath Smith

Southampton/Wessex (Wessex Clinical Genetics Service, University Hospital Southampton, Princess Anne Hospital, Coxford Road, Southampton, SO16 5YA, UK and Wessex Regional Genetics Laboratory, Salisbury NHS Foundation Trust, Salisbury District Hospital, Odstock Road, Salisbury, Wiltshire, SP2 8BJ, UK and Faculty of Medicine, University of Southampton)

Recruiting Consultant Clinical Geneticists: Munaza Ahmed, Diana Baralle, Amanda Collins, Nicola Foulds, Katherine Lachlan, I. Karen Temple*, Diana Wellesley

Research Nurse/ Genetic Counsellors: Lucy Harrison, Audrey Torokwa Diagnostic Laboratory scientists: David J. Bunyan, Morag N. Collinson

The Synaptopathies and Paroxysmal Syndromes (SYNaPS) Study Group

(http://neurogenetics.co.uk/synaptopathies-synaps/)

Study Group Members:

Vincenzo Salpietro[1], Stephanie Efthymiou[2], Yamna Kriouile[3], Mohamed El Khorassani[3], Mhammed Aguennouz[3], Blagovesta Karashova[4], Daniela Avdjieva[4], Hadil Kathom[4], Radka Tincheva[4], Lionel Van Maldergem[5], Wolfgang Nachbauer[6], Sylvia Boesch[6], Larissa Arning[7], Dagmar Timmann[8], Bru Cormand[9], Belen Pérez-Dueñas[10], Gabriella Di Rosa[11], Erica Pironti[11], Jatinder S. Goraya[12], Tipu Sultan[13], Salman Kirmani[14], Shahnaz Ibrahim[15], Farida Jan[15], Jun Mine[16], Selina Banu[17], Pierangelo Veggiotti[18], Michel D. Ferrari[19], Alberto Verrotti[20], Gian Luigi Marseglia[21], Salvatore Savasta[21], Barbara Garavaglia[22], Carmela Scuderi[23], Eugenia Borgione[23], Valeria Dipasquale[24], Maria Concetta Cutrupi[24], Simona Portaro[25], Benigno Monteagudo Sanchez[26], Mercedes Pineda-Marfa'[27], Francina Munell[27], Alfons Macaya[27], Richard Boles[28], Gali Heimer[29], Savvas Papacostas[30], Andreea Manole[1], Nancy Malintan[1], Maria Natalia Zanetti[1], Michael G. Hanna[1], James E. Rothman[1,31], Dimitri M. Kullmann[2], Henry Houlden[1]

Study Group Members Affiliations

[1]Department of Molecular Neuroscience, UCL Institute of Neurology, London WC1N 3BG, UK

[2]Department of Clinical and Experimental Epilepsy, UCL Institute of Neurology, London WC1N 3BG, UK

[3]Children's Hospital of Rabat, University of Rabat, Rabat 6527, Morocco

[4]Department of Paediatrics, Medical University of Sofia, Sofia 1431, Bulgaria

[5]Centre of Human Genetics, University Hospital Liege, Liege 4000, Belgium

[6]Department of Neurology, Medical University Innsbruck, Anichstrasse 35, Innsbruck 6020, Austria

[7]Department of Human Genetics, Ruhr-University Bochum, Bochum 44801, Germany

[8]Braun Neurologische Universitätsklinik Universität *Essen,* Hufelandstr 55, *Essen* D-45122, Germany

[9]Department of Genetics, Universitat de Barcelona, Barcelona 08007, Spain

[10]Hospital Sant Joan de Deu, Esplugues de Llobregat 08950, Barcelona, Spain

[11]Department of Pediatrics, University of Messina, Messina 98123, Italy

[12]Division of Paediatric Neurology, Dayanand Medical College & Hospital, Ludhiana, Punjab 141001, India

[13]Department of Paediatric Neurology, Children's Hospital of Lahore, Lahore 381-D/2, Pakistan

[14]Department of Medical Genetics, Aga Khan University Hospital, Karachi, Karachi City, Sindh 74800,

Pakistan

[15]Department of Paediatric Neurology, Aga Khan University Hospital, Karachi, Karachi City, Sindh 74800, Pakistan

[16]Department of Pediatrics, Shimane University School of Medicine, 89-1 Enya, Izumo, Shimane 693-8501, Japan

[17] Institute of Child Health and Shishu Shastho Foundation Hospital, Mirpur, Dhaka 1216, Bangladesh

[18]Pediatric Neurology Unit, V. Buzzi Children's Hospital, Via Castelvetro 32, 20154 Milan, Italy

[19]Leiden University Medical Center, Albinusdreef 2, Leiden 2333, Netherlands

[20]Paediatric Department, San Salvatore Hospital, University of L'Aquila, L'Aquila, Italy

[21]Department of Pediatrics, University of Pavia, IRCCS Policlinico "San Matteo", Pavia 27100, Italy

[22]IRCCS Foundation, Neurological Institute “Carlo Besta”, Molecular Neurogenetics, 20126 Milan, Italy

[23]Laboratorio di Neuropatologia Clinica, U.O.S. Malattie, Neuromuscolari Associazione OASI Maria SS. ONLUS – IRCCS, Via Conte Ruggero 73, 94018 Troina, Italy

[24]Department of Pediatrics, University Hospital “Gaetano Martino”, University of Messina, Messina 98123, Italy

[25]IRCCS Centro Neurolesi "Bonino Pulejo", SS113, c.da Casazza, 98124 Messina, Italy

[26]Hospital Arquitecto Marcide, Avenida de la Residencia S/N, Ferrol (A Coruña), 15401 Spain

[27]Neuropediatrics Unit, University Hospital Vall d'Hebron, Barcelona 08035, Spain

[28]Courtagen Life Sciences, 12 Gill Street Suite 3700, Woburn, MA 01801 USA

[29]Division of Pediatric Neurology, Edmond and Lily Children's Hospital, Chaim Sheba Medical Center, 52621 Ramat Gan, Israel

[30]The Cyprus Institute of Neurology and Genetics, 1683 Nicosia, Cyprus

[31]Department of Cell Biology, Yale University School of Medicine, New Haven, USA

Supplemental References

[1] Engel J., Jr. (2001). A proposed diagnostic scheme for people with epileptic seizures and with epilepsy: report of the ILAE task force on classification and terminology. Epilepsia. *42*, 796–803
[2] B, Hanefeld F, Percy A, Skjeldal O. (2002). An update on clinically applicable diagnostic criteria in Rett syndrome. Comments to Rett Syndrome Clinical Criteria Consensus Panel Satellite to European Paediatric Neurology Society Meeting, Baden Baden, Germany, 11 September 2001. Eur. J. Paediatr. Neurol. *6*, 293–297
[3] Neul JL, Kaufmann WE, Glaze DG, Christodoulou J, Clarke AJ, Bahi-Buisson N, Leonard H, Bailey ME, Schanen NC, Zappella M, et al. (2010). Rett syndrome: revised diagnostic criteria and nomenclature. Ann. Neurol. *68*, 944-50
[4] Yoo Y, Jung J, Lee YN, Lee Y, Cho H, Na E, Hong J, Kim E, Lee JS, Lee JS, et al. (2017). GABBR2 mutations determine phenotype in rett syndrome and epileptic encephalopathy. Ann. Neurol. *82*, 466-478.
[5] Srivastava S, Desai S, Cohen J, Smith-Hicks C, Barañano K, Fatemi A, Naidu S. (2018). Monogenic disorders that mimic the phenotype of Rett syndrome. Neurogenetics. *19*, 41-47
[6] Schalock, R.L., Buntinx, W.H.E., Borthwick-Duffy, S., Bradley, V., Craig, E.M., Coulter, Craig EM, Gomez SC, Lachapelle Y, Luckasson R, et al. (2010). Intellectual disability: Definition, classification, and system of supports *(11e).*
[7] Sobreira N, Schiettecatte F, Boehm C, Valle D, Hamosh A. (2015). New tools for Mendelian disease gene identification: PhenoDB variant analysis module; and GeneMatcher, a web-based tool for linking investigators with an interest in the same gene. Hum. Mutat. *36*, 425-31.

[8] Huang Z, Sun Y, Fan Y, Wang L, Liu H, Gong Z, Wang J, Yan H, Wang Y, Hu G, et al. (2018). Genetic Evaluation of 114 Chinese Short Stature Children in the Next Generation Era: a Single Center Study. Cell. Physiol. Biochem. *49*, 295-305

[9] Martín-Hernández E, Rodríguez-García ME, Chen CA, Cotrina-Vinagre FJ, Carnicero-Rodríguez P, Bellusci M, Schaaf CP, Martínez-Azorín F. (2018). Mitochondrial involvement in a Bosch-Boonstra-Schaaf optic atrophy syndrome patient with a novel de novo NR2F1 gene mutation. J. Hum. Genet. *63*, 525-528.

[10] Martín-Hernández E, Rodríguez-García ME, Camacho A, Matilla-Dueñas A, García-Silva MT, Quijada-Fraile P, Corral-Juan M, Tejada-Palacios P, de Las Heras RS, Arenas J, et al. (2016). New ATP8A2 gene mutations associated with a novel syndrome: encephalopathy, intellectual disability, severe hypotonia, chorea and optic atrophy. Neurogenetics. *17*, 259-263.

[11] Salpietro V, Lin W, Delle Vedove A, Storbeck M, Liu Y, Efthymiou S, Manole A, Wiethoff S, Ye Q, Saggar A, et al. (2017). Homozygous mutations in VAMP1 cause a presynaptic congenital myasthenic syndrome
Ann. Neurol. *81*, 597-603.

[12] Mencacci NE, Kamsteeg EJ, Nakashima K, R'Bibo L, Lynch DS, Balint B, Willemsen MA, Adams ME, Wiethoff S, Suzuki K, et al. (2016). De-Novo Mutations in PDE10A Cause Childhood-Onset Chorea with Bilateral Striatal Lesions. Am. J. Hum. Genet. *98*, 763-71.

[13] Melia TJ, Weber T, McNew JA, Fisher LE, Johnston RJ, Parlati F, Mahal LK, Sollner TH, Rothman JE. (2002). Regulation of membrane fusion by the membrane-proximal coil of the t-SNARE during zippering of SNAREpins. J. Cell. Biol. *158,* 929-40

[14] Weber T, Zemelman BV, McNew JA, Westermann B, Gmachl M, Parlati F, Söllner TH, Rothman JE. (1998). SNAREpins: minimal machinery for membrane fusion. Cell. *92*, 759-72.

[15] Shen J, Tareste DC, Paumet F, Rothman JE, Melia TJ. (2007). Selective activation of cognate SNAREpins by Sec1/Munc18 proteins. Cell. *128*, 183-95

[16] Bradford MM. (1976). A rapid and sensitive method for the quantitation of microgram quantities of protein utilizing the principle of protein-dye binding. Anal Biochem. *72*, 248-54.

[17] Walch-Solimena C, Blasi J, Edelmann L, Chapman ER, von Mollard GF, Jahn R. (1995). The t-SNAREs syntaxin 1 and SNAP-25 are present on organelles that participate in synaptic vesicle recycling. J Cell. Biol. *128*, 637-45.

[18] Takamori S, Holt M, Stenius K, Lemke EA, Grønborg M, Riedel D, Urlaub H, Schenck S, Brügger B, Ringler P, Müller SA, Rammner B, Gräter F, Hub JS, De Groot BL, Mieskes G, Moriyama Y, Klingauf J, Grubmüller H, Heuser J, Wieland F, Jahn R. (2006). Molecular anatomy of a trafficking organelle. Cell. *127,* 831-46

[19] Struck DK, Hoekstra D, Pagano RE. (1981). Use of resonance energy transfer to monitor membrane fusion. Biochemistry. *20*, 4093-9.

[20] Scott BL, Van Komen JS, Liu S, Weber T, Melia TJ, McNew JA. Liposome fusion assay to monitor intracellular membrane fusion machines. (2003). Methods Enzymol. *372,* 274-300.

[21] Stein A, Weber G, Wahl MC, Jahn R. (2009). Helical extension of the neuronal SNARE complex into the membrane. Nature. *460,* 525-8.

[22] Bussi G, Donadio D, Parrinello M. (2007). Canonical sampling through velocity rescaling. J. Chem. Phys. *126*, 014101.

[23] Hess B. (2008). P-LINCS: A Parallel Linear Constraint Solver for Molecular Simulation. J. Chem. Theory. Comput. *4,* 116-22.

[24] Pronk S, Páll S, Schulz R, Larsson P, Bjelkmar P, Apostolov R, Shirts MR, Smith JC, Kasson PM, van der Spoel D, Hess B, Lindahl E.(2013). GROMACS 4.5: a high-throughput and highly parallel open source molecular simulation toolkit. Bioinformatics. *29*, 845-54
